# Barium silicate nanoparticles, an efficient catalyst for one-pot green synthesis of α-benzyl amino coumarin derivatives as potential chemotherapeutic agents†

**DOI:** 10.1039/d3ra00796k

**Published:** 2023-07-12

**Authors:** Hadi Taghrir, Zeinab Faghih, Majid Ghashang, Leila Emami, Shadi Dalili, Soghra Khabnadideh

**Affiliations:** a Pharmaceutical Sciences Research Center, Shiraz University of Medical Sciences Shiraz Islamic Republic of Iran Faghihl@sums.ac.ir layafaghih@gmail.com +98-7132424126 +98-7132424127; b Department of Chemistry, Najafabad Branch, Islamic Azad University Najafabad Iran ghashangmajid@pmt.iaun.ac.ir +98-3142291016 +98-3142291004; c Department of Physical and Environmental Sciences 1265 Military Trail Scarborough ON M1C 1A4 Canada

## Abstract

A new, simple, and efficient method for synthesis of α-benzyl amino coumarin and its derivatives (1–24) is described *via* a one-pot, three-component condensation of aromatic aldehydes, amine, and 4-hydroxycoumarin under green chemistry conditions: water as a solvent and BaSiO_3_ nanoparticles as catalyst. BaSiO_3_ nanoparticles and all synthesized derivatives were characterized by multiple methods including; XRD, NMR, and FE-SEM. This method which gives higher yields, is also less expensive, and more environmentally friendly compared with other methods in the literature. *In silico* physicochemical and pharmacokinetics analyses were done on all synthesized compounds and indicated that these α-benzyl amino coumarins would be effective scaffolds for the future development of chemotherapeutic agents.

## Abstract 2

A new, simple, and efficient method for synthesis of α-benzyl amino coumarin and its derivatives (1–24) *via* a one-pot reaction and, their appropriate ADMET properties.

## Graphical abstract

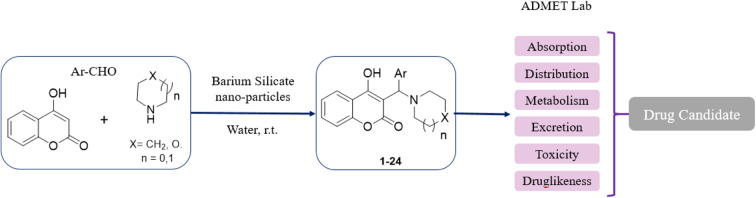

## 1. Introduction

Recently, 4-hydroxycoumarin and its derivatives have been of great interest due to their special role in natural and synthetic organic chemistry.^1^ Furthermore, coumarin is a suitable scaffold for chemical reactions in several studies, due to its hydroxyl and carbonyl groups adjacent to the α-position. Consequently, the 4-hydroxycoumarin and its derivatives have broad spectrum pharmacological properties including: antioxidant,^2^ antimicrobial,^3^ HIV protease inhibitors,^4^ antibacterial,^5^ antifungal,^6^ antitumor,^7^ and anticoagulant^8^ activities. Thus, the development of coumarin-containing compounds for drug discovery has recently drawn much attention. α-Benzyl amino coumarins are one of the most useful products resulting from 4-hydroxycoumarin's reactions. These compounds have been developed with various methods such as using: non-ionic surfactant in aqueous media,^9^ catalyst-free conditions in dichloromethane,^10^ InCl_3_ in toluene,^11,12^ chlorosulfonic acid,^13^ TiO_2_ nanocatalyst,^14^ catalyst-free in ethanol solvent,^15^ {[1,4-DHPyrazine][C(CN)_3_]_2_} as a new nanostructured molten salt (NMS) catalyst,^16^ catalyst-free in CH_2_Cl_2_ (ref. 17), [Et_3_NH][HSO_4_] ionic liquid,^18^ Fe_3_O_4_@ZrO_2_/SO_4_^−2^ heterogeneous nanocatalyst in CH_3_CN,^19^ β-cyclodextrin based nanosponge in ethanol,^20^ free-catalyst in CH_3_CN,^21^ nano crystalline ZnO,^22^ NaOH.^23^ However, most of these methods rely on multistep reactions and complex synthetic pathways with prolonged reaction times, low yields, expensive and harsh reaction conditions, as well as toxic catalysts. Thus, the development of new methods without such limitations is of great importance to prepare such heterocycles, found in natural products. Furthermore, multicomponent reactions (MCRs) are generating considerable interest in pharmaceutical chemistry to synthesize diverse structures of bioactive heterocycles, involving three or more reactants in one-pot reactions.^24^ Due to non-toxic and unique nature of water, there has been a rapid rise in the use of aqueous environment instead of conventional organic solvents in organic synthesis, especially in the MCRs.

**Scheme 1:**
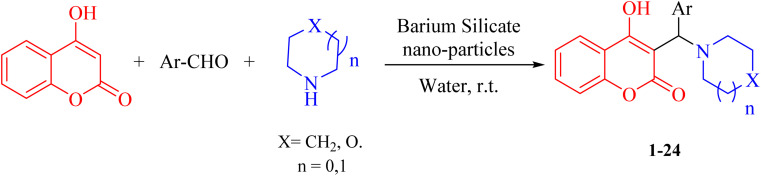
Synthesis of α-benzyl amino coumarin derivatives.

In the present study, due to the importance of α-benzyl amino coumarin scaffolds as a significant class of heterocycles in medicinal chemistry, a new, highly efficient, mild, and eco-friendly synthesis protocol is reported. Condensation between 4-hydroxycoumarin, an aliphatic amine, and a vast range of aromatic aldehydes using barium silicate nanoparticles as a heterogeneous, effective catalyst, is applied to obtain α-benzyl amino coumarin compounds (Scheme 1).

Furthermore, regarding the significant biological profiles of the coumarin-bearing structures, the physicochemical and pharmacokinetic properties of these α-benzyl amino coumarins were examined through *in silico* analysis, to identify them as suitable biological candidates for future evaluations.

## 2. Result and discussion

### 2.1. Chemistry

**Fig. 1:**
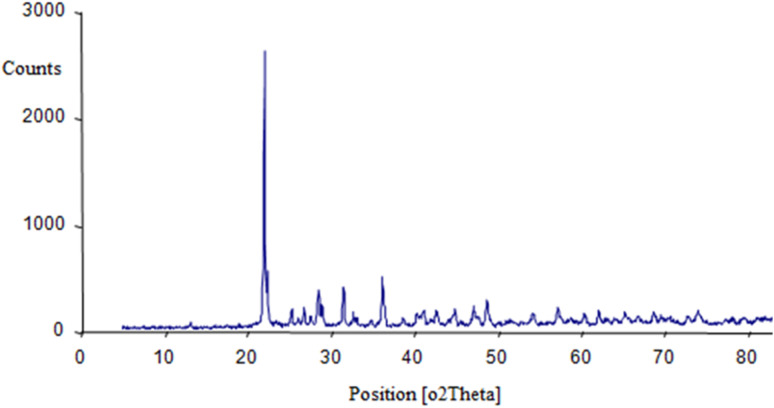
XRD pattern of BaSiO_3_ Nanoparticles.

In order to determine the crystalline structure and phase composition of the BaSiO_3_ nanopowder, X-ray diffraction (XRD) analysis using Cu-Kα radiation (Fig. 1). The average size was measured by the Sherer equation by measuring the highest peak (peak appearing at 22.0° (2° theta)); the XRD spectrum was 30 nm.

**Fig. 2:**
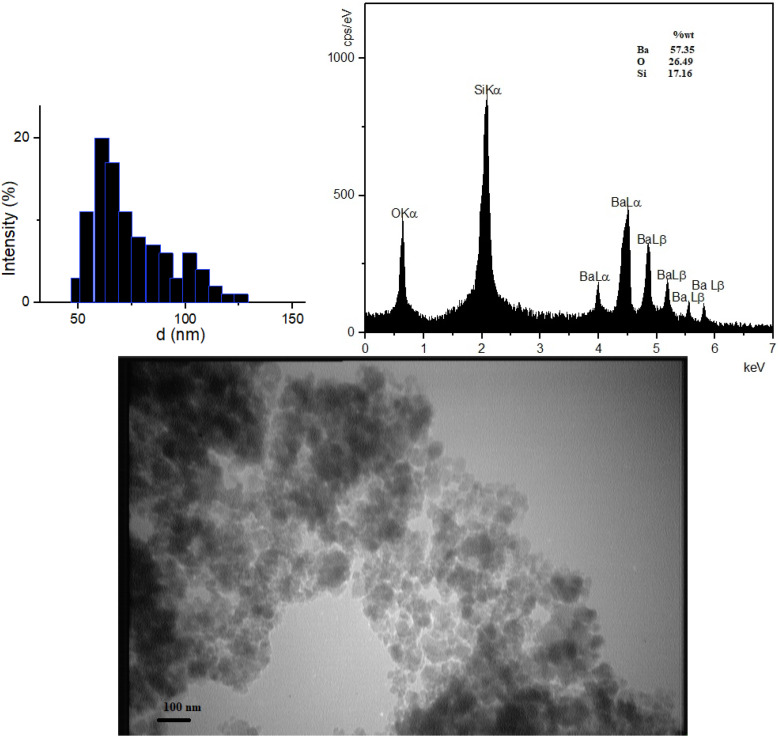
DLS, EDS analyses, and TEM photographs of BaSiO_3_ nanoparticles.

A particle size distribution diagram was plotted through the data obtained from dynamic light scattering (DLS) analysis and showed the particles were dispersed in the range of 45–140 nm. According to the diagram, the average particle size was 74 nm (Fig. 2). The energy-dispersive X-ray spectroscopy analysis (EDAX) demonstrated the elements in the composition and the weight percentage of each (17.16% Si, 57.35% Ba, and 26.49% O), which confirmed the proposed structure of the composition as BaSiO_3_. The TEM Photograph displayed unregular and chaotic particles with nanometer size (Fig. 2).

**Fig. 3:**
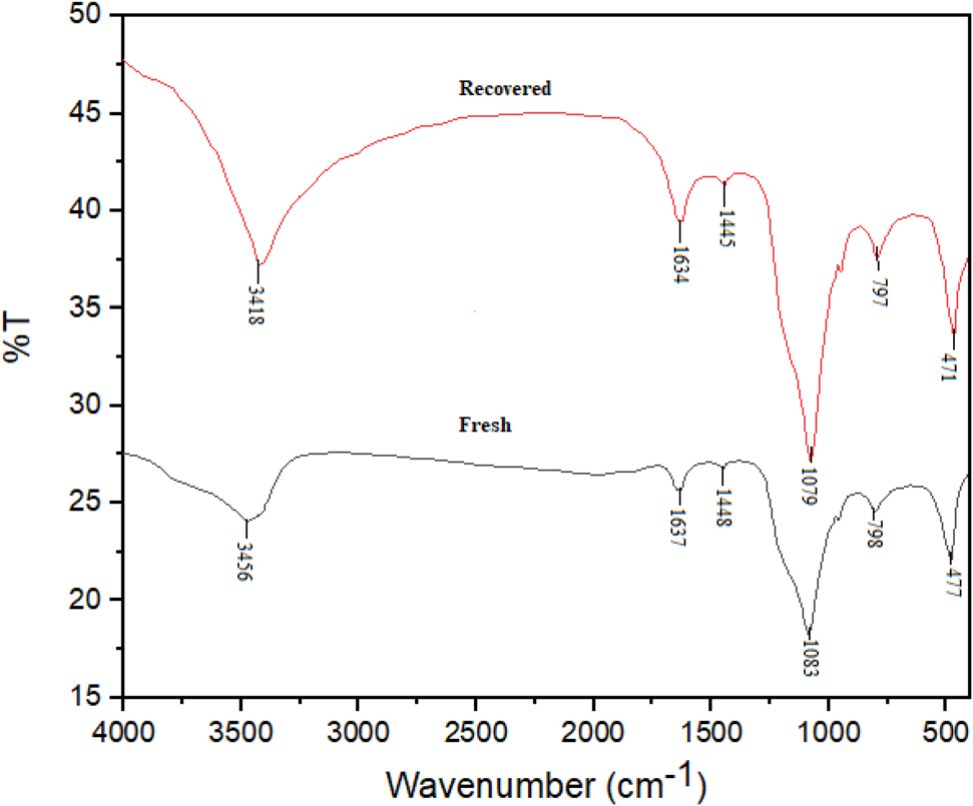
FT-IR analysis of BaSiO_3_ nanoparticles.

Fig. 3, represented the FT-IR spectra of fresh and recovered BaSiO_3_ which was used for the evaluation of functional groups presented in the structure of the catalyst. The samples contained metal–oxygen bonds which showed stretching and bending vibrations. The broad adsorption peak which appeared in the 3200–3700 cm^−1^ was corresponded to stretching vibrations of Si–OH and –OH groups presented in BaSiO_3_ and adsorbed water. The adsorption peaks at 1637, 1448, and 1083 cm^−1^ could be assigned to the presence of Si–O (stretching vibration), and Si–O–Si (asymmetric stretching vibration) bonds. The peaks entered at 798 and 477 cm^−1^ were assigned to be related to Si–O–Ba stretching vibration and Ba–O bonds (Fig. 3).

To find the optimal conditions for the synthesis of α-benzyl amino coumarins, the synthesis of 4-hydroxy-3-(phenyl(piperidine-1-yl)methyl)-2*H*-chromen-2-one (compound A_1_), by the reaction of benzaldehyde (1.2 mmol), pyridine (1.2 mmol) and 4-hydroxy-coumarin (1 mmol), was selected as a model reaction.

This reaction was performed in water as a green solvent, as well as in the presence of various organic solvents, using 0.56 mg of barium silicate nanoparticle (1–8) (Table 1).

**Table 1:** Optimization of the reaction conditions in the synthesis of 4-hydroxy-3-(phenyl(piperidine-1-yl)methyl)-2*H*-chromium-2-one^a^

Entry	Catalyst (mmol)	*T* (°C)	Solvent (5 ml)	Yield^b^ (%)
1	0.56	r.t.	—	10
2	0.56	r.t.	*n*-Hexane	—
3	0.56	r.t.	CH_2_Cl_2_	—
4	0.56	r.t.	Et_2_O	—
5	0.56	r.t.	EtOAc	—
6	0.56	r.t.	EtOH	10
7	0.56	r.t.	MeOH	40
8	0.56	r.t.	H_2_O	79
9	0.14	r.t.	H_2_O	51
10	0.28	r.t.	H_2_O	85
11	1	r.t.	H_2_O	85

a Time for reaction: 2 hours.

b Isolated yield.

**Scheme 2:**
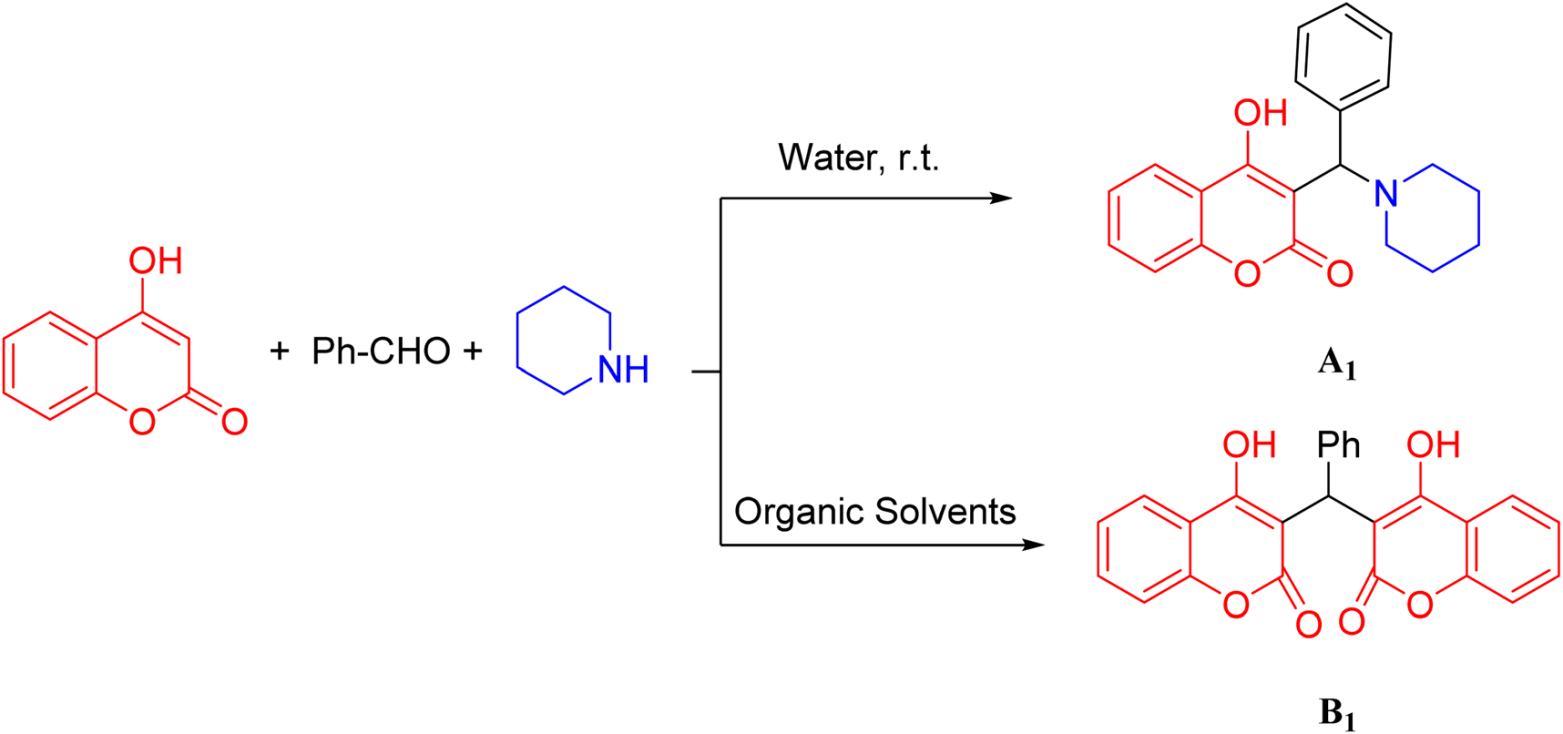
Multi-component reaction of benzaldehyde, piperidine, and 4-hydroxycomarin in water and organic solvents.

As seen from the results represented in Table 1, when the model reaction was carried out in the presence of organic solvents, it did not lead to the production of A_1_ products but rather led to the formation of 3,3′-(phenyl methylene) bis(4-hydroxy-2*H*-chromen-2-one) (B_1_) as a final product. This finding showed that A products were only obtained in highly aqueous media. (Scheme 2). The different amounts of barium silicate nanoparticle in the model reaction were also investigated (8–11), which indicated that using 0.28 mg of this catalyst increased the yield of the reaction properly.

Overall, the use of 0.28 mg of designed catalysts in aqueous media was selected as an optimal condition for the model reaction to synthesize various derivatives of α-benzyl amino coumarin compounds using cycloaliphatic amines, different aromatic and aliphatic aldehydes, A_1_–A_30_ (Table 2). As shown in Table 2, unfortunately, the aliphatic aldehydes were unreactive and could not be matched for the given reaction to form targeted molecules. Only aromatic aldehydes generated the desired compounds, A_1_–A_24_ (Table 2).

**Table 2:** Synthesis of α-benzyl amino coumarin derivatives using barium silicate nanoparticles

Entry	Aldehyde	Amine	Product	Time (min)	Yield^a^ (%)
1	Benzaldehyde	Piperidine	A_1_	30	85
2	2-Chlorobenzaldehyde	Piperidine	A_2_	60	87
3	4-Chlorobenzaldehyde	Piperidine	A_3_	40	70
4	2-Methylbenzaldehyde	Piperidine	A_4_	70	84
5	4-Methylbenzaldehyde	Piperidine	A_5_	60	81
6	4-Methoxybenzaldehyde	Piperidine	A_6_	70	70
7	4-*tert*-Butylbenzaldehyde	Piperidine	A_7_	40	89
8	3-Nitrobenzaldehyde	Piperidine	A_8_	25	90
9	2,4-Dichlorobenzaldehyde	Piperidine	A_9_	75	61
10	4-Bromobenzaldehyde	Piperidine	A_10_	40	90
11	Benzaldehyde	Pyrrolidine	A_11_	45	82
12	4-Bromobenzaldehyde	Pyrrolidine	A_12_	30	90
13	4-Chlorobenzaldehyde	Pyrrolidine	A_13_	30	93
14	4-Nitrobenzaldehyde	Pyrrolidine	A_14_	25	88
15	4-Methylbenzaldehyde	Pyrrolidine	A_15_	65	89
16	4-Methoxybenzaldehyde	Pyrrolidine	A_16_	80	95
17	3-Nitrobenzaldehyde	Pyrrolidine	A_17_	30	92
18	Benzaldehyde	Morpholine	A_18_	45	93
19	4-Bromobenzaldehyde	Morpholine	A_19_	40	90
20	4-Chlorobenzaldehyde	Morpholine	A_20_	35	98
21	4-Nitrobenzaldehyde	Morpholine	A_21_	30	78
22	4-Methylbenzaldehyde	Morpholine	A_22_	55	90
23	2,4-Dichlorobenzaldehyde	Morpholine	A_23_	45	92
24	3,5-Dchlorobenzaldehyde	Morpholine	A_24_	30	89
25	Butyraldehyde	Piperidine	A_25_	120	—
26	Butyraldehyde	Pyrrolidine	A_26_	120	—
27	Butyraldehyde	Morpholine	A_27_	120	—
28	Hexanal	Piperidine	A_28_	120	—
29	Hexanal	Pyrrolidine	A_29_	120	—
30	Hexanal	Morpholine	A_30_	120	—

a Isolated yield.

**Scheme 3:**
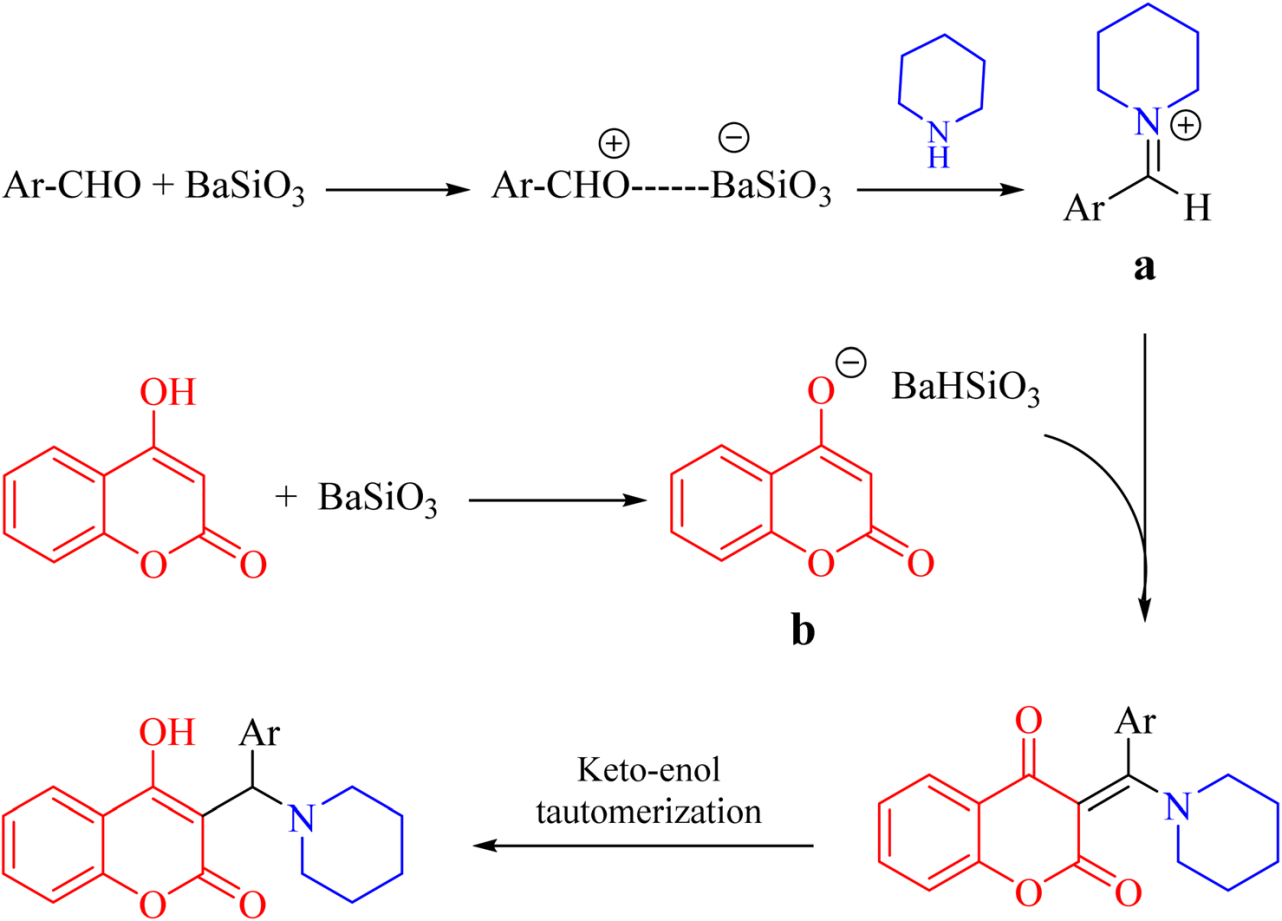
Proposed mechanism of the reaction for the synthesis of α-benzyl amino coumarin derivatives.

According to our observations, it is believed that the reaction could be started by the activation of aldehyde using Lewis acid characteristics of BaSiO_3_ and subsequently formation of iminium ion intermediate (a) from the reaction of secondary amine with the activated aldehyde. In addition, 4-hydroxycoumarin could be equilibrated with their enolate ion form (b). The final product would be achieved through the reaction of a and b and subsequent keto–enol tautomerization as shown in Scheme 3.

**Fig. 4:**
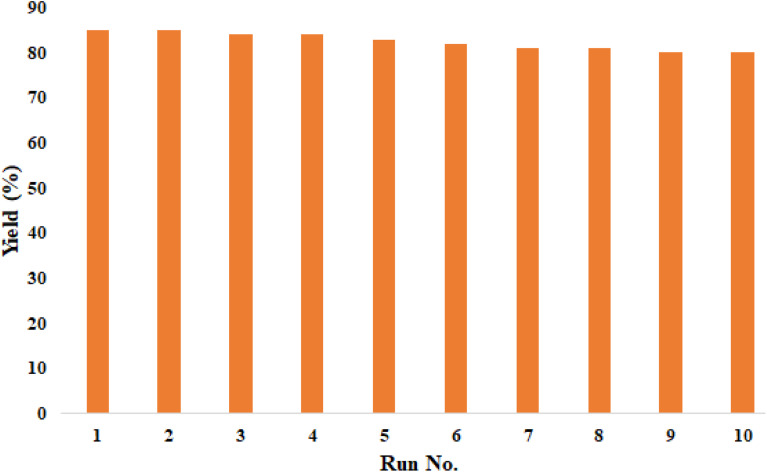
The recyclability test results of BaSiO_3_.

The catalyst recyclability also was investigated in the synthesis of product A_1_. In each step, the compound separated from the reaction mixture was washed with acetone and dried at 50 °C. The results of 10 recycling and reuse of the catalyst are shown in Fig. 4. The obtained results show the good recyclability of the catalyst and its good stability. The FT-IR spectrum (Fig. 3) of the recovered catalyst confirms the structural stability of the compound.

Therefore, based on the purpose of this study, efficacy, generality, and the use of barium silicate as an efficient catalyst for the preparation of various α-benzyl amino coumarin derivatives was investigated by using different aromatic aldehydes containing electron-donating or electron-withdrawing groups (Scheme 1). The results are summarized in Table 2.

### 2.2. *In silico* ADMET modeling studies

The ADME properties have a significant role in the acceptance or rejection of compounds as potential drug candidates. In addition, toxicity is another property that can damage the organism. Hence, the study of ADMET properties of synthesized compounds before biological evaluation is necessary. The Swiss ADME software was utilized to predict the physiochemical properties of the synthesized compounds (1–24) according to Lipinski and Veber's rules. The molecular weights (MW) of all compounds were in the acceptable range (321–416 g mol^−1^). The hydrogen bond properties (as donors or acceptors), total polar surface area (TPSA), and the rotatable bond number value of all synthesized compounds were reasonable. Moreover, all of the compounds had admissible lipophilicity (logP) values. Based on these physiochemical properties, all of the α-benzyl amino coumarin derivatives represented good potential for oral bioavailability (Table 3).

**Table 3:** Physiochemical properties of all α-benzyl amino coumarin derivatives (1–24)

Entry	MW^a^	LogP^b^	HBD^c^	HBA^d^	TPSA^e^ (Å)	RB^f^	Lipinski/Veber violation
1	335.4	3.04	1	4	53.68	3	0
2	369.84	3.62	1	4	53.68	3	0
3	369.84	3.52	1	4	53.68	3	0
4	349.42	3.26	1	4	53.68	3	0
5	349.42	3.26	1	4	53.68	3	0
6	365.42	2.69	1	5	62.91	4	0
7	391.50	3.89	1	4	53.68	4	0
8	380.39	2.08	1	6	99.5	4	0
9	404.29	4.01	1	4	53.68	3	0
10	414.29	3.63	1	4	53.68	3	0
11	321.37	2.381	1	4	53.68	3	0
12	400.27	3.41	1	4	53.68	3	0
13	355.81	3.30	1	4	53.68	3	0
14	366.37	1.85	1	6	99.50	4	0
15	335.40	3.04	1	4	53.68	3	0
16	351.40	2.48	1	5	62.91	4	0
17	366.67	1.85	1	6	99.50	4	0
18	337.37	1.93	1	5	62.91	3	0
19	416.27	2.59	1	5	62.91	3	0
20	371.81	2.48	1	5	62.91	3	0
21	382.37	1.06	1	7	108.73	4	0
22	351.40	2.21	1	5	62.91	3	0
23	406.26	2.96	1	5	62.91	3	0
24	402.26	2.96	1	5	62.91	3	0
Lipinski/Veber's rules	≤ 500	≤ 5	≤ 5	≤ 10	≤ 140	≤ 10	≤ 1

a Molecular weight (MW).

b Logarithm of partition coefficient between *n*-octanol and water (logP).

c Number of hydrogen bond donors (HBD).

d Number of hydrogen bond acceptors (HBA).

e Topological polar surface area (TPSA).

f Number of rotatable bonds (RB).

The absorption and distribution properties of the synthesized compounds are displayed in Table 4. The log *S* value shows the water solubility, which is in the range of −3.63 to −2.18 for all tested compounds. In fact, their lower log *S* values represented better solubility. The human intestinal absorptions (% HIA) were above 95, which indicated high intestinal absorption in the transport of oral drugs to their biological targets. The Caco-2 permeability of all compounds except analogue 21, illustrated that they exhibit permeability across intestinal membranes. In the case of blood–brain barrier property, compounds 6, 8, 14, 16–17, and 19–22 showed low blood–brain barrier percentages, respectively, leading to a lower neurological effect. Table 4 indicates that all of the tested compounds had no effects on P-glycoprotein efflux transporters as both substrate and inhibitor, therefore, these systems do not disturb the cell permeability and absorption of the compounds. The binding of drug molecules to plasma proteins can affect their bioavailability and distribution across cell membranes. Thus, the percentages of plasma protein binding (PPB) of all compounds was also calculated. All of them had weak PPB with values lower than 90%, except for 3,9, 12, and 19 which showed strong binding to plasma proteins.^26^

**Table 4:** Absorption and distribution profile of all α-benzyl amino coumarin compounds^a^

Entry	HIA	Caco-2 permeability	BBB	P-Glycoprotein	Plasma protein bonding (PPB)
1	95.89	40.71	0.53	NS/NI	84.19
2	96.35	32.78	1.36	NS/NI	88.44
3	96.35	33.20	1.45	NS/NI	95.61
4	95.99	42.25	1.07	NS/NI	84.00
5	95.99	41.68	1.27	NS/NI	84.18
6	95.94	43.59	0.18	NS/NI	83.07
7	96.28	46.85	3.17	NS/NI	86.23
8	96.85	21.52	0.01	NS/NI	88.55
9	96.73	36.52	3.21	NS/NI	98.25
10	96.04	44.79	1.02	NS/NI	85.61
11	95.79	36.93	0.89	NS/NI	78.15
12	96.55	29.35	0.82	NS/NI	100
13	96.26	30.41	0.72	NS/NI	89.90
14	96.50	16.70	0.01	NS/NI	82.31
15	95.89	38.05	0.60	NS/NI	80.51
16	95.87	40.30	0.10	NS/NI	78.83
17	96.50	21.34	0.01	NS/NI	83.07
18	95.83	37.64	0.60	NS/NI	51.66
19	96.46	29.18	0.15	NS/NI	91.28
20	96.16	30.91	0.14	NS/NI	78.08
21	95.23	9.96	0.01	NS/NI	66.81
22	95.88	38.05	0.07	NS/NI	60.53
23	96.59	33.57	0.27	NS/NI	86.02
24	96.59	33.64	0.50	NS/NI	88.18

a NS = non-substrate; NI = non-inhibitor; S = substrate; I = inhibitor.

Table 5, illustrates the metabolism and toxicity profiles of all synthesized compounds. Both CYP2D6 and CYP3A4 enzymes have a crucial role in the metabolism of drugs in the liver. In this regard, compounds 1–10 and 21–24 were neither substrates nor inhibitors of CYP2D6 while compounds 11–20 were both substrates and inhibitors of CYP2D6. In the case of CYP3A4 enzymes, none of the compounds were substrates/inhibitors of CYP3A4, except for 14, 16, and 22. The Ames test results represented that compounds 2–3, 6–7, 9–10, 12–13, 19–20, and 23–24 had negative values which indicated their non-mutagenic behavior. All the tested compounds were determined to be non-carcinogenic and, showed lower oral toxicity, so could be considered safe through oral administration. Most of the compounds have weak hERG inhibition, which means that they could not affect QT interval prolongation and cause violent cardiac side effects. Taken together, these results indicate that these synthesized compounds had proper physicochemical and pharmacokinetic properties which would introduce them as ideal candidates for further biological evaluations.

**Table 5:** Metabolism and toxicity profile of all α-benzyl amino coumarin compounds^a^

Entry	CYP2D6	CYP3A4	AMES toxicity	Carcinogenicity	hERG inhibition
1	NS/NI	NS/NI	Toxic	NC	Weak
2	NS/NI	NS/NI	Non toxic	NC	Strong
3	NS/NI	NS/NI	Non toxic	NC	Strong
4	NS/NI	NS/NI	Toxic	NC	Weak
5	NS/NI	NS/NI	Toxic	NC	Weak
6	NS/NI	NS/NI	Non toxic	NC	Weak
7	NS/NI	NS/NI	Non toxic	NC	Weak
8	NS/NI	NS/NI	Toxic	NC	Strong
9	NS/NI	NS/NI	Non toxic	NC	Strong
10	NS/NI	NS/NI	Non toxic	NC	Weak
11	S/I	NS/NI	Toxic	NC	Weak
12	S/I	NS/NI	Non toxic	NC	Weak
13	S/I	NS/NI	Non toxic	NC	Weak
14	S/I	S/I	Toxic	NC	Weak
15	S/I	NS/NI	Toxic	NC	Weak
16	S/I	S/NI	Toxic	NC	Weak
17	S/I	NS/NI	Toxic	NC	Weak
18	S/I	NS/NI	Toxic	NC	Weak
19	S/I	NS/NI	Non toxic	NC	Weak
20	S/I	NS/NI	Non toxic	NC	Weak
21	NS/NI	NS/NI	Toxic	NC	Weak
22	NS/NI	S/I	Toxic	NC	Weak
23	NS/NI	NS/NI	Non toxic	NC	Weak
24	NSN/I	NS/NI	Non toxic	NC	Weak

a hERG = Human ether-a-go-go related gene, NS = non-substrate; NI = non-inhibitor; S = substrate; I = inhibitor; NC = non-carcinogenic; C = carcinogenic.

## 3. Experimental

### 3.1. Reagents and instrumentation

All reagents were purchased from Merck and Aldrich and used without further purification. All yields refer to isolated products after purification. The NMR spectra were recorded on a Bruker Advance DPX 400 MHz instrument. The spectra were measured in DMSO-d6 relative to TMS (0.00 ppm). Elemental analysis was performed on a Heraeus CHN-O-Rapid analyser. TLC was performed on silica gel PolyGram SIL G/UV 254 plates. The powder X-ray diffraction patterns were measured with D8, Advance, Bruker, AXS, and diffractometer using CuKα irradiation. FE-SEM was taken by a Hitachi S-4160 photograph to examine the shape and size of BaSiO_3_ nanoparticles.

### 3.2. Preparation of barium silicate nanopowder

10 ml BaCl_2_ and 10 ml tetra ethyl orthosilicate were dissolved in 150 ml ethanol. Then ammonium 18% was added dropwise, while vigorously stirring at room temperature for 24 h. The resultant mixture was aged for 10 h. The gel was dried at 100 °C for 3 h and calcination at 1000 °C for 3 h. The resulting barium silicate was pulverized.^25^

### 3.3. General procedure

To a mixture of aldehyde (1.2 mmol), piperidine or other aliphatic amines (1.2 mmol) and 4-hydroxycoumarin (1 mmol) in water (5 ml), BaSiO_3_ nanopowder (0.26 mmol) as a catalyst was added and the mixture was stirred for a period of time at room temperature (Table 2). Progress of the reaction was monitored by TLC using *n*-hexane/ethyl acetate (9 : 1). Upon completion, the solvent was evaporated and the reaction mixture was diluted in hot ethanol. The catalyst was isolated by simple filtration, and the crude product was recrystallized in ethanol/water (75 : 25) to afford the pure product.

### 3.4. Spectral data

#### 3.4.1. 4-Hydroxy-3-(phenyl(piperidin-1-yl) methyl)-2*H*-chromen-2-one (A1)

Yield: 85% (0.285 mg), white solid, m.p.: 183–185 °C; ^1^H-NMR (400 MHz, DMSO-d_6_): *δ* = 1.81–1.91 (m, 6H), 2.24–2.31 (m, 2H), 3.05–3.12 (m, 2H), 5.36 (s, 1H), 7.19 (t, *J* = 7.6 Hz, 2H), 7.27 (t, *J* = 7.6 Hz, 1H), 7.34 (d, *J* = 7.6 Hz, 2H), 7.43 (t, *J* = 8.2 Hz, 1H), 7.52 (d, *J* = 8.2 Hz, 1H), 7.80 (t, *J* = 8.2 Hz, 1H), 7.89 (d, *J* = 8.4 Hz, 1H), 8.00–8.11 (brs, 1H, OH) ppm; ^13^C-NMR (100 MHz, DMSO-d_6_): *δ* = 22.1, 24.8, 49.6, 71.6, 103.3, 115.3, 119.5, 122.6, 125.3, 127.4, 128.7, 129.7, 130.3, 135.2, 154.5, 162.9, 174.2 ppm; elemental analysis for C_21_H_21_NO_3_: found: C, 75.12; H, 6.24; N, 4.14%; calculated: C, 75.20; H, 6.31; N, 4.18%.

#### 3.4.2. 3-((2-Chlorophenyl)(piperidin-1-yl)methyl)-4-hydroxy-2*H*-chromen-2-one (A2)

Yield: 87% (0.322 mg), white solid, m.p.: 186–188 °C; ^1^H-NMR (400 MHz, DMSO-d_6_): *δ* = 1.80–1.92 (m, 6H), 2.24–2.31 (m, 2H), 3.04–3.12 (m, 2H), 5.48 (s, 1H), 7.22 (t, *J* = 7.6 Hz, 1H), 7.28 (t, *J* = 7.8 Hz, 1H), 7.37 (d, *J* = 7.8 Hz, 1H), 7.44 (t, *J* = 8.0 Hz, 1H), 7.53 (d, *J* = 8.0 Hz, 1H), 7.64 (d, *J* = 7.7 Hz, 1H), 7.79–7.85 (m, 2H), 7.90 (d, *J* = 8.4 Hz, 1H) ppm; ^13^C-NMR (100 MHz, DMSO-d_6_): *δ* = 22.4, 25.0, 50.41, 74.7, 105.0, 116.1, 120.3, 125.0, 128.6, 129.1, 129.6, 130.1, 130.7, 131.3, 136.1, 144.2, 155.2, 164.4, 174.6 ppm; elemental analysis for C_21_H_20_ClNO_3_: found: C, 68.26; H, 5.52; N, 3.73%; calculated: C, 68.20; H, 5.45; N, 3.79%.

#### 3.4.3. 3-((4-Chlorophenyl)(piperidin-1-yl)methyl)-4-hydroxy-2*H*-chromen-2-one (A3)

Yield: 70% (0.259 mg), white solid, m.p.: 189–191 °C; ^1^H-NMR (400 MHz, DMSO-d_6_): *δ* = 1.82–1.91 (m, 6H), 2.21–2.30 (m, 2H), 3.06–3.12 (m, 2H), 5.48 (s, 1H), 7.24 (d, *J* = 7.7 Hz, 2H), 7.42–7.54 (m, 5H), 7.81 (t, *J* = 8.2 Hz, 1H), 7.90 (d, *J* = 8.4 Hz, 1H), ppm; ^13^C-NMR (100 MHz, DMSO-d_6_): *δ* = 21.4, 25.1, 49.7, 73.5, 104.5, 115.4, 120.7, 124.2, 129.3, 130.1, 130.7, 132.8, 135.2, 145.1, 154.7, 164.5, 174.3 ppm; elemental analysis for C_21_H_20_ClNO_3_: found: C, 68.14; H, 5.53; N, 3.87%; calculated: C, 68.20; H, 5.45; N, 3.79%.

#### 3.4.4. 4-Hydroxy-3-(piperidin-1-yl(*o*-tolyl) methyl)-2*H*-chromen-2-one (A4)

Yield: 84% (0.293 mg), white solid, m.p.: 201–203 °C; ^1^H-NMR (400 MHz, DMSO-d_6_): *δ* = 1.82–1.93 (m, 6H), 2.24–2.30 (m, 2H), 3.07–3.12 (m, 2H), 5.31 (s, 1H), 7.06 (d, *J* = 7.8 Hz, 1H), 7.12 (t, *J* = 7.8 Hz, 1H), 7.25–7.30 (m, 2H), 7.42 (t, *J* = 8.2 Hz, 1H), 7.52 (d, *J* = 8.0 Hz, 1H), 7.79 (t, *J* = 8.1 Hz, 1H), 7.88 (d, *J* = 8.2 Hz, 1H), 8.10–8.40 (m, 1H, OH) ppm; ^13^C-NMR (100 MHz, DMSO-d_6_): *δ* = 21.1, 21.7, 25.4, 49.6, 73.7, 103.8, 115.1, 120.2, 123.9, 124.5, 126.8, 127.4, 127.8, 130.4, 131.0, 134.0, 137.2, 154.8, 163.9, 173.7 ppm; elemental analysis for C_22_H_23_NO_3_: found: C, 75.54; H, 6.58; N, 4.11%; calculated: C, 75.62; H, 6.63; N, 4.01%.

#### 3.4.5. 4-Hydroxy-3-(piperidin-1-yl(*p*-tolyl) methyl)-2*H*-chromen-2-one (A5)

Yield: 81% (0.282 mg), white solid, m.p.: 197–199 °C; ^1^H-NMR (400 MHz, DMSO-d_6_): *δ* = 1.78–1.89 (m, 6H), 2.20–2.27 (m, 2H), 2.31 (s, 3H), 3.06–3.13 (m, 2H), 5.14 (s, 1H), 7.24 (d, *J* = 7.7 Hz, 2H), 7.42–7.54 (m, 5H), 7.81 (t, *J* = 8.2 Hz, 1H), 7.90 (d, *J* = 8.4 Hz, 1H), 7.13 (d, *J* = 7.7 Hz, 2H), 7.24 (d, *J* = 7.7 Hz, 2H), 7.42 (t, *J* = 8.4 Hz, 1H), 7.51 (d, *J* = 8.4 Hz, 1H), 7.79 (t, *J* = 8.2 Hz, 1H), 7.88 (d, *J* = 8.2 Hz, 1H) ppm; ^13^C-NMR (100 MHz, DMSO-d_6_): *δ* = 21.5, 22.4, 25.6, 49.9, 73.5, 103.9, 115.4, 120.1, 121.8, 124.5, 127.8, 130.4, 131.0, 134.0, 137.7, 154.7, 163.6, 173.9 ppm; elemental analysis for C_22_H_23_NO_3_: found: C, 75.66; H, 6.72; N, 4.08%; calculated: C, 75.62; H, 6.63; N, 4.01%.

#### 3.4.6. 4-Hydroxy-3-((4-methoxyphenyl)(piperidin-1-yl)methyl)-2*H*-chromen-2-one (A6)

Yield: 70% (0.256 mg), white solid, m.p.: 192–194 °C; ^1^H-NMR (400 MHz, DMSO-d_6_): *δ* = 1.79–1.86 (m, 6H), 2.22–2.27 (m, 2H), 3.08–3.11 (m, 2H), 3.79 (s, 3H), 4.09 (s, 1H), 6.96 (d, *J* = 7.7 Hz, 2H), 7.12 (d, *J* = 7.7 Hz, 2H), 7.42 (t, *J* = 8.4 Hz, 1H), 7.51 (d, *J* = 8.4 Hz, 1H), 7.79 (t, *J* = 8.2 Hz, 1H), 7.88 (d, *J* = 8.2 Hz, 1H) ppm; ^13^C-NMR (100 MHz, DMSO-d_6_): *δ* = 21.6, 25.5, 50.1, 56.1, 73.4, 103.7, 115.6, 117.2, 121.6, 124.1, 124.2, 129.1, 130.1, 131.0, 154.6, 156.2, 163.4, 173.1 ppm; elemental analysis for C_22_H_23_NO_4_: found: C, C, 72.37; H, 6.39; N, 3.89%; calculated: C, 72.31; H, 6.34; N, 3.83%.

#### 3.4.7. 3-((4-(*tert*-Butyl)phenyl)(piperidin-1-yl)methyl)-4-hydroxy-2*H*-chromen-2-one (A7)

Yield: 89% (0.348 mg), white solid, m.p.: 207–209 °C; ^1^H-NMR (400 MHz, DMSO-d_6_): *δ* = 1.29 (s, 9H), 1.82–1.89 (m, 6H), 2.23–2.26 (m, 2H), 3.04–3.09 (m, 2H), 5.29 (s, 1H), 7.14 (d, *J* = 7.7 Hz, 2H), 7.25 (d, *J* = 7.7 Hz, 2H), 7.43 (t, *J* = 8.4 Hz, 1H), 7.52 (d, *J* = 8.4 Hz, 1H), 7.80 (t, *J* = 8.2 Hz, 1H), 7.89 (d, *J* = 8.2 Hz, 1H), ppm; ^13^C-NMR (100 MHz, DMSO-d_6_): *δ* = 14.8, 21.4, 25.5, 34.3, 50.3, 73.6, 103.4, 115.4, 121.6, 124.7, 126.7, 127.9, 130.2, 131.2, 133.5, 138.2, 154.5, 163.1, 173.5 ppm; elemental analysis for C_25_H_29_NO_3_: found: C, 76.77; H, 7.53; N, 3.51%; calculated: C, 76.70; H, 7.47; N, 3.58%.

#### 3.4.8. 4-Hydroxy-3-((3-nitrophenyl)(piperidin-1-yl) methyl)-2*H*-chromen-2-one (A_8_)

Yield: 90% (0.342 mg), yellow solid, m.p.: 218–220 °C; ^1^H-NMR (400 MHz, DMSO-d_6_): *δ* = 1.83–1.92 (m, 6H), 2.25–2.29 (m, 2H), 3.08–3.13 (m, 2H), 5.72 (s, 1H), 7.44 (t, *J* = 8.0 Hz, 1H), 7.51 (d, *J* = 8.0 Hz, 1H), 7.56 (t, *J* = 7.8 Hz, 1H), 7.80 (t, *J* = 8.0 Hz, 1H), 7.91 (d, *J* = 8.1 Hz, 1H), 8.27 (d, *J* = 7.8 Hz, 1H), 8.02 (d, *J* = 7.8 Hz, 1H), 8.49 (s, 1H), 8.50–8.90 (m, 1H, OH) ppm; ^13^C-NMR (100 MHz, DMSO-d_6_): *δ* = 21.9, 25.7, 50.5, 75.6, 103.7, 115.9, 121.7, 124.8, 128.7, 129.1, 129.6, 130.1, 130.9, 131.4, 134.5, 139.2, 154.7, 164.1, 175.5 ppm; elemental analysis for C_21_H_20_N_2_O_5_: found: C, 66.26; H, 5.25; N, 7.31%; calculated: C, 66.31; H, 5.30; N, 7.36%.

#### 3.4.9. 3-((2,4-Dichlorophenyl)(piperidin-1-yl)methyl)-4-hydroxy-2*H*-chromen-2-one (A_9_)

Yield: 61% (0.246 mg), white solid, m.p.: 211–213 °C; ^1^H-NMR (400 MHz, DMSO-d_6_): *δ* = 1.84–1.90 (m, 6H), 2.25–2.28 (m, 2H), 3.08–3.12 (m, 2H), 5.52 (s, 1H), 7.41–7.51 (m, 3H), 7.69 (d, *J* = 7.8 Hz, 1H), 7.77–7.81 (m, 2H), 7.88 (d, *J* = 8.3 Hz, 1H) 7.95–8.30 (m, 1H, OH) ppm; ^13^C-NMR (100 MHz, DMSO-d_6_): *δ* = 21.7, 25.8, 50.3, 74.3, 103.5, 115.7, 122.0, 124.6, 127.7, 128.9, 129.3, 130.2, 131.4, 134.1, 141.2, 143.6, 154.6, 163.9, 174.5 ppm; elemental analysis for C_21_H_19_Cl_2_NO_3_: found: C, 62.39; H, 4.74; N, 3.46%; calculated: C, 62.39; H, 4.74; N, 3.46%.

#### 3.4.10. 3-((4-Bromophenyl)(piperidin-1-yl)methyl)-4-hydroxy-2*H*-chromen-2-one (A_10_)

Yield: 90% (0.373 mg), white solid, m.p.: 203–205 °C; ^1^H-NMR (400 MHz, DMSO-d_6_): *δ* = 1.78–1.88 (m, 6H), 2.22–2.28 (m, 2H), 3.08–3.14 (m, 2H), 5.59 (s, 1H), 7.39–7.52 (m, 5H), 7.75–7.89 (m, 5H) ppm; ^13^C-NMR (100 MHz, DMSO-d_6_): *δ* = 21.7, 25.4, 49.8, 73.9, 103.5, 115.1, 121.6, 124.3, 128.9, 130.2, 131.3, 132.9, 133.7, 148.3, 154.7, 163.9, 174.7 ppm; elemental analysis for C_21_H_20_BrNO_3_: found: C, 60.85; H, 4.82; N, 3.31%; calculated: C, 60.88; H, 4.87; N, 3.38%.

#### 3.4.11. 4-Hydroxy-3-(phenyl(pyrrolidin-1-yl) methyl)-2*H*-chromen-2-one (A11)

Yield: 82% (0.264 mg), white solid, m.p.: 167–169 °C; ^1^H-NMR (400 MHz, DMSO-d_6_): *δ* = 2.02–2.10 (m, 4H), 3.02–3.08 (m, 2H), 3.41–3.47 (m, 2H), 5.19 (s, 1H), 7.19–7.33 (m, 5H), 7.41 (t, *J* = 8.4 Hz, 1H), 7.49 (d, *J* = 8.2 Hz, 1H), 7.69 (t, *J* = 8.3 Hz, 1H), 7.93 (d, *J* = 8.2 Hz, 1H), 7.99 (brs, 1H, OH) ppm; ^13^C-NMR (100 MHz, DMSO-d_6_): *δ* = 26.7, 53.6, 72.3, 103.5, 115.7, 120.1, 123.6, 125.7, 127.8, 128.6, 129.5, 130.3, 134.9, 154.7, 163.1, 173.9 ppm; elemental analysis for C_20_H_19_NO_3_: found: C, 74.69; H, 5.99; N, 4.42; calculated: C, 74.75; H, 5.96; N, 4.36%.

#### 3.4.12. 3-((4-Bromophenyl) (pyrrolidin-1-yl)methyl)-4-hydroxy-2*H*-chromen-2-one (A12)

Yield: 90% (0.360 mg), white solid, m.p.: 188–190 °C; ^1^H-NMR (400 MHz, DMSO-d_6_): *δ* = 2.04–2.11 (m, 4H), 3.01–3.06 (m, 2H), 3.41–3.43 (m, 2H), 5.39 (s, 1H), 7.35 (d, *J* = 7.7 Hz, 2H), 7.40 (t, *J* = 8.4 Hz, 1H), 7.48 (d, *J* = 8.2 Hz, 1H), 7.65 (d, *J* = 7.7 Hz, 2H), 7.67 (t, *J* = 8.3 Hz, 1H), 7.90 (d, *J* = 8.2 Hz, 1H), 7.96 (brs, 1H, OH) ppm; ^13^C-NMR (100 MHz, DMSO-d_6_): *δ* = 26.4, 53.6, 74.2, 103.7, 115.1, 121.6, 124.5, 128.8, 130.2, 131.2, 133.3, 134.6, 148.1, 154.7, 164.1, 174.8 ppm; elemental analysis for C_20_H_18_BrNO_3_: found: C, 60.08; H, 4.59; N, 3.55; calculated: C, 60.01; H, 4.53; N, 3.50%.

#### 3.4.13. 3-((4-Chlorophenyl) (pyrrolidin-1-yl)methyl)-4-hydroxy-2*H*-chromen-2-one (A13)

Yield: 93% (0.331 mg), white solid, m.p.: 175–177 °C; ^1^H-NMR (400 MHz, DMSO-d_6_): *δ* = 2.04–2.12 (m, 4H), 3.00–3.06 (m, 2H), 3.40–3.46 (m, 2H), 5.46 (s, 1H), 7.27 (d, *J* = 7.7 Hz, 2H), 7.41 (t, *J* = 8.4 Hz, 1H), 7.48 (d, *J* = 7.7 Hz, 2H), 7.52 (d, *J* = 8.4 Hz, 1H), 7.67 (brs, 1H, OH), 7.80 (t, *J* = 8.2 Hz, 1H), 7.90 (d, *J* = 8.2 Hz, 1H) ppm; ^13^C-NMR (100 MHz, DMSO-d_6_): *δ* = 26.4, 52.8, 74.1, 105.9, 115.8, 121.2, 124.6, 129.8, 130.1, 131.3, 132.8, 135.1, 144.5, 154.7, 164.1, 174.8 ppm; elemental analysis for C_20_H_18_ClNO_3_: found: C, 67.44; H, 5.02; N, 3.85; calculated: C, 67.51; H, 5.10; N, 3.94%.

#### 3.4.14. 4-Hydroxy-3-((4-nitrophenyl) (pyrrolidin-1-yl) methyl)-2*H*-chromen-2-one (A14)

Yield: 88% (0.322 mg), yellow solid, m.p.: 193–195 °C; ^1^H-NMR (400 MHz, DMSO-d_6_): *δ* = 2.06–2.12 (m, 4H), 3.04–3.07 (m, 2H), 3.44–3.47 (m, 2H), 5.76 (s, 1H), 7.42 (t, *J* = 8.3 Hz, 1H), 7.52 (d, *J* = 8.3 Hz, 1H), 7.77 (d, *J* = 7.9 Hz, 2H), 7.81 (t, *J* = 8.3 Hz, 1H), 7.91 (d, *J* = 8.3 Hz, 1H), 7.90–8.20 (brs, 1H, OH), 8.28 (d, *J* = 7.8 Hz, 2H) ppm; ^13^C-NMR (100 MHz, DMSO-d_6_): *δ* = 26.4, 53.4, 77.4, 106.1, 116.0, 121.2, 124.6, 130.2, 130.6, 131.4, 134.1, 137.1, 139.5, 154.8, 164.6, 175.6 ppm; elemental analysis for C_20_H_18_N_2_O_5_: found: C, 65.66; H, 4.99; N, 7.61; calculated: C, 65.57; H, 4.95; N, 7.65%.

#### 3.4.15. 4-Hydroxy-3-(pyrrolidin-1-yl(*p*-tolyl)methyl)-2*H*-chromen-2-one (A15)

Yield: 89% (0.299 mg), white solid, m.p.: 176–178 °C; ^1^H-NMR (400 MHz, DMSO-d_6_): *δ* = 2.01–2.09 (m, 4H), 2.28 (s, 3H), 3.01–3.05 (m, 2H), 3.41–3.45 (m, 2H), 5.16 (s, 1H), 7.01 (d, *J* = 7.7 Hz, 2H), 7.21 (d, *J* = 7.7 Hz, 2H), 7.40 (t, *J* = 8.4 Hz, 1H), 7.48 (d, *J* = 8.2 Hz, 1H), 7.69 (t, *J* = 8.3 Hz, 1H), 7.89–7.94 (m, 3H) ppm; ^13^C-NMR (100 MHz, DMSO-d_6_): *δ* = 21.6, 26.1, 51.2, 73.1, 104.8, 115.2, 120.1, 121.6, 124.7, 128.2, 130.2, 131.3, 134.1, 137.8, 154.5, 163.3, 173.2 ppm; elemental analysis for C_21_H_21_NO_3_: found: C, 75.14; H, 6.27; N, 4.13; calculated: C, 75.20; H, 6.31; N, 4.18%.

#### 3.4.16. 4-Hydroxy-3-((4-methoxyphenyl) (pyrrolidin-1-yl) methyl)-2*H*-chromen-2-one (A16)

Yield: 95% (0.334 mg), white solid, m.p.: 174–176 °C; ^1^H-NMR (400 MHz, DMSO-d_6_): *δ* = 2.02–2.09 (m, 4H), 3.01–3.05 (m, 2H), 3.41–3.45 (m, 2H), 3.73 (s, 3H), 5.16 (s, 1H), 6.91 (d, *J* = 7.7 Hz, 2H), 7.07 (d, *J* = 7.7 Hz, 2H), 7.40 (t, *J* = 8.4 Hz, 1H), 7.48 (d, *J* = 8.2 Hz, 1H), 7.67–7.71 (m, 2H), 7.92 (d, *J* = 8.2 Hz, 1H) ppm; ^13^C-NMR (100 MHz, DMSO-d_6_): *δ* = 26.5, 51.1, 56.7, 73.1, 103.5, 115.8, 117.5, 121.4, 124.3, 124.8, 129.0, 130.3, 131.2, 154.6, 156.4, 163.6, 171.4 ppm; elemental analysis for C_21_H_21_NO_4_: found: C, 71.74; H, 5.98; N, 3.95; calculated: C, 71.78; H, 6.02; N, 3.99%.

#### 3.4.17. 4-Hydroxy-3-((3-nitrophenyl)(pyrrolidin-1-yl)methyl)-2*H*-chromen-2-one (A17)

Yield: 92% (0.337 mg), yellow solid, m.p.: 195–197 °C; ^1^H-NMR (400 MHz, DMSO-d_6_): *δ* = 2.02–2.12 (m, 4H), 3.01–3.08 (m, 2H), 3.38–3.45 (m, 2H), 5.79 (s, 1H), 7.43 (t, *J* = 8.1 Hz, 1H), 7.52 (d, *J* = 8.1 Hz, 1H), 7.55 (t, *J* = 7.8 Hz, 1H), 7.78 (t, *J* = 8.2 Hz, 1H), 7.90 (d, *J* = 8.2 Hz, 1H), 7.98 (d, *J* = 7.8 Hz, 1H), 8.25 (d, *J* = 7.8 Hz, 1H), 8.47 (s, 1H), 9.40–9.70 (brs, 1H, OH) ppm; ^13^C-NMR (100 MHz, DMSO-d_6_): *δ* = 26.7, 51.8, 77.3, 104.1, 116.1, 121.5, 124.6, 128.9, 129.4, 129.9, 130.2, 131.2, 131.6, 135.3, 139.7, 154.6, 164.3, 176.4 ppm; elemental analysis for C_20_H_18_N_2_O_5_: found: C, 65.64; H, 5.03; N, 7.63; calculated: C, 65.57; H, 4.95; N, 7.65%.

#### 3.4.18. 4-Hydroxy-3-(morpholino(phenyl)methyl)-2*H*-chromen-2-one (A18)

Yield: 93% (0.314 mg), white solid, m.p.: 172–174 °C; ^1^H-NMR (400 MHz, DMSO-d_6_): *δ* = 2.95–3.01 (m, 2H), 3.29–3.35 (m, 2H), 3.73–3.92 (m, 4H), 5.34 (s, 1H), 7.19–7.33 (m, 6H), 7.45 (t, *J* = 8.2 Hz, 1H), 7.55 (d, *J* = 8.3 Hz, 1H), 7.82 (t, *J* = 8.2 Hz, 1H), 7.90 (d, *J* = 8.2 Hz, 1H) ppm; ^13^C-NMR (100 MHz, DMSO-d_6_): *δ* = 59.8, 64.6, 74.6, 104.4, 116.2, 120.7, 123.6, 125.7, 127.6, 128.7, 129.6130.4, 136.4, 155.2, 163.9, 174.6 ppm; elemental analysis for C_20_H_19_NO_4_: found: C, 71.26; H, 5.74; N, 4.08; calculated: C, 71.20; H, 5.68; N, 4.15%.

#### 3.4.19. 3-((4-Bromophenyl)(morpholino)methyl)-4-hydroxy-2*H*-chromen-2-one (A19)

Yield: 90% (0.375 mg), white solid, m.p.: 189–191 °C; ^1^H-NMR (400 MHz, DMSO-d_6_): *δ* = 2.96–3.02 (m, 2H), 3.31–3.95 (m, 6H), 5.44 (s, 1H), 7.32 (d, *J* = 7.7 Hz, 2H), 7.42–7.56 (m, 5H), 7.82 (t, *J* = 8.2 Hz, 1H), 7.91 (d, *J* = 8.2 Hz, 1H) ppm; ^13^C-NMR (100 MHz, DMSO-d_6_): *δ* = 60.4, 66.6, 78.2, 104.7, 116.1, 121.6, 124.4, 128.9, 130.2, 131.1, 133.4, 135.6, 148.4, 155.2, 164.3, 174.1 ppm; elemental analysis for C_20_H_18_BrNO_4_: found: C, 57.75; H, 4.44; N, 3.42; calculated: C, 57.71; H, 4.36; N, 3.36%.

#### 3.4.20. 3-((4-Chlorophenyl)(morpholino)methyl)-4-hydroxy-2*H*-chromen-2-one (A20)

Yield: 98% (0.364 mg), white solid, m.p.: 171–173 °C; ^1^H NMR (400 MHz, DMSO-d_6_): *δ* = 2.94–3.97 (m, 8H), 5.67 (s, 1H), 7.26 (d, *J* = 7.8 Hz, 2H), 7.41–7.53 (m, 4H), 7.78 (t, *J* = 8.3 Hz, 1H), 7.90 (d, *J* = 8.3 Hz, 1H), 7.90–8.00 (brs, 1H, OH) ppm; ^13^C-NMR (100 MHz, DMSO-d_6_): *δ* = 60.1, 66.4, 76.7, 104.5, 116.0, 121.6, 124.3, 127.8, 129.6, 130.1, 131.3, 134.8, 142.4, 155.3, 164.1, 172.1 ppm; elemental analysis for C_20_H_18_ClNO_4_: found: C, 64.53; H, 4.79; N, 3.81; calculated: C, 64.61; H, 4.88; N, 3.77%.

#### 3.4.21. 4-Hydroxy-3-(morpholino(4-nitrophenyl) methyl)-2*H*-chromen-2-one (A21)

Yield: 78% (0.298 mg), yellow solid, m.p.: 185–187 °C; ^1^H-NMR (400 MHz, DMSO-d_6_): *δ* = 2.96–3.94 (m, 8H), 6.39 (s, 1H), 7.42 (t, *J* = 8.4 Hz, 1H), 7.49 (d, *J* = 8.4 Hz, 1H), 7.67 (t, *J* = 8.4 Hz, 1H), 7.78 (d, *J* = 7.7 Hz, 2H), 7.94 (d, *J* = 8.4 Hz, 1H), 8.03 (d, *J* = 7.7 Hz, 2H), 7.90–8.00 (brs, 1H, OH) ppm; ^13^C-NMR (100 MHz, DMSO-d_6_): *δ* = 61.2, 68.3, 82.4, 106.3, 116.4, 121.3, 124.6, 129.3, 130.4, 131.3, 132.4, 137.3, 140.1, 155.2, 164.8, 178.9 ppm; elemental analysis for C_20_H_18_N_2_O_4_: found: C, 62.73; H, 4.71; N, 7.28%; calculated: C, 62.82; H, 4.75; N, 7.33%.

#### 3.4.22. 4-Hydroxy-3-(morpholino(*p*-tolyl) methyl)-2*H*-chromen-2-one (A22)

Yield: 90% (0.316 mg), white solid, m.p.: 173–175 °C; ^1^H-NMR (400 MHz, DMSO-d_6_): *δ* = 2.94–3.97 (m, 8H), 5.48 (s, 1H), 7.14 (d, *J* = 7.8 Hz, 2H), 7.26 (d, *J* = 7.8 Hz, 2H), 7.42 (t, *J* = 8.3 Hz, 1H), 7.51 (d, *J* = 8.3 Hz, 1H), 7.77 (t, *J* = 8.3 Hz, 1H), 7.89 (d, *J* = 8.3 Hz, 1H), 7.95–8.10 (brs, 1H, OH) ppm; ^13^C-NMR (100 MHz, DMSO-d_6_): *δ* = 21.5, 59.7, 65.8, 74.2, 104.9, 115.4, 120.2, 121.6, 124.7, 128.6, 130.1, 131.4, 134.0, 137.7, 154.5, 163.7, 174.2 ppm; elemental analysis for C_21_H_21_NO_4_: found: C, 71.71; H, 6.09; N, 4.06%; calculated: C, 71.78; H, 6.02; N, 3.99%.

#### 3.4.23. 3-((2,4-Dichlorophenyl)(morpholino)methyl)-4-hydroxy-2*H*-chromen-2-one (A23)

Yield: 92% (0.374 mg), white solid, m.p.: 193–195 °C; ^1^H-NMR (400 MHz, DMSO-d_6_): *δ* = 2.95–3.99 (m, 8H), 5.82 (s, 1H), 7.41–7.46 (m, 3H), 7.50 (d, *J* = 8.3 Hz, 1H), 7.69 (d, *J* = 7.8 Hz, 1H), 7.79 (t, *J* = 8.2 Hz, 1H), 7.81 (s, 1H), 7.88 (d, *J* = 8.3 Hz, 1H), 7.94–8.25 (brs, 1H, OH) ppm; ^13^C-NMR (100 MHz, DMSO-d_6_): *δ* = 60.3, 66.4, 76.9, 105.1, 115.6, 121.7, 124.7, 128.8, 129.2, 130.1, 131.3, 132.3, 135.4, 143.7, 145.6, 154.8, 164.1, 177.2 ppm; elemental analysis for C_20_H_17_Cl_2_NO_4_: found: C, 59.01; H, 4.15; N, 3.33%; calculated: C, 59.13; H, 4.22; N, 3.45%.

#### 3.4.24. 3-((3,5-Dichlorophenyl)(morpholino)methyl)-4-hydroxy-2*H*-chromen-2-one (A24)

Yield: 89% (0.362 mg), white solid, m.p.: 196–198 °C;^1^H-NMR (400 MHz, DMSO-d_6_): *δ* = 2.94–3.98 (m, 8H), 5.80 (s, 1H), 7.42 (t, *J* = 8.4 Hz, 1H), 7.49 (d, *J* = 8.4 Hz, 1H), 7.75–7.90 (m, 5H), 8.11 (s, 1H) ppm; ^13^C-NMR (100 MHz, DMSO-d_6_): *δ* = 60.3, 66.7, 75.7, 105.0, 115.7, 121.4, 124.3, 129.8, 130.1, 131.4, 133.3, 134.8, 142.7, 155.0, 164.3, 176.6 ppm; elemental analysis for C_20_H_17_Cl_2_NO_4_: found: C, 59.34; H, 4.28; N, 3.36%; calculated: C, 59.13; H, 4.22; N, 3.45%.

### 3.5. *In silico* physicochemical parameters and ADMET profiling calculations

The physicochemical properties and drug-likeness studies of all α-benzyl amino coumarin compounds were obtained and evaluated for ADMET characteristics under Lipinski's rule of five and Veber rules by using SwissADME online software, the preADMET online server (https://preadmet.webservice.bmdrc.org/) and admetSAR (http://lmmd.ecust.edu.cn/admetsar2/) online server.

## 4. Conclusion

In summary, we have demonstrated an elegant and environmentally friendly protocol for the synthesis of α-benzyl amino coumarin derivatives by using barium silicate nanoparticles as a catalyst and water as a green solvent. Compared with other methods in existence, our method provides the advantages of better yields, inexpensive operation, cleaner reaction profile, and environmental friendliness. The *in silico* ADMET analysis performed within this study indicated that all synthesized compounds are in agreement with Lipinski's and Veber's rules. Also, the results of toxicity risk assessment tests showed that none of the compounds exhibit a risk of mutagenicity, tumorigenicity, irritation, or acute oral toxicity which highlights their potential as ideal chemotherapeutic agents.

## Conflicts of interest

There are no conflicts to declare.

## Supplementary Material

RA-013-D3RA00796K-s001
