# Supplementary material for: Barium silicate nanoparticles, an efficient catalyst for one-pot green synthesis of α-benzyl amino coumarin derivatives as potential chemotherapeutic agents
Source: RSC Adv. 2023 Jul 12;13(31):21127–37. doi: 10.1039/d3ra00796k (PMC10337722; doi:10.1039/d3ra00796k)
Supplement: RA-013-D3RA00796K-s001 [file RA-013-D3RA00796K-s001.pdf]

Electronic Supplementary Material (ESI) for RSC Advances.
This journal is © The Royal Society of Chemistry 2023

Barium Silicate Nanoparticles, An Efficient Catalyst for One-Pot Green Synthesis of A-Benzyl Amino Coumarin Derivatives as Potential Chemotherapeutic Agents

Hadi Taghrir[1], Leila Emami[1], Zeinab Faghih[1*], Shadi Dalili[2], Soghra Khabnadideh[1], Majid Ghashang[3*]

[1]Pharmaceutical Sciences Research Center, Shiraz University of Medical Sciences, Shiraz, Islamic Republic of Iran

[2]Department of Physical and Environmental Sciences, 1265 Military Trail, Scarborough, ON M1C 1A4 Canada

[3]Department of Chemistry, Najafabad Branch, Islamic Azad University, Najafabad, Iran.

Zeinab Faghih: Tel: +98-7132424127, Fax: +98-7132424126; Faghihl@sums.ac.ir, and layafaghih@gmail.com

Majid Ghashang: Tel: +98-3142291004; Fax: +98-3142291016; E-mail: ghashangmajid@pmt.iaun.ac.ir,

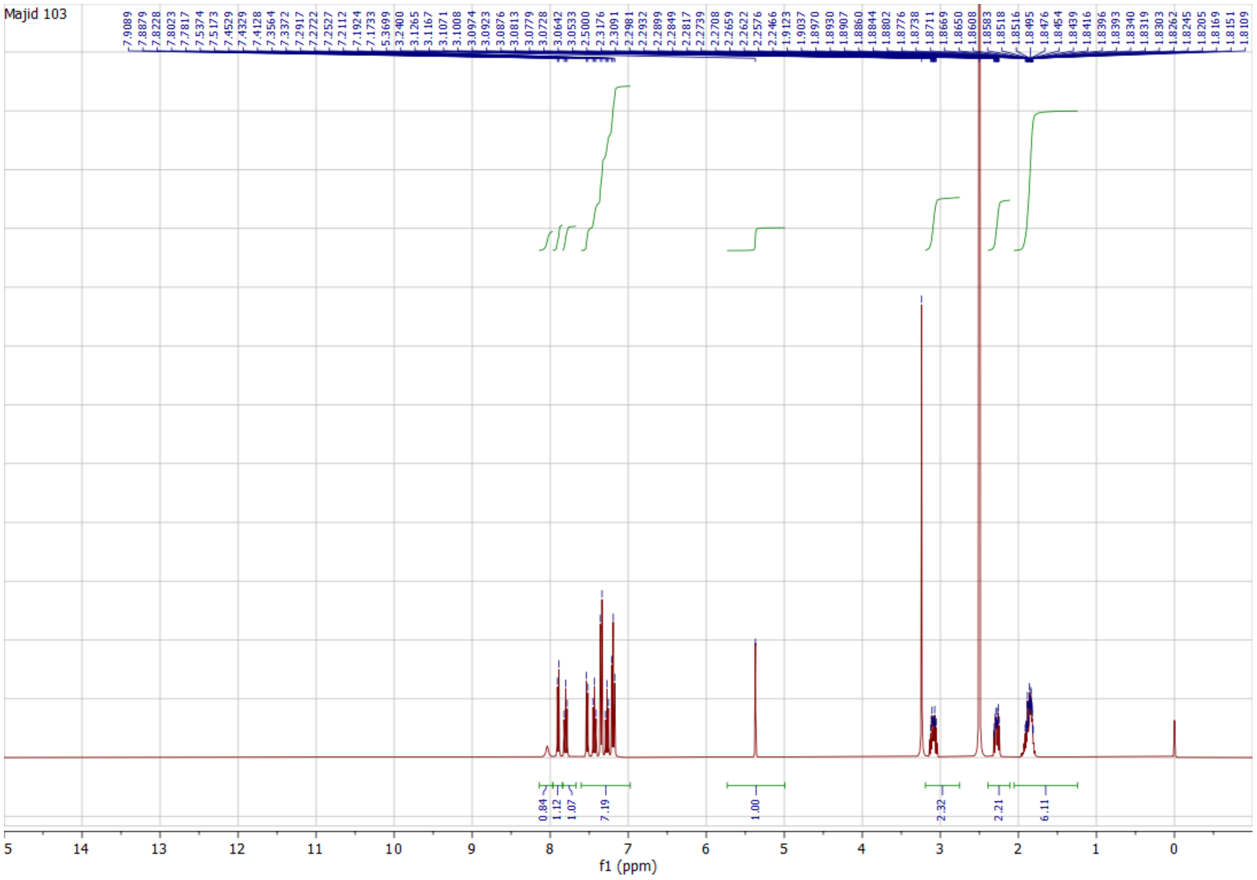

Figure S_1: ^{1}H-NMR spectra of 4-hydroxy-3-(phenyl(piperidin-1-yl) methyl)-2H-chromen-2-one

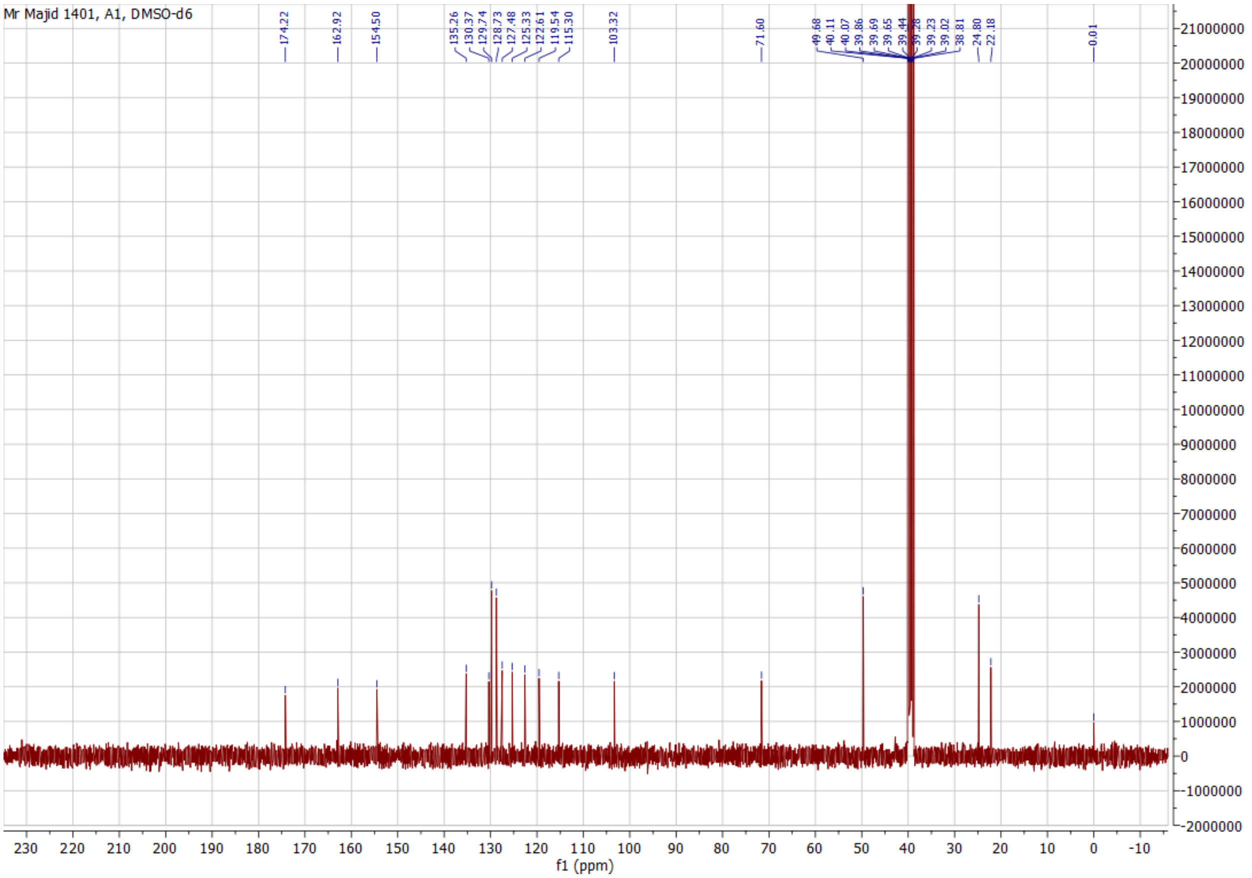

Figure S$_2$: ^{13}C-NMR spectra of 4-hydroxy-3-(phenyl(piperidin-1-yl) methyl)-2H-chromen-2-one

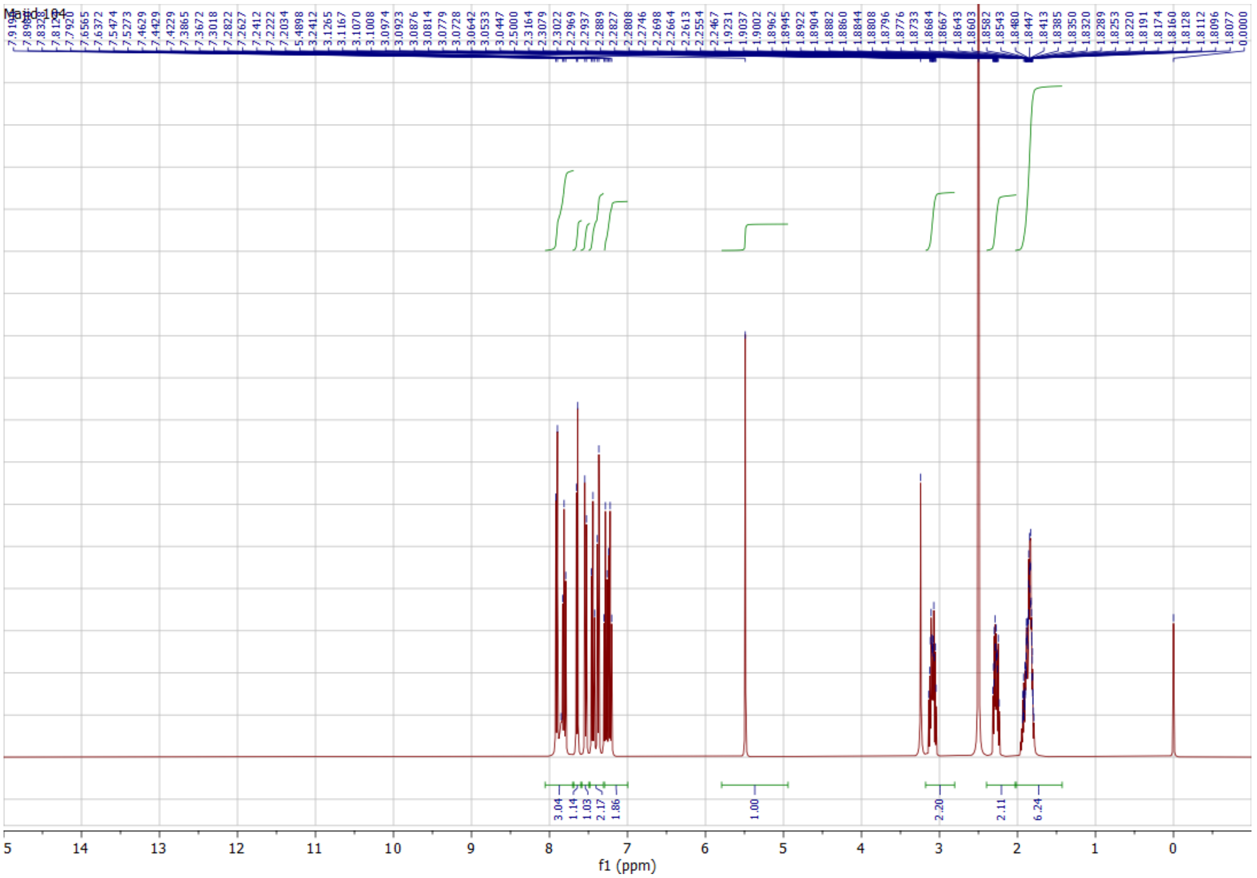

Figure S$_3$: ^{1}H-NMR spectra of 3-((2-chlorophenyl) (piperidin-1-yl)methyl)-4-hydroxy-2H-chromen-2-one

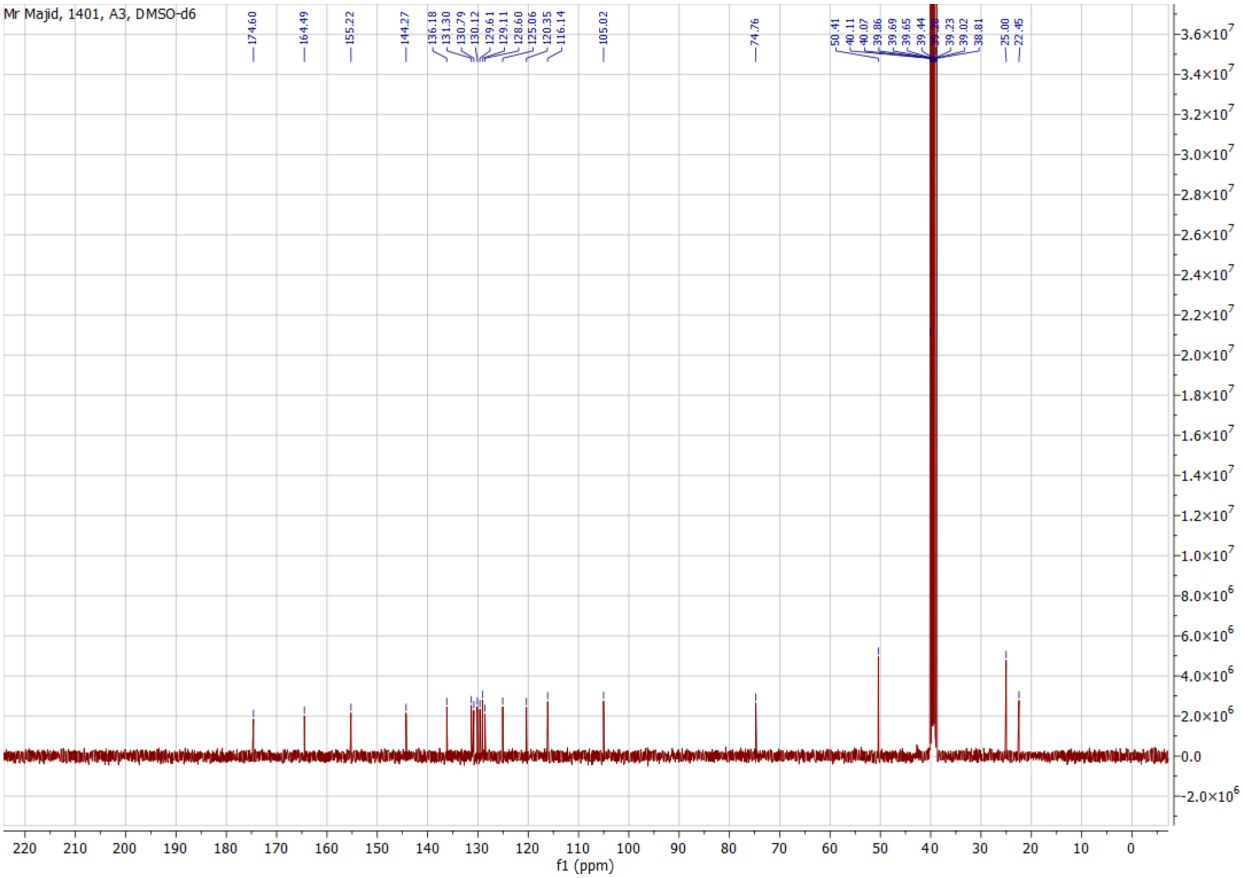

Figure S_4: ^{13}C-NMR spectra of 3-((2-chlorophenyl) (piperidin-1-yl) methyl)-4-hydroxy-2H-chromen-2-one

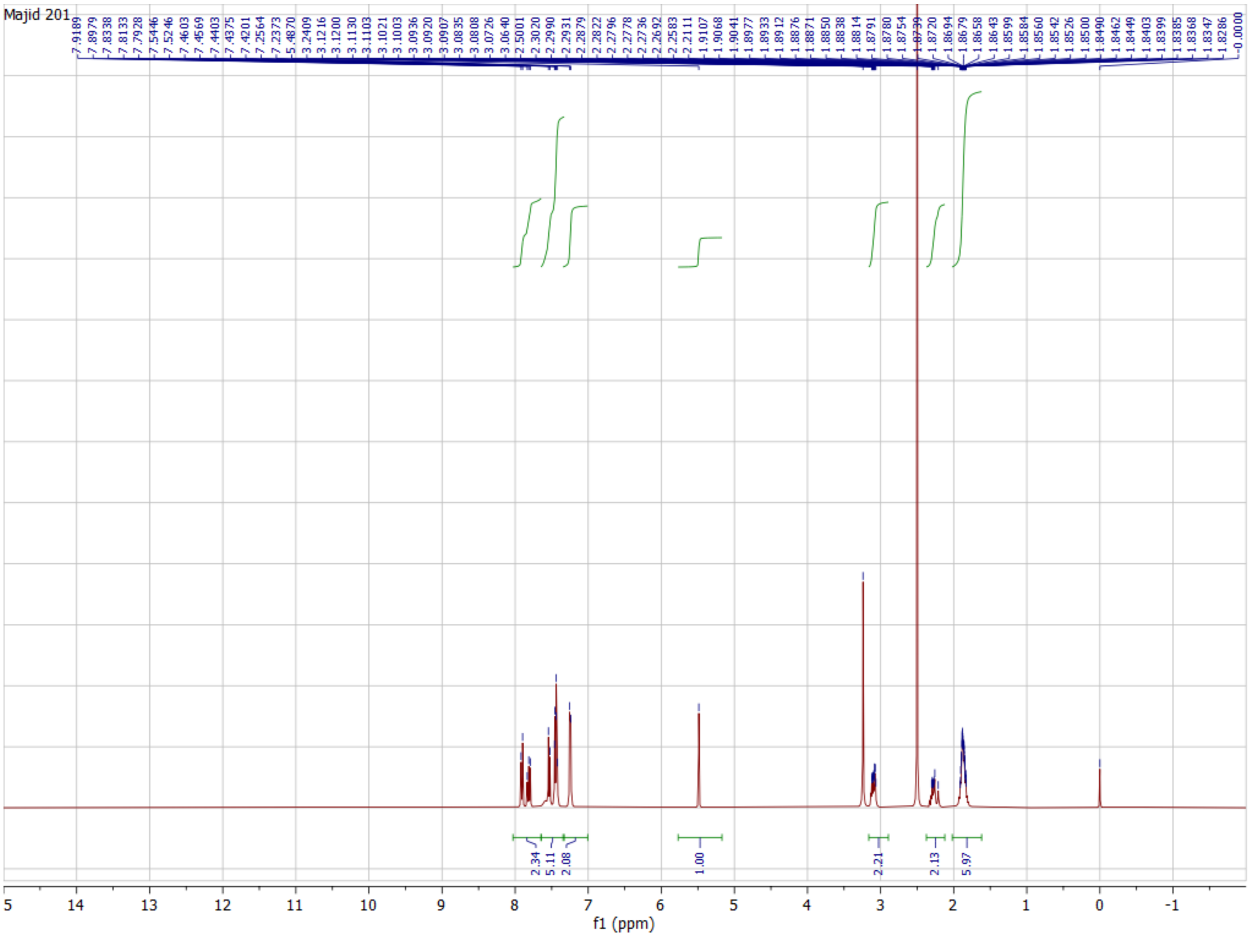

Figure S$_5$: ^{1}H-NMR spectra of 3-((4-chlorophenyl) (piperidin-1-yl) methyl)-4-hydroxy-2H-chromen-2-one

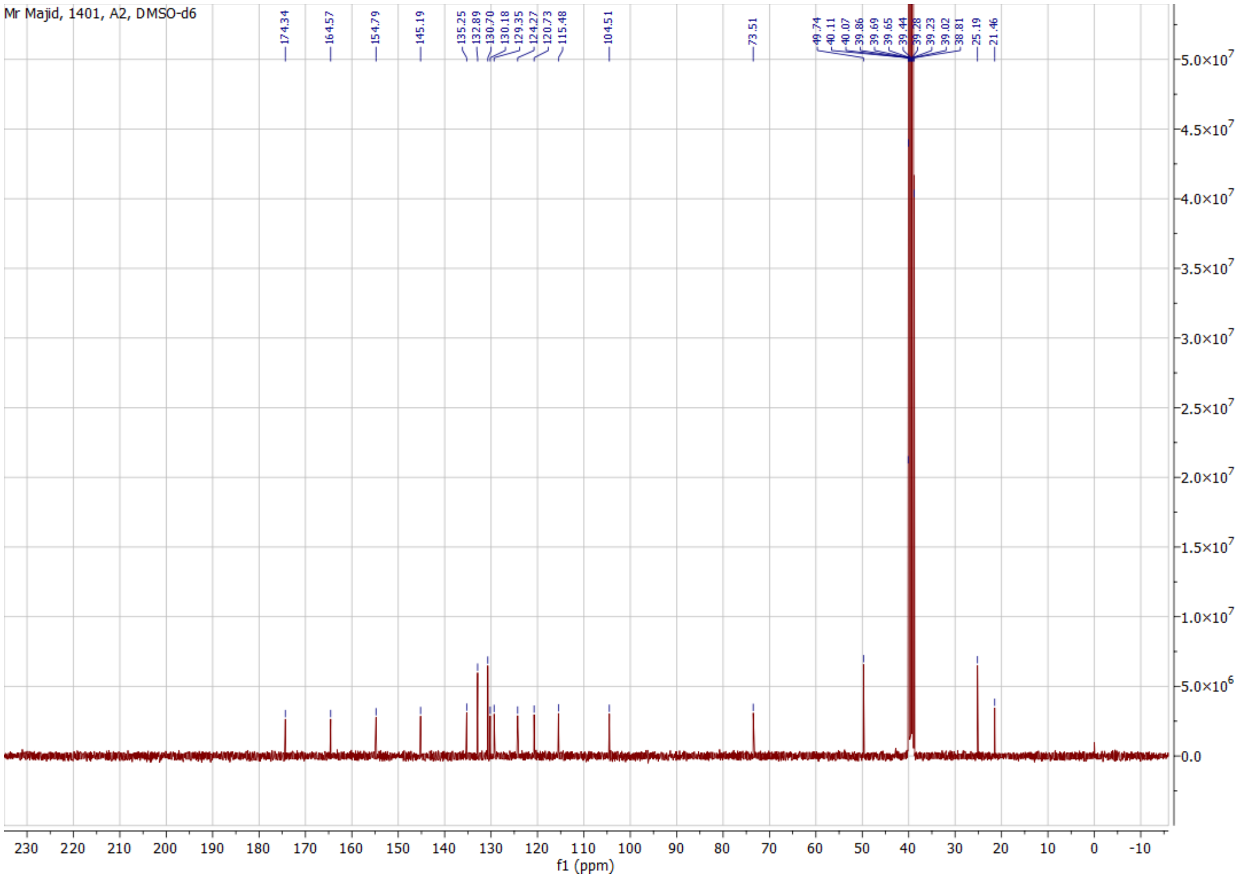

Figure S$_6$: ^{13}C-NMR spectra of 3-((4-chlorophenyl) (piperidin-1-yl) methyl)-4-hydroxy-2H-chromen-2-one

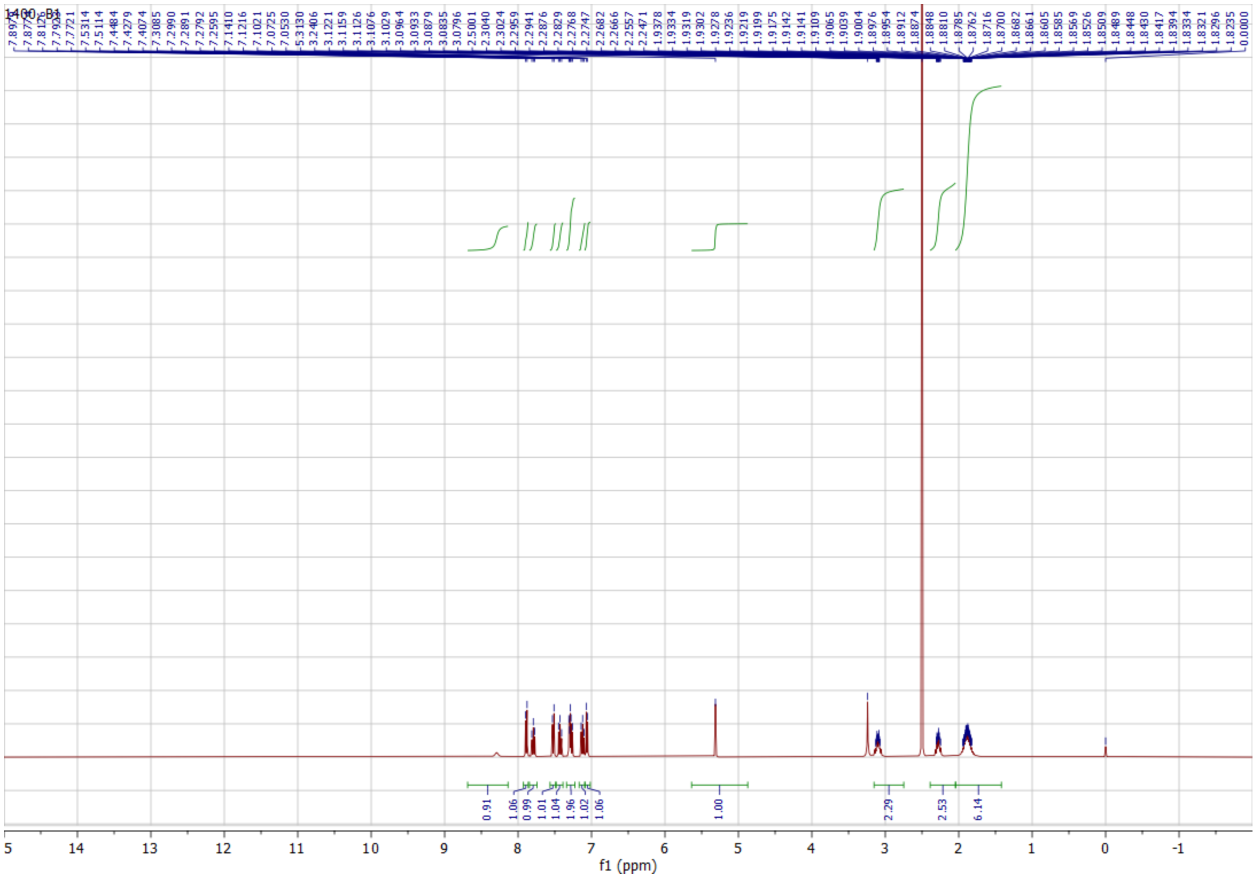

Figure S$_7$: ^{1}H-NMR spectra of 4-hydroxy-3-(piperidin-1-yl(o-tolyl) methyl)-2H-chromen-2-one

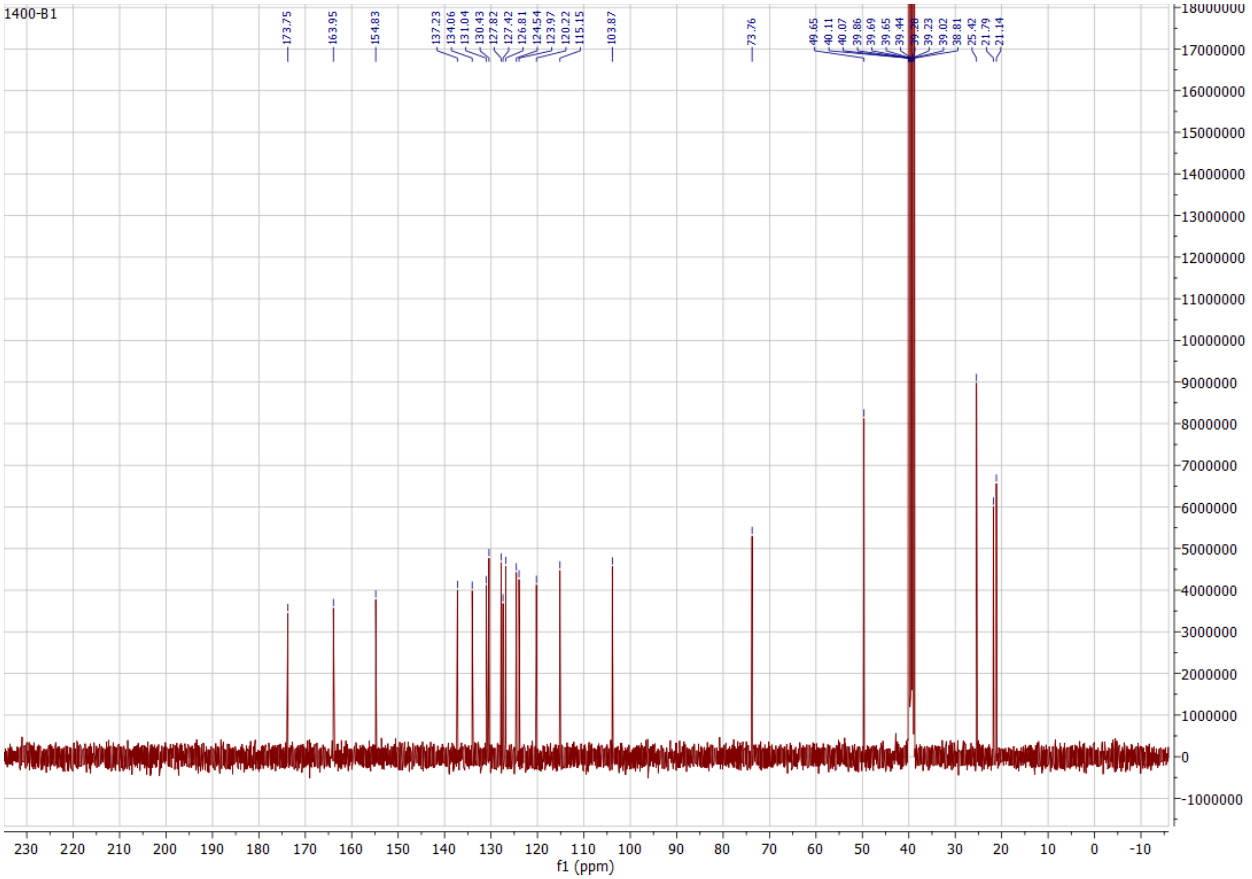

Figure S$_8$: ^{13}C-NMR spectra of 4-hydroxy-3-(piperidin-1-yl(o-tolyl) methyl)-2H-chromen-2-one

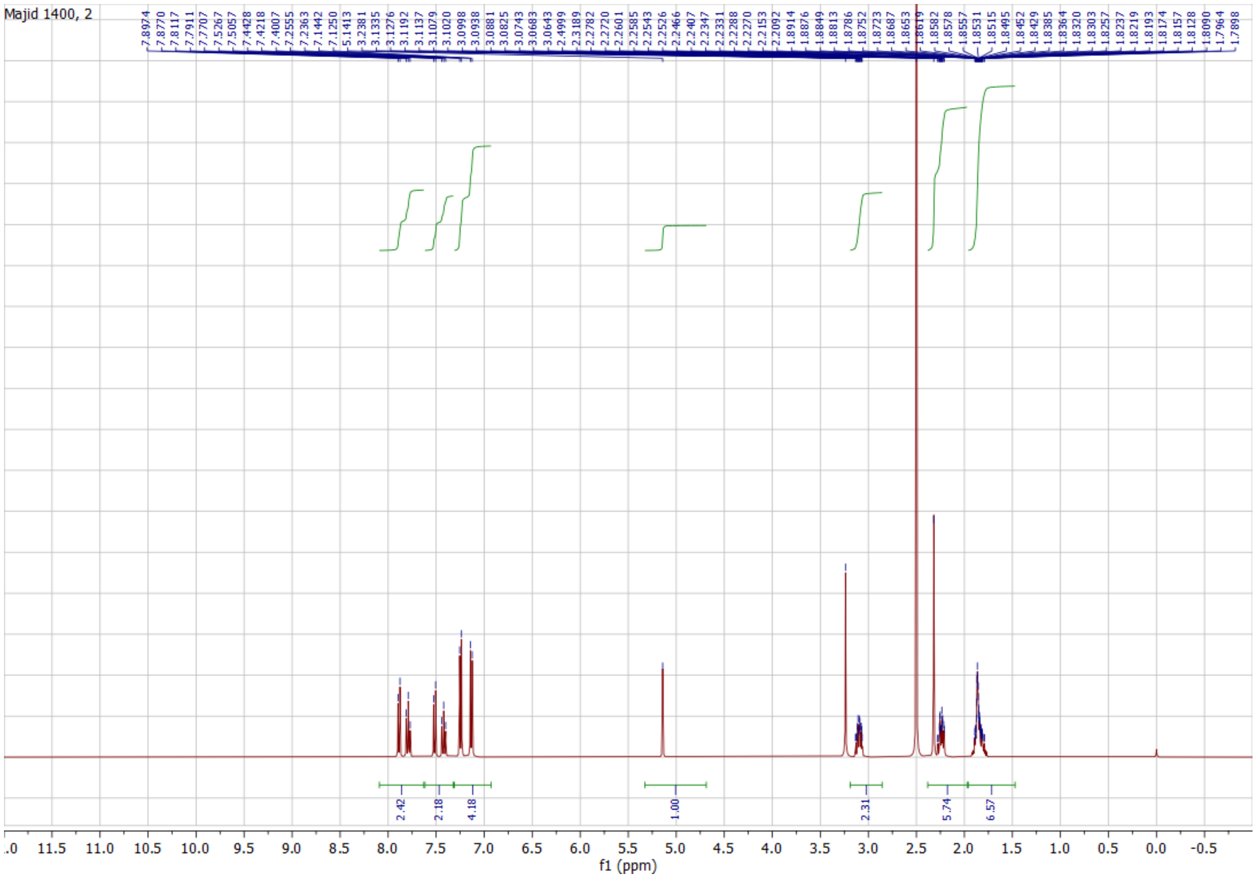

Figure S$_9$: ^{1}H-NMR spectra of 4-hydroxy-3-(piperidin-1-yl(p-tolyl)methyl)-2H-chromen-2-one

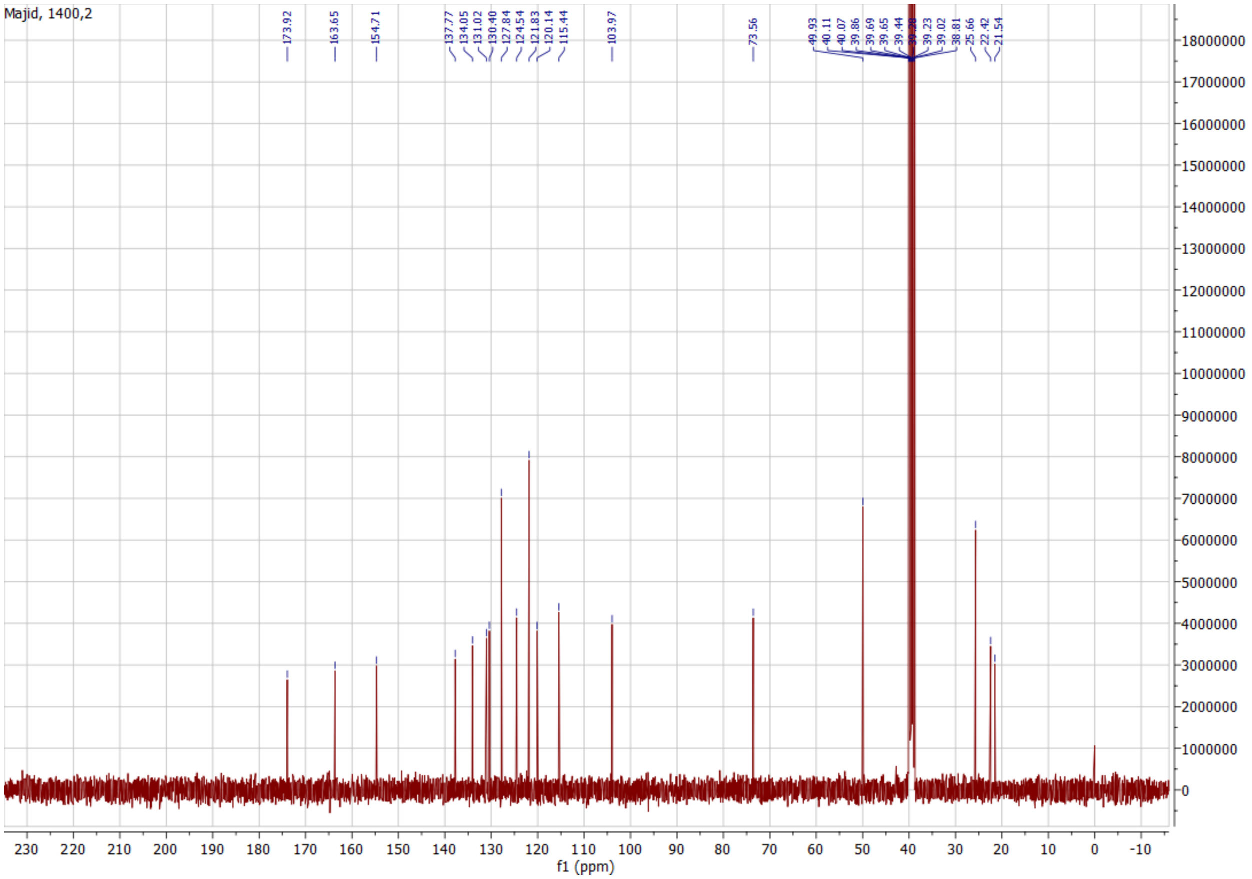

Figure S$_{10}$: ^{13}C-NMR spectra of 4-hydroxy-3-(piperidin-1-yl(p-tolyl)methyl)-2H-chromen-2-one

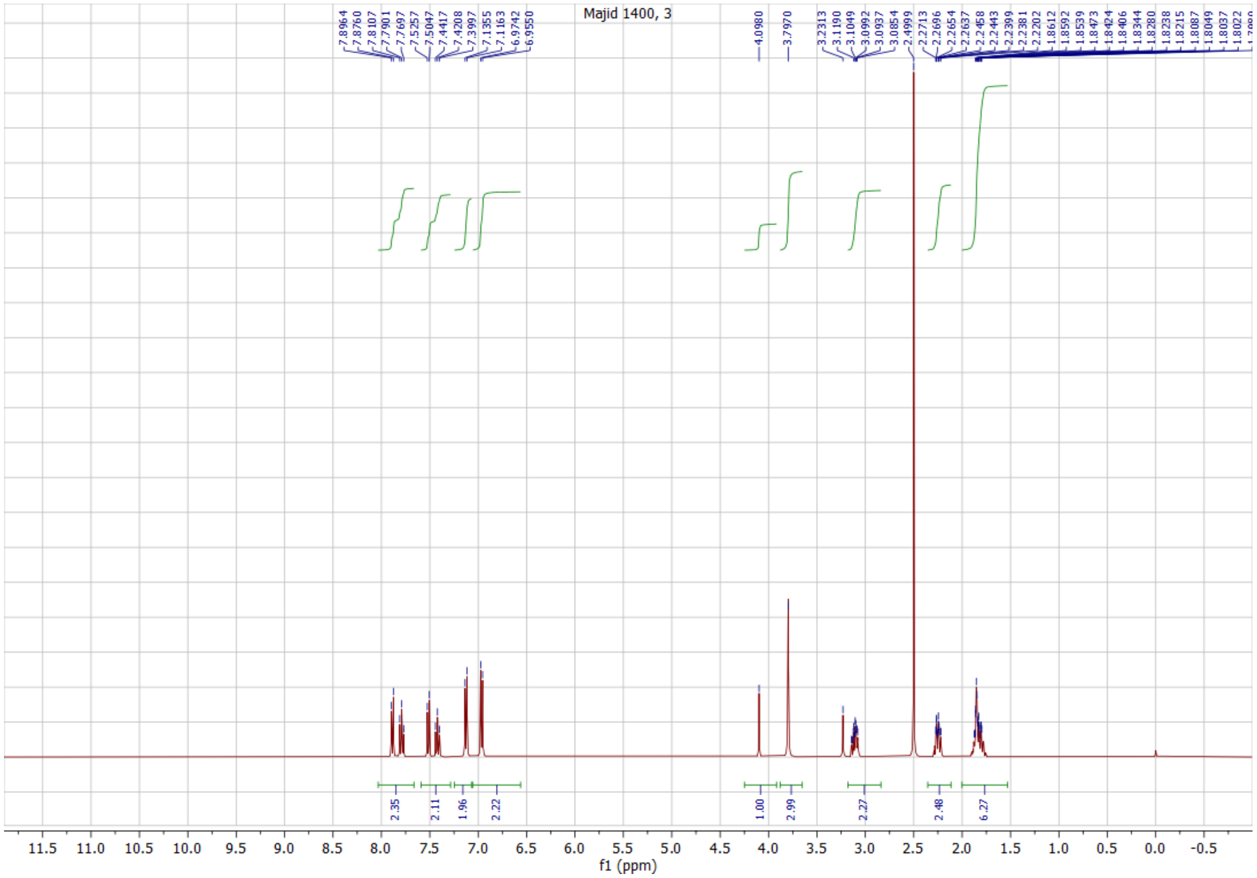

Figure S$_{11}$: ^{1}H-NMR spectra of 4-hydroxy-3-((4-methoxyphenyl)(piperidin-1-yl)methyl)-2H-chromen-2-one

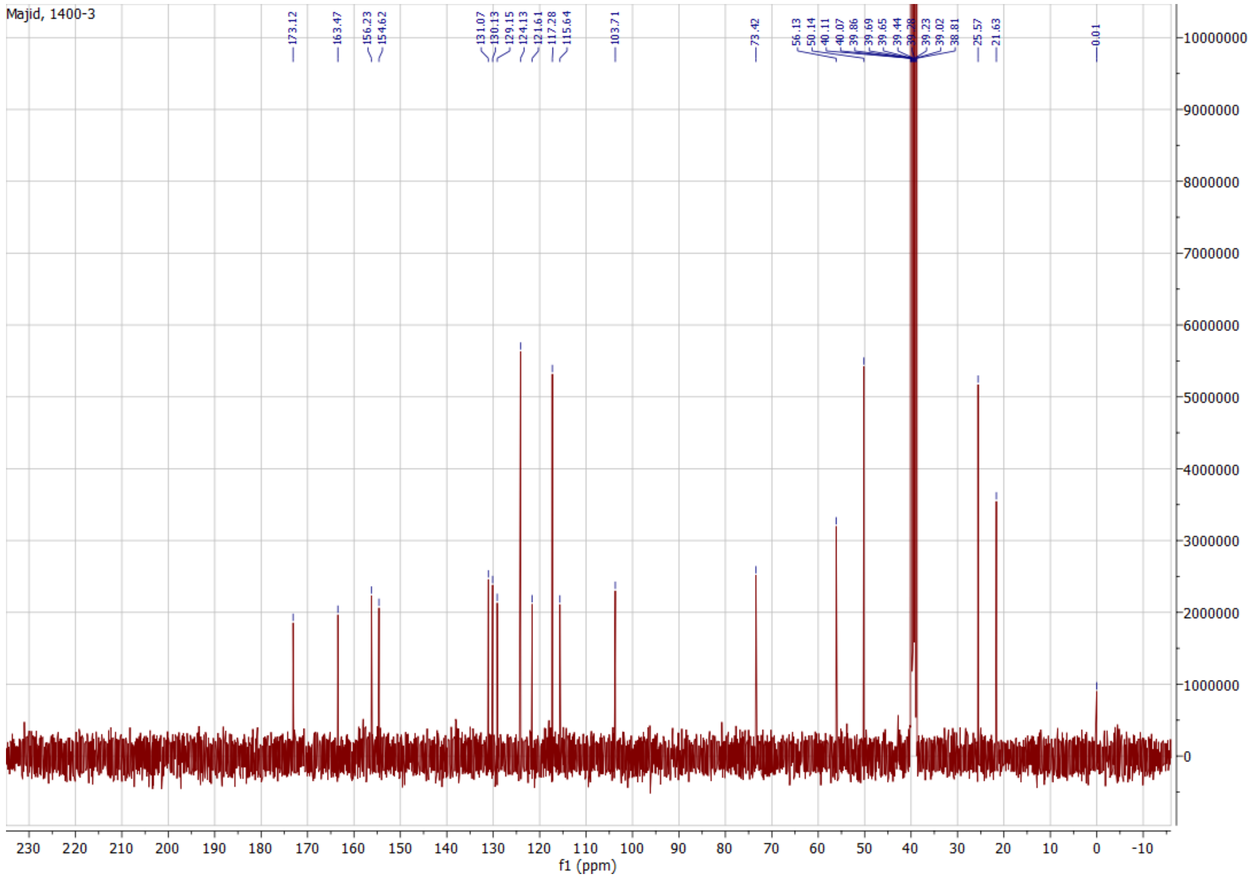

Figure S$_{12}$: ^{13}C-NMR spectra of 4-hydroxy-3-((4-methoxyphenyl)(piperidin-1-yl)methyl)-2H-chromen-2-one

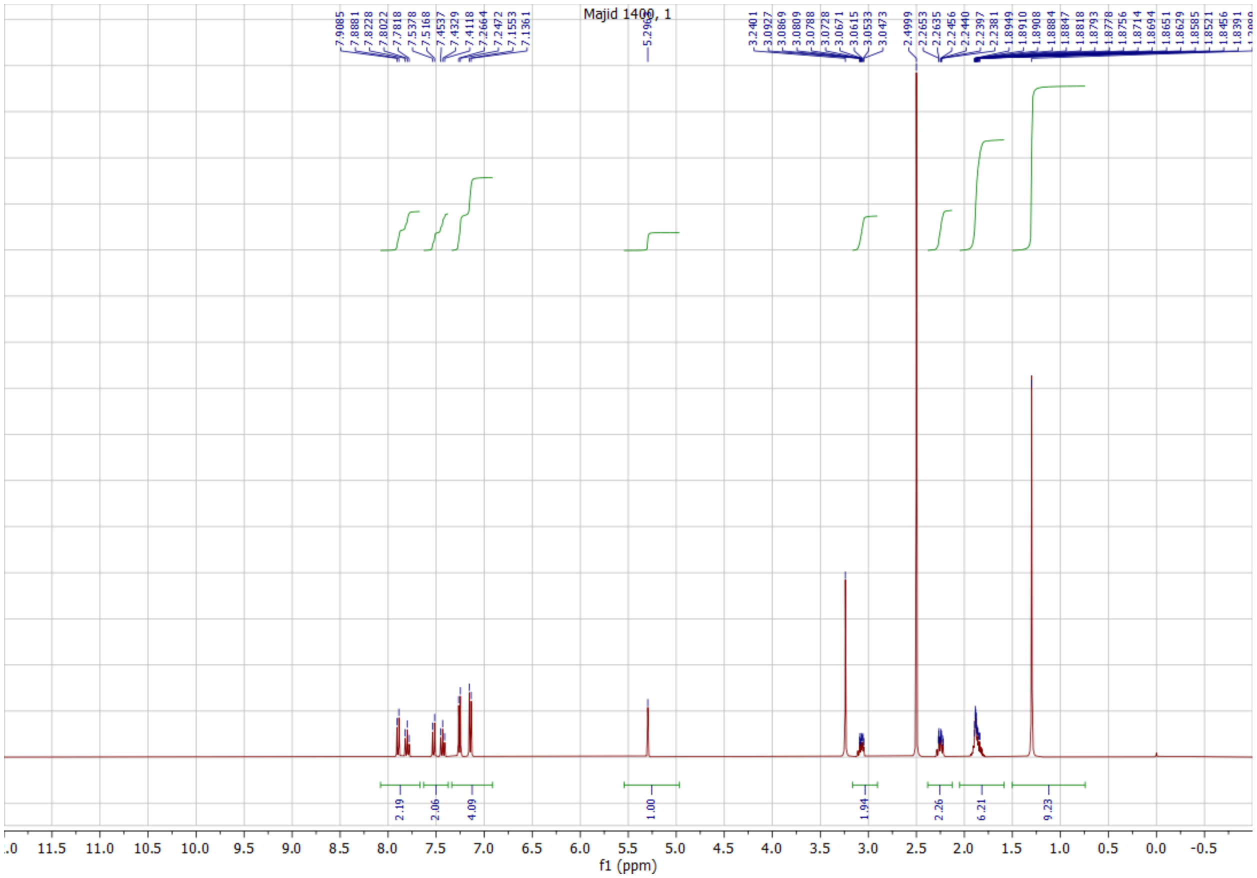

Figure S$_{13}$: ^{1}H-NMR spectra of 3-((4-(tert-butyl)phenyl)(piperidin-1-yl)methyl)-4-hydroxy-2H-chromen-2-one

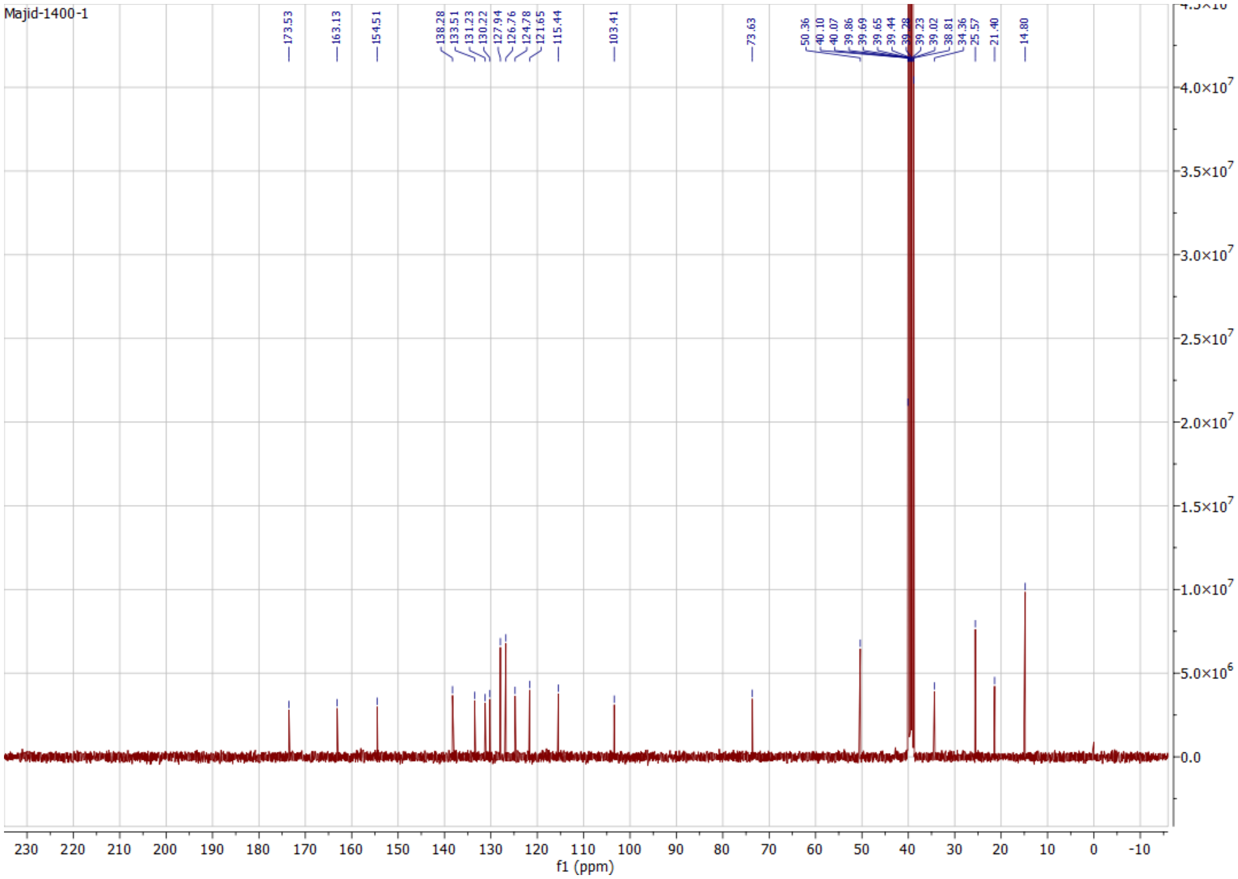

Figure S$_{14}$: ^{13}C-NMR spectra of 3-((4-(tert-butyl)phenyl)(piperidin-1-yl)methyl)-4-hydroxy-2H-chromen-2-one

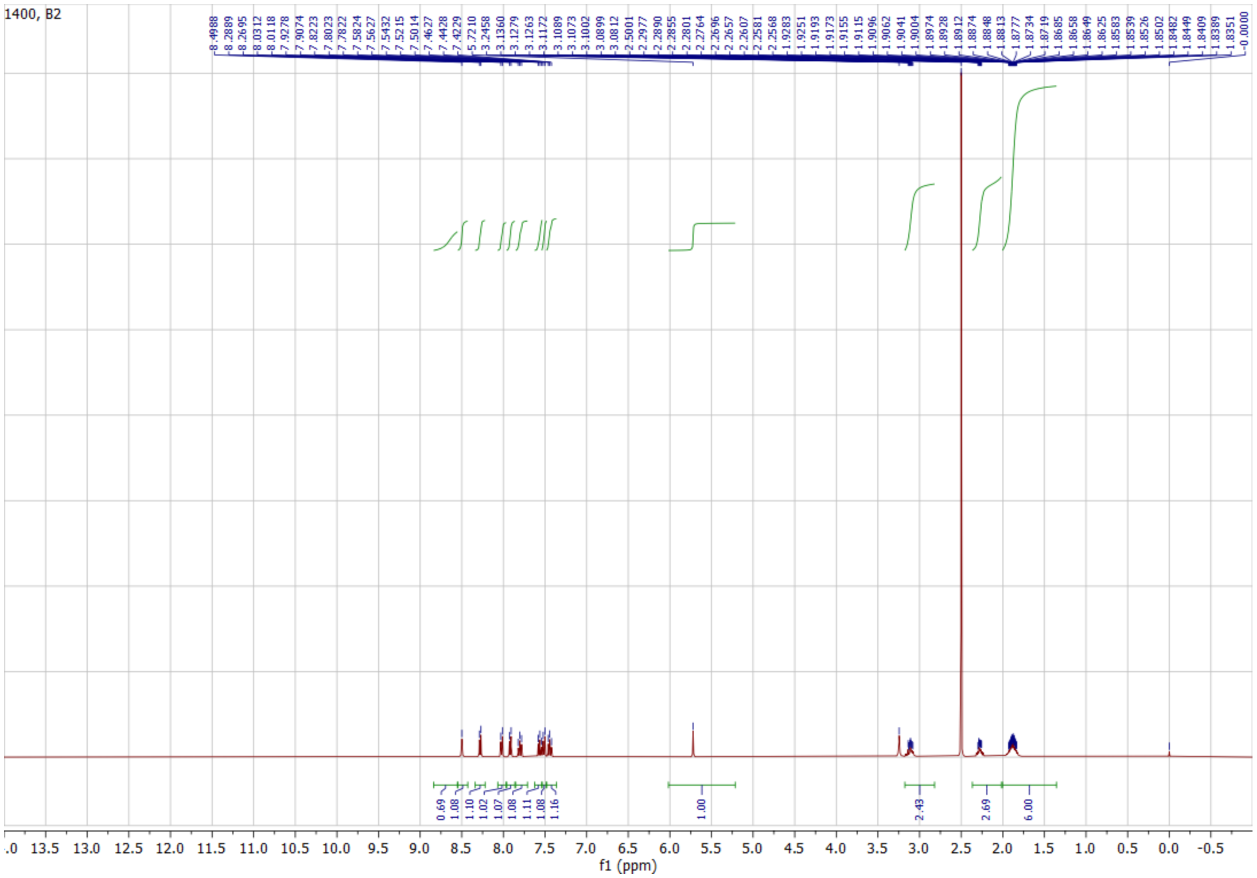

Figure S$_{15}$: ^{1}H-NMR spectra of 4-hydroxy-3-((3-nitrophenyl)(piperidin-1-yl)methyl)-2H-chromen-2-one

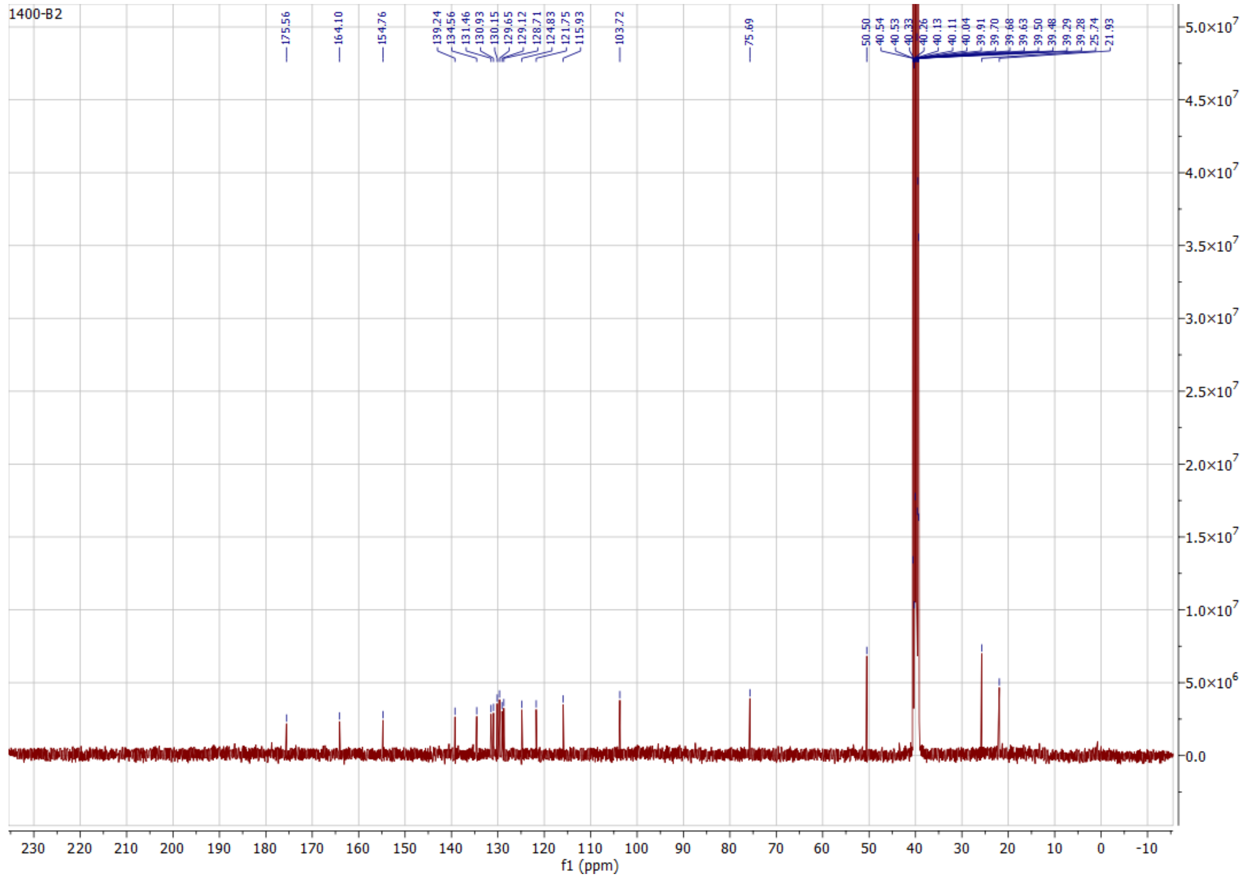

Figure S$_{16}$: ^{13}C-NMR spectra of 4-hydroxy-3-((3-nitrophenyl)(piperidin-1-yl)methyl)-2H-chromen-2-one

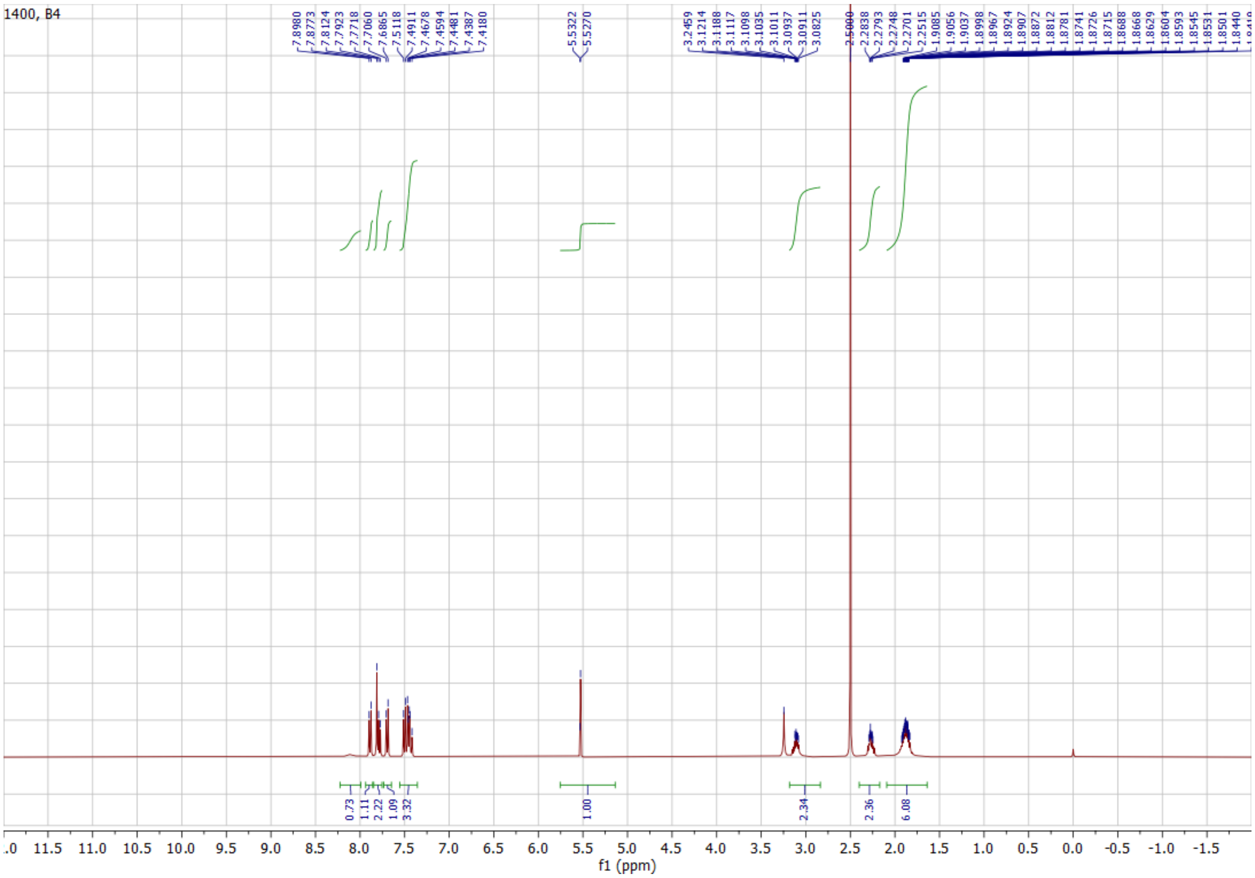

Figure S_{17}: H-NMR spectra of 3-((2,4-dichlorophenyl)(piperidin-1-yl)methyl)-4-hydroxy-2H-chromen-2-one

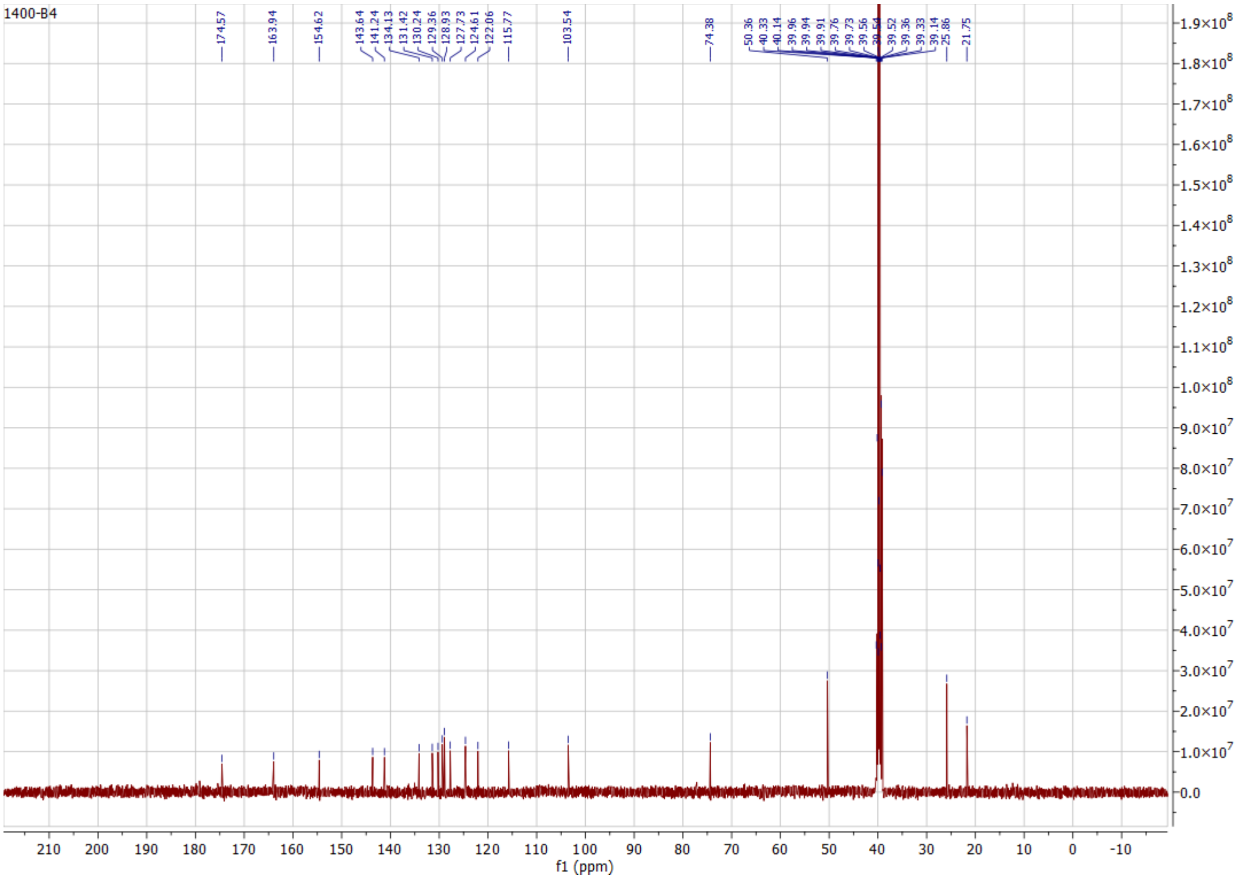

Figure S$_{18}$: ^{13}C-NMR spectra of 3-((2,4-dichlorophenyl)(piperidin-1-yl)methyl)-4-hydroxy-2H-chromen-2-one

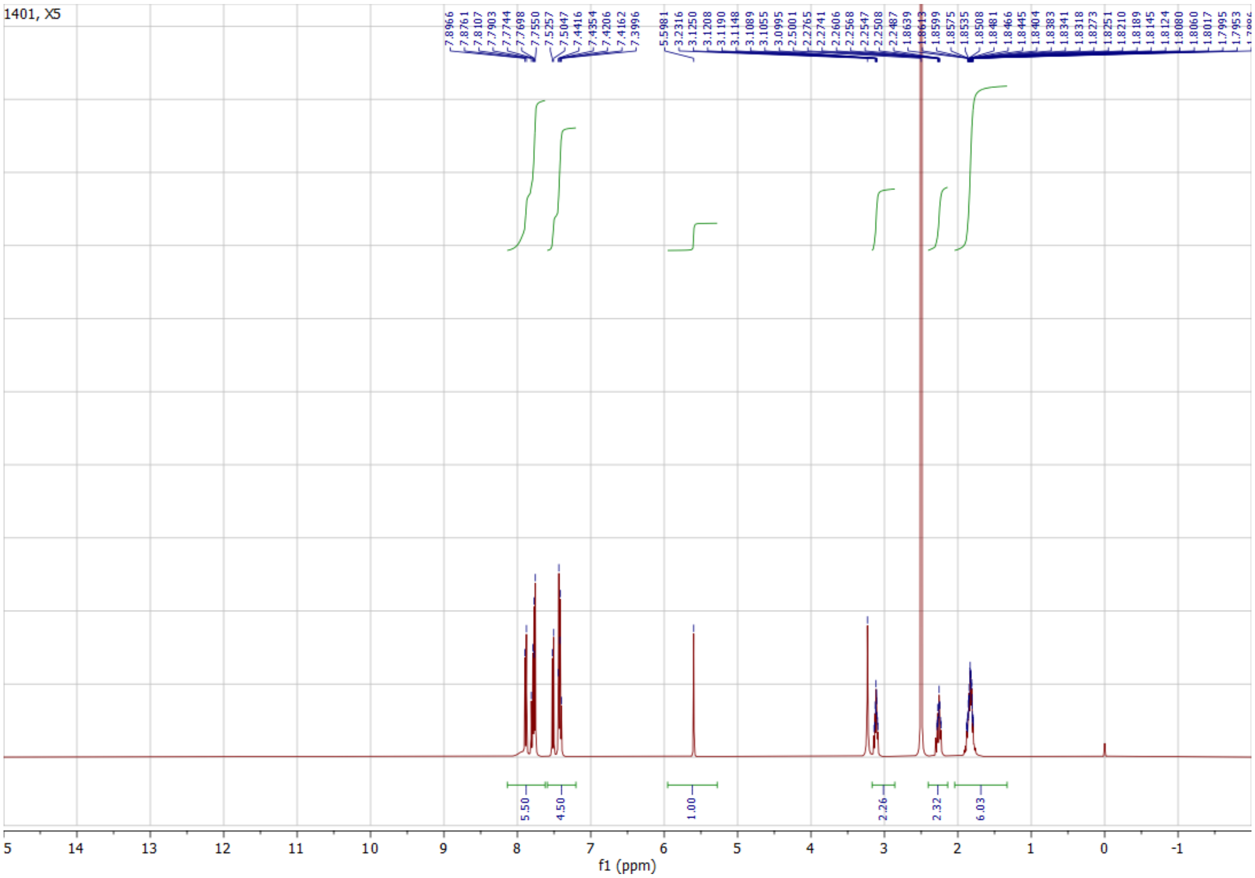

Figure S$_{19}$: H-NMR spectra of 3-((4-bromophenyl)(piperidin-1-yl)methyl)-4-hydroxy-2H-chromen-2-one

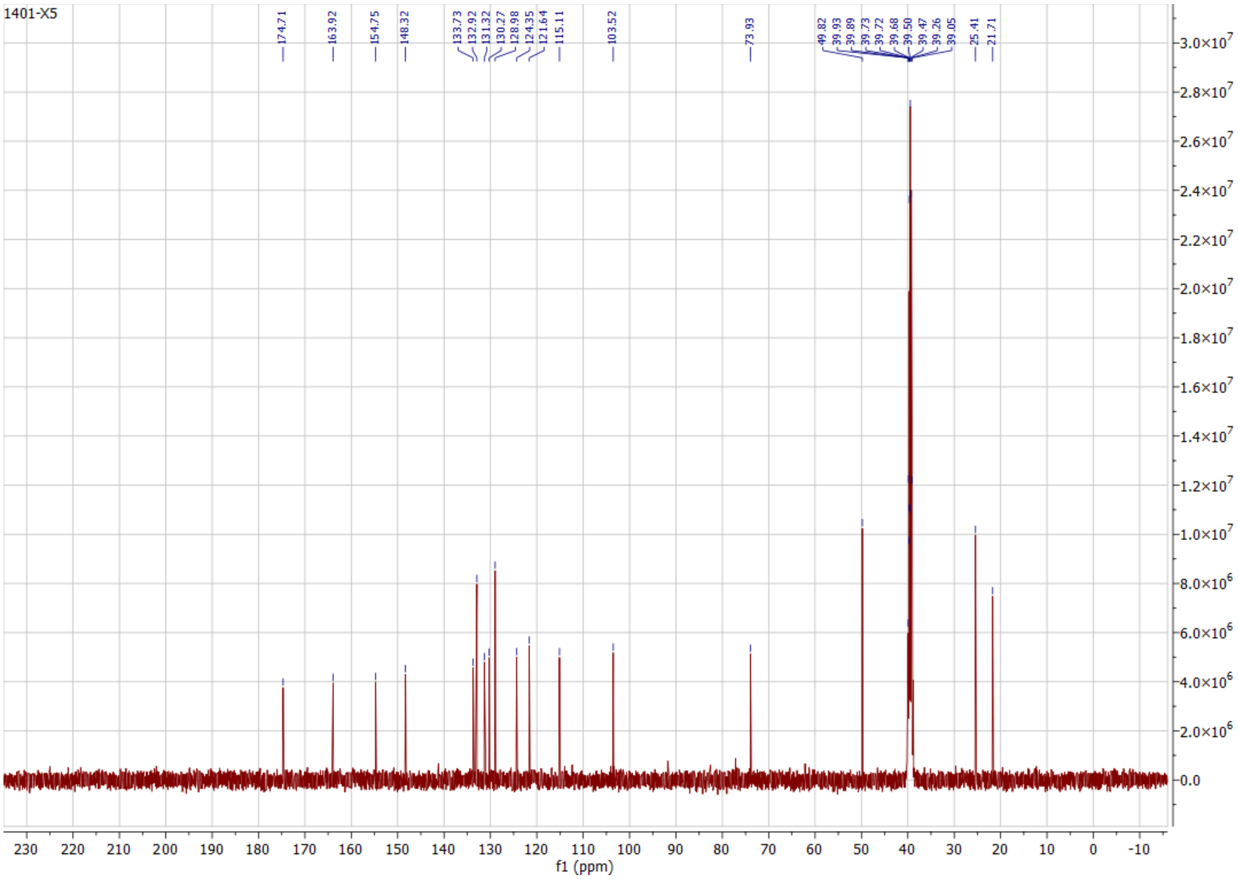

Figure S$_{20}$: ^{13}C-NMR spectra of 3-((4-bromophenyl)(piperidin-1-yl)methyl)-4-hydroxy-2H-chromen-2-one

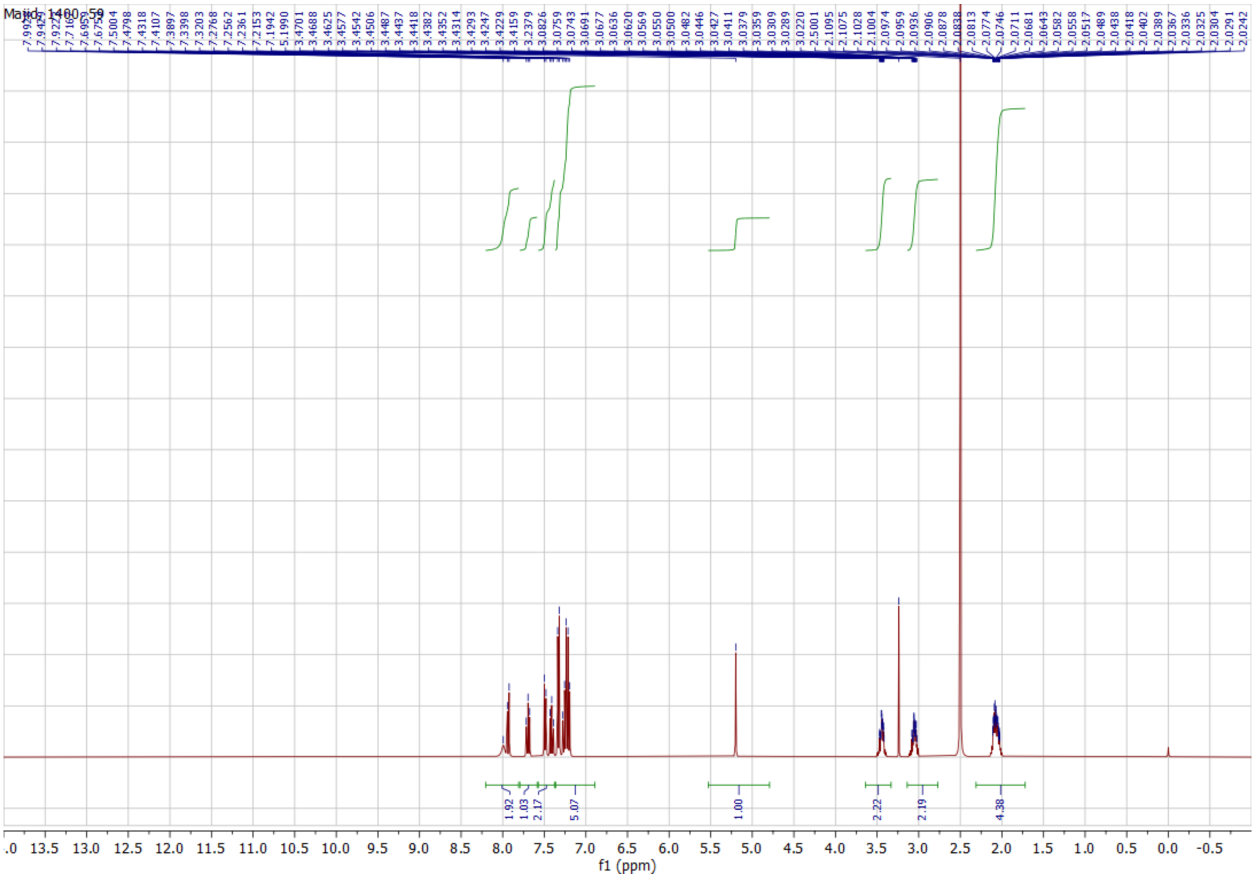

Figure S_{21}: H-NMR spectra of 4-hydroxy-3-(phenyl(pyrrolidin-1-yl)methyl)-2H-chromen-2-one

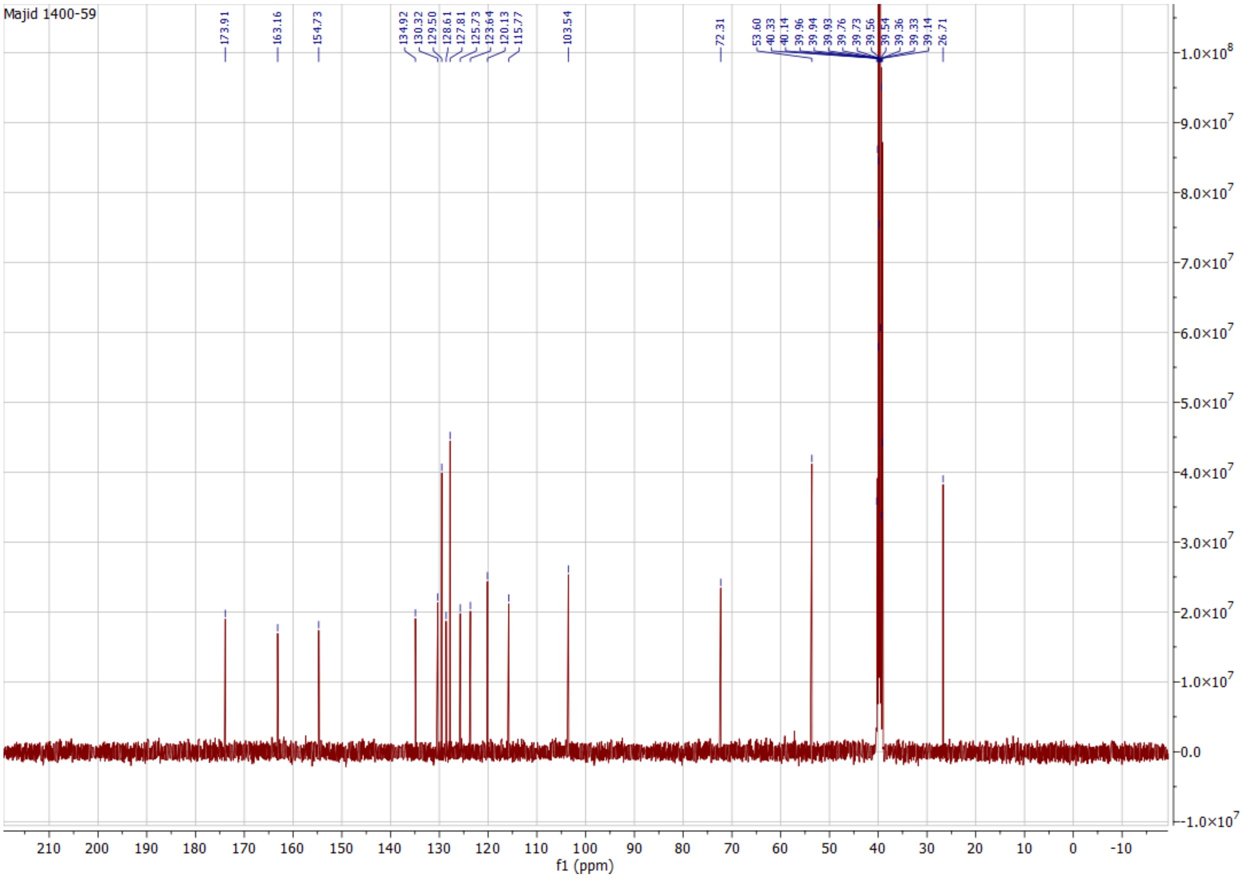

Figure S$_{22}$: ^{13}C-NMR spectra of 4-hydroxy-3-(phenyl(pyrrolidin-1-yl)methyl)-2H-chromen-2-one

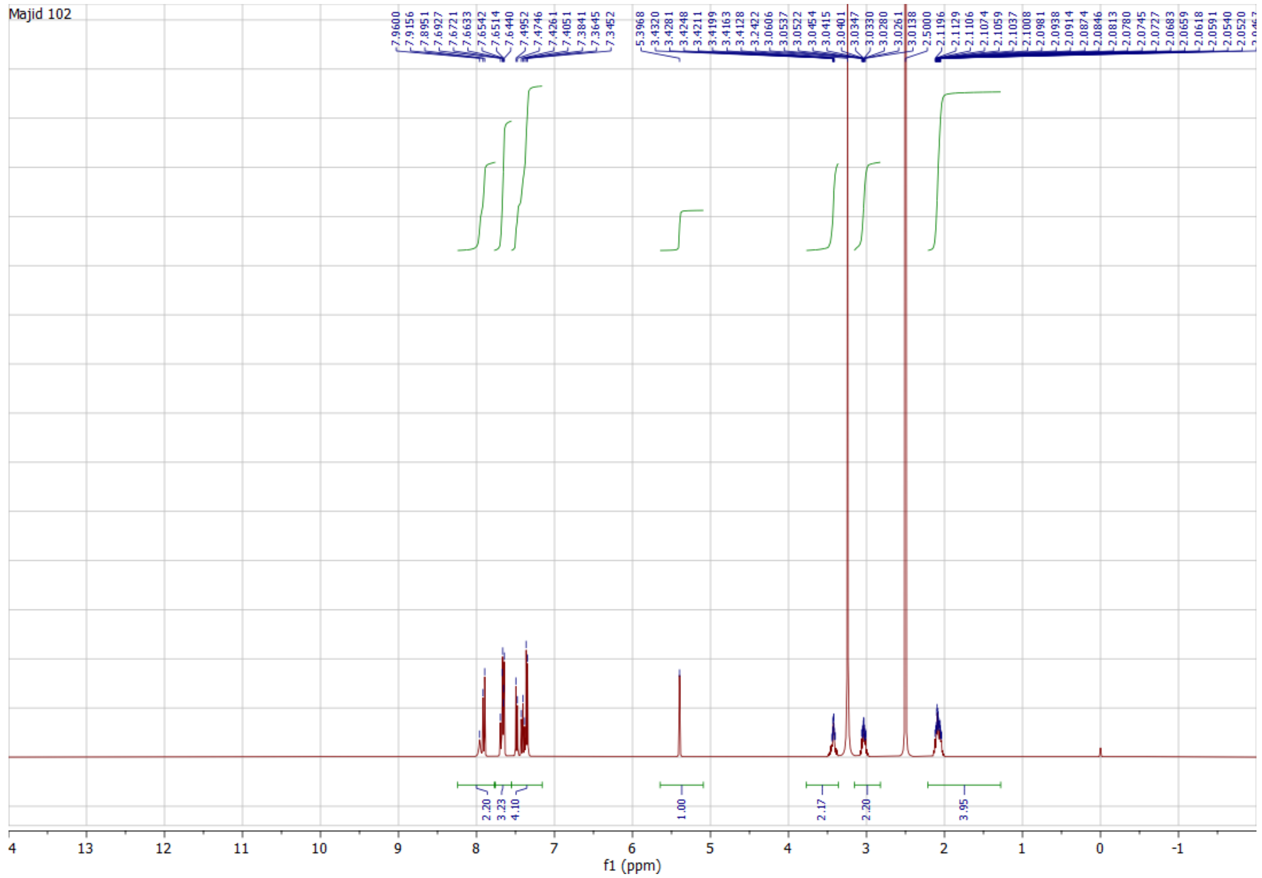

Figure S23: H-NMR spectra of 3-((4-bromophenyl)(pyrrolidin-1-yl)methyl)-4-hydroxy-2H-chromen-2-one

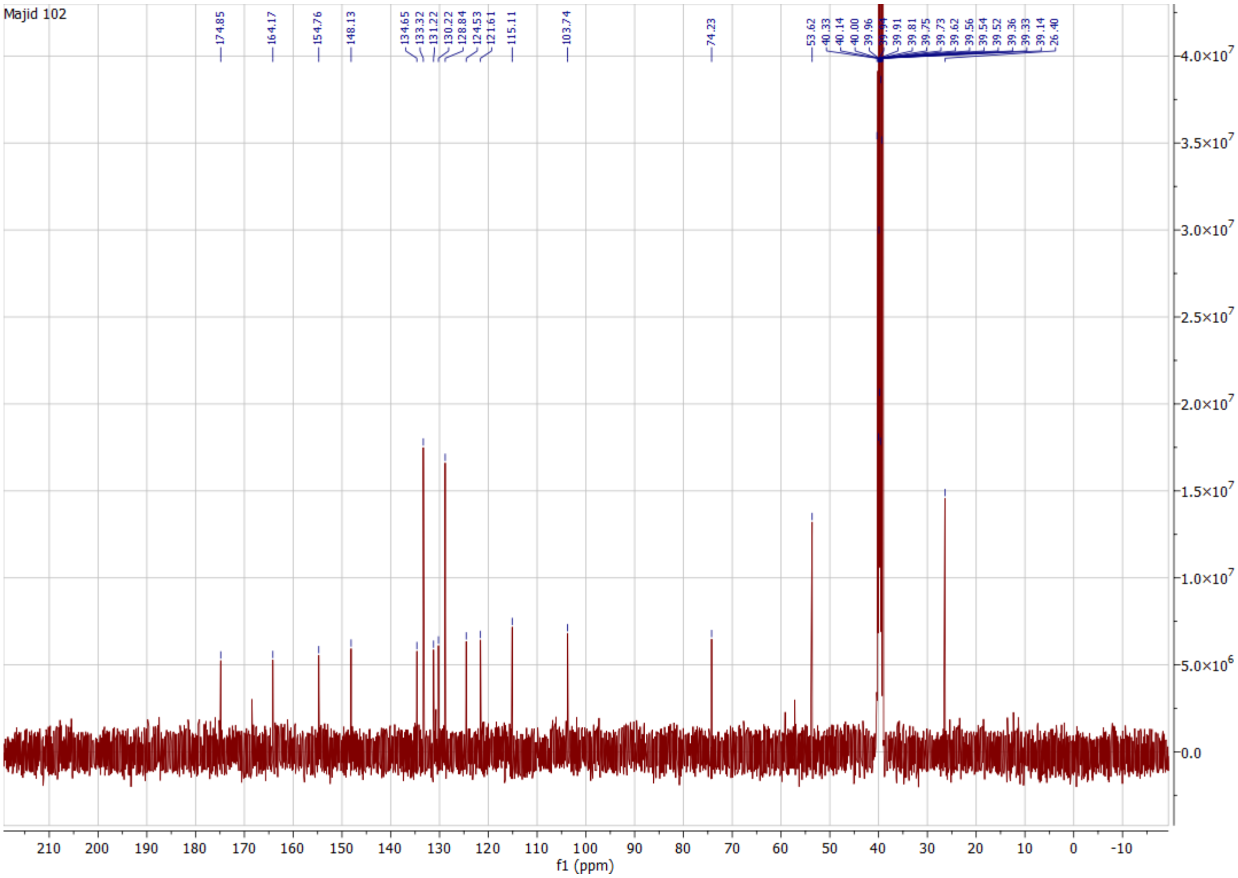

Figure S$_{24}$: ^{13}C-NMR spectra of 3-((4-bromophenyl)(pyrrolidin-1-yl)methyl)-4-hydroxy-2H-chromen-2-one

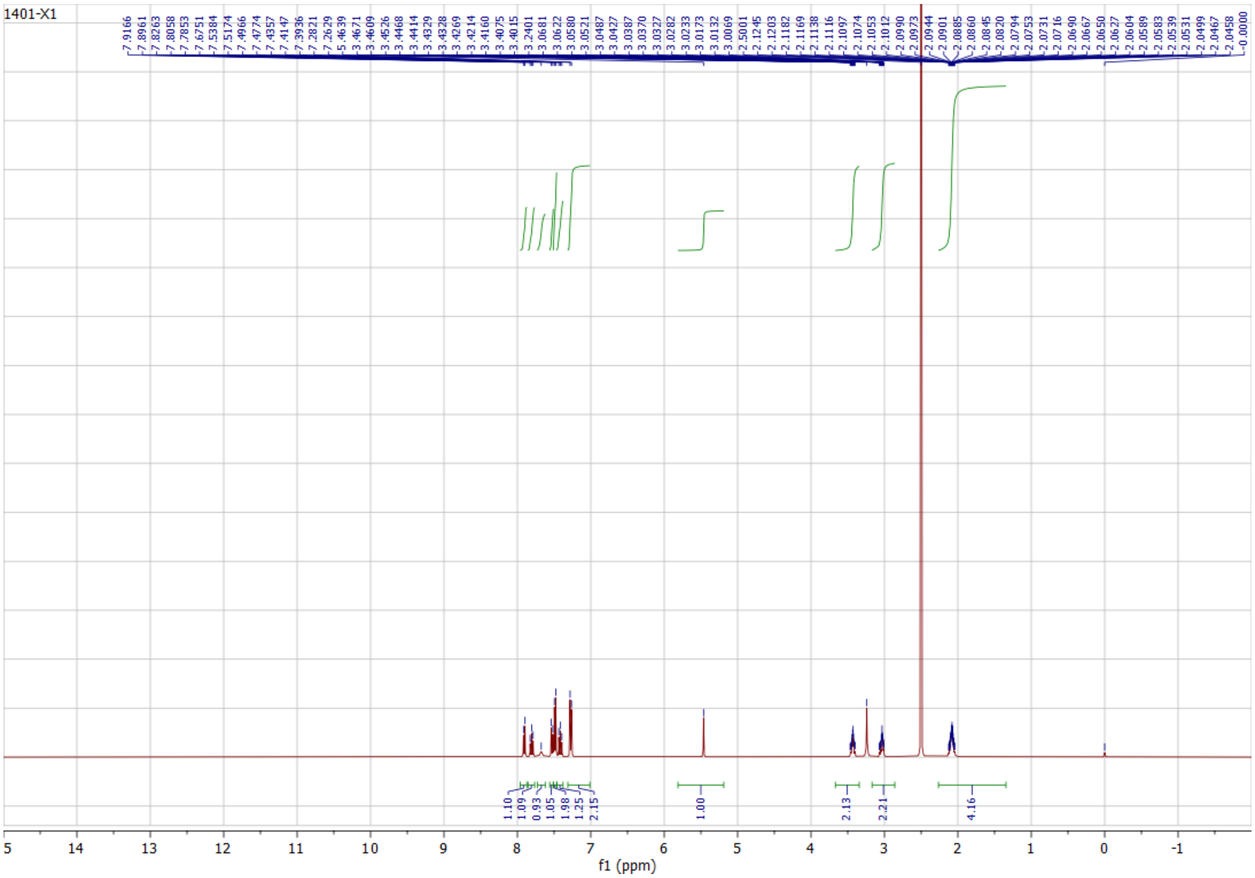

Figure S_{25}: H-NMR spectra of 3-((4-chlorophenyl)(pyrrolidin-1-yl)methyl)-4-hydroxy-2H-chromen-2-one

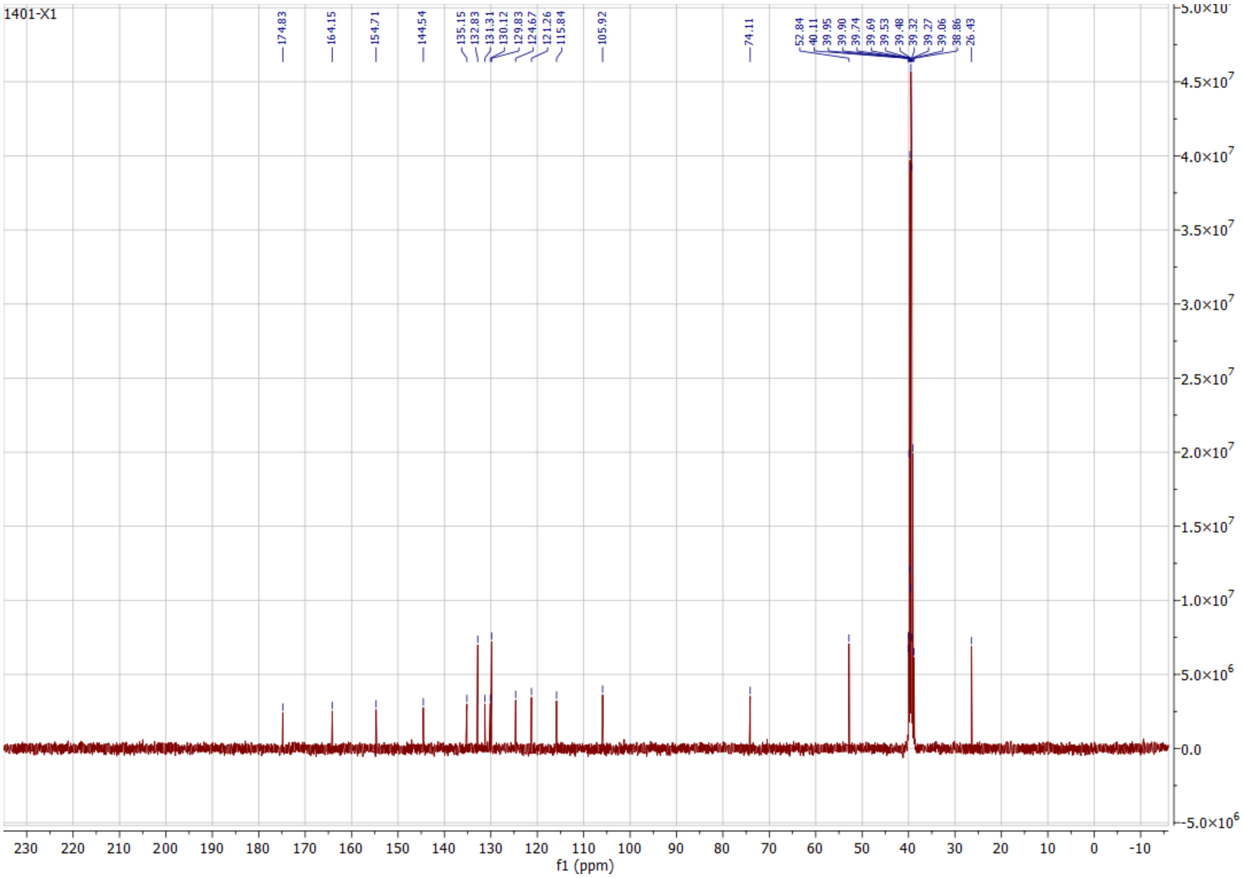

Figure S$_{26}$: ^{13}C-NMR spectra of 3-((4-chlorophenyl)(pyrrolidin-1-yl)methyl)-4-hydroxy-2H-chromen-2-one

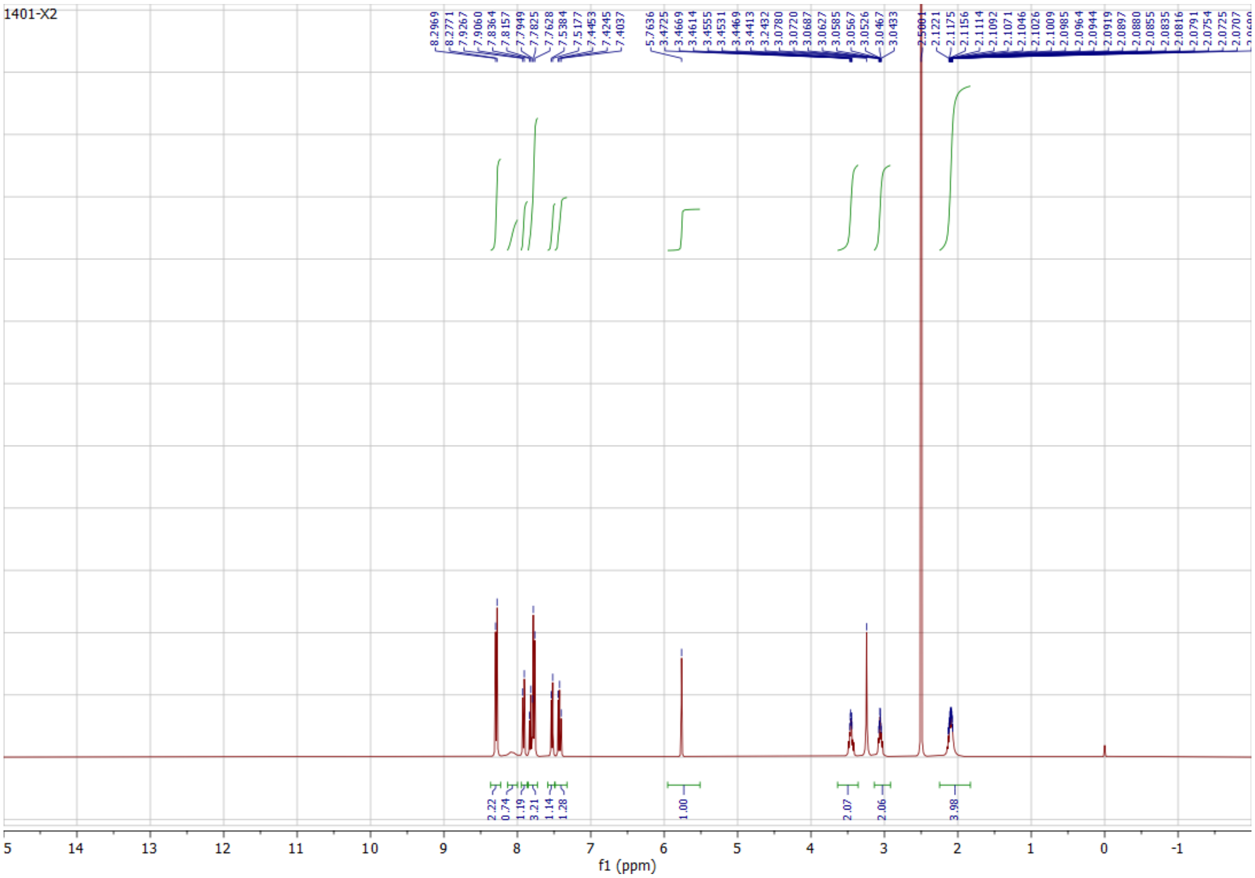

Figure S_{27}: ^{1}H-NMR spectra of 4-hydroxy-3-((4-nitrophenyl)(pyrrolidin-1-yl)methyl)-2H-chromen-2-one

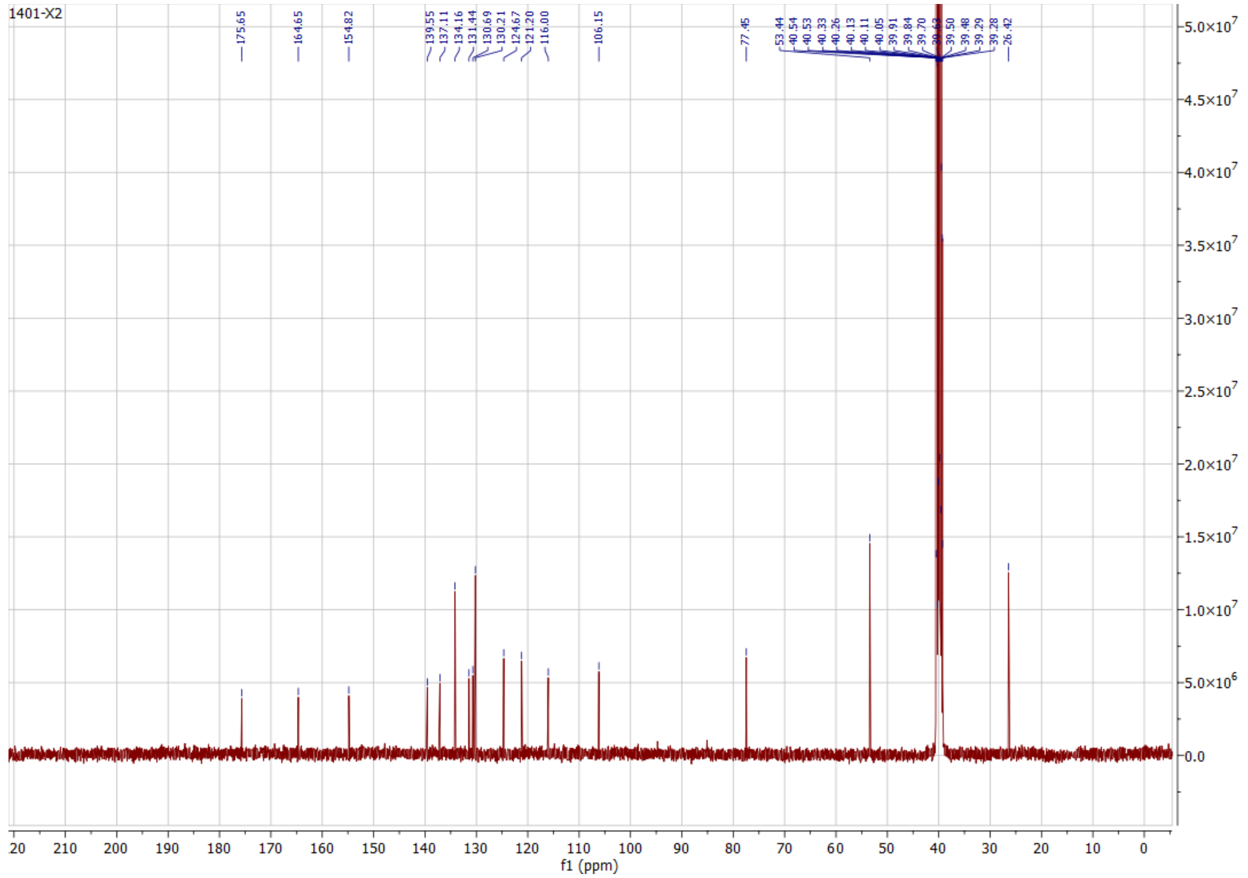

Figure S_{28}: ^{13}C-NMR spectra of 4-hydroxy-3-((4-nitrophenyl)(pyrrolidin-1-yl)methyl)-2H-chromen-2-one

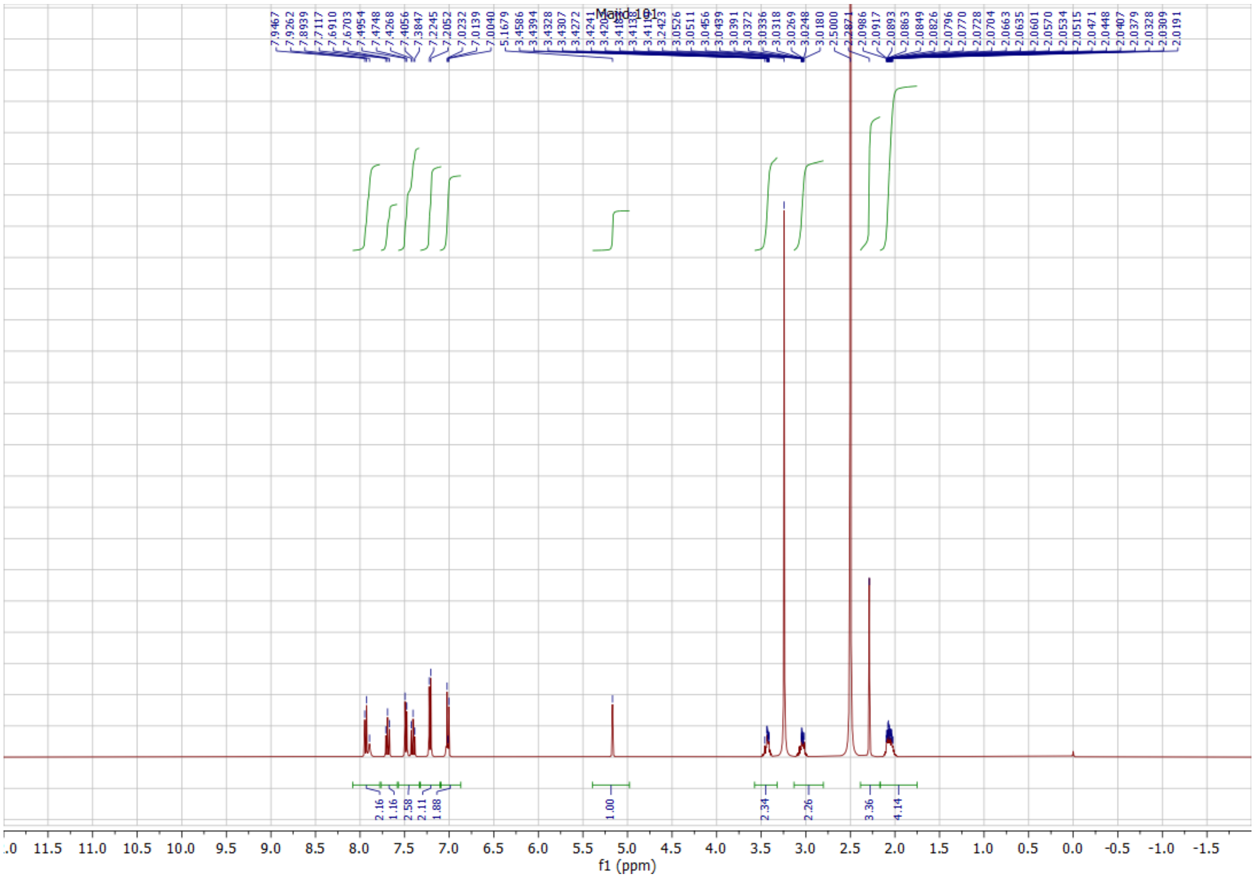

Figure S$_{29}$: H-NMR spectra of 4-hydroxy-3-(pyrrolidin-1-yl(p-tolyl)methyl)-2H-chromen-2-one

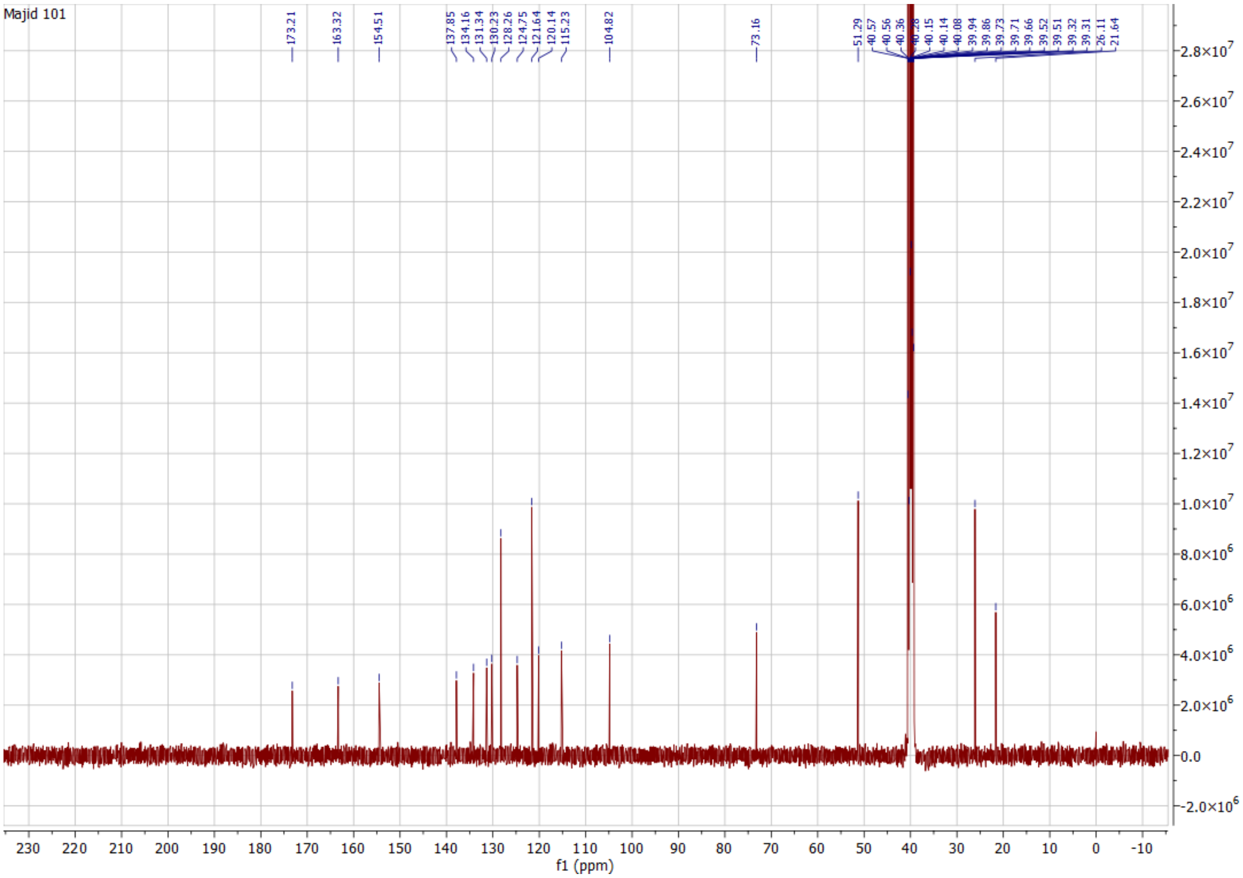

Figure S$_{30}$: ^{13}C-NMR spectra of 4-hydroxy-3-(pyrrolidin-1-yl(p-tolyl)methyl)-2H-chromen-2-one

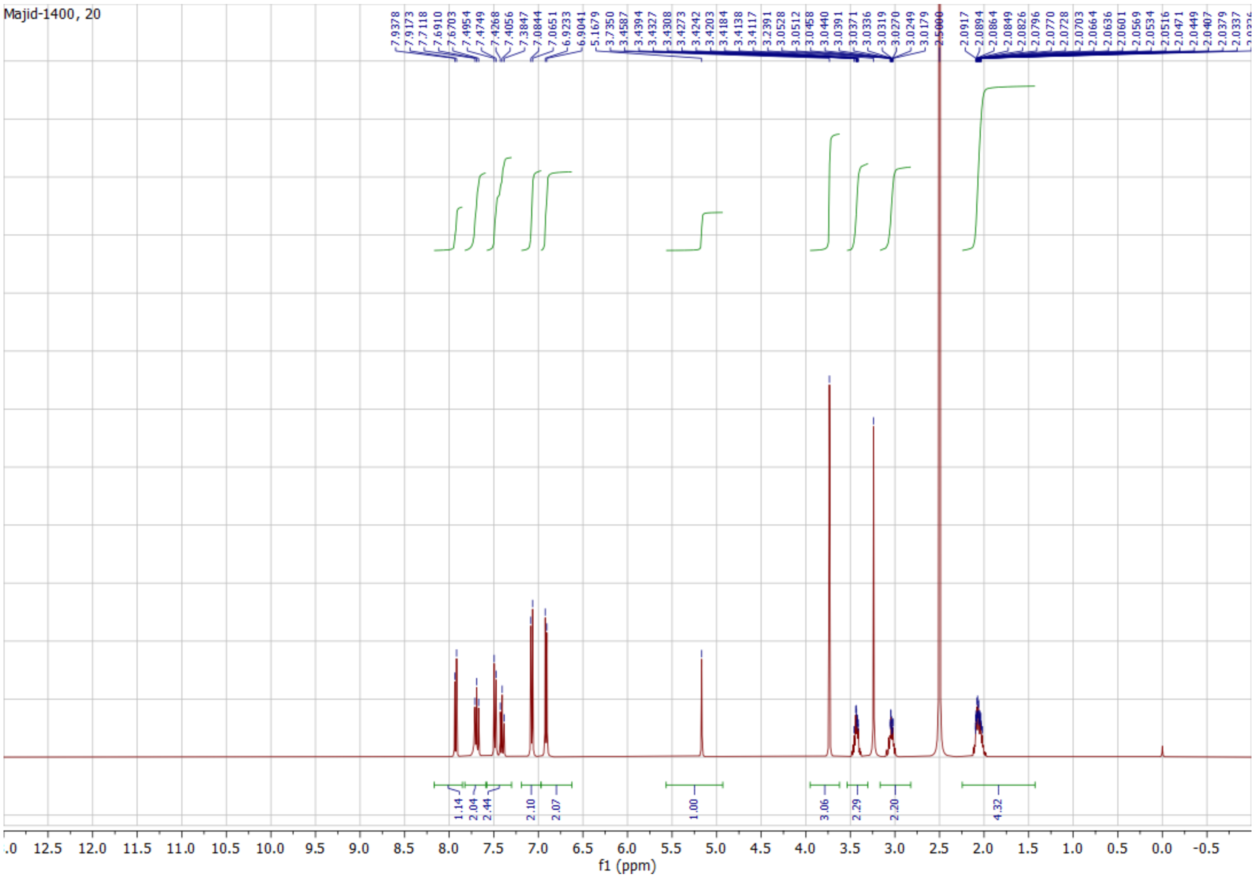

Figure S_{31}: H-NMR spectra of 4-hydroxy-3-((4-methoxyphenyl)(pyrrolidin-1-yl)methyl)-2H-chromen-2-one

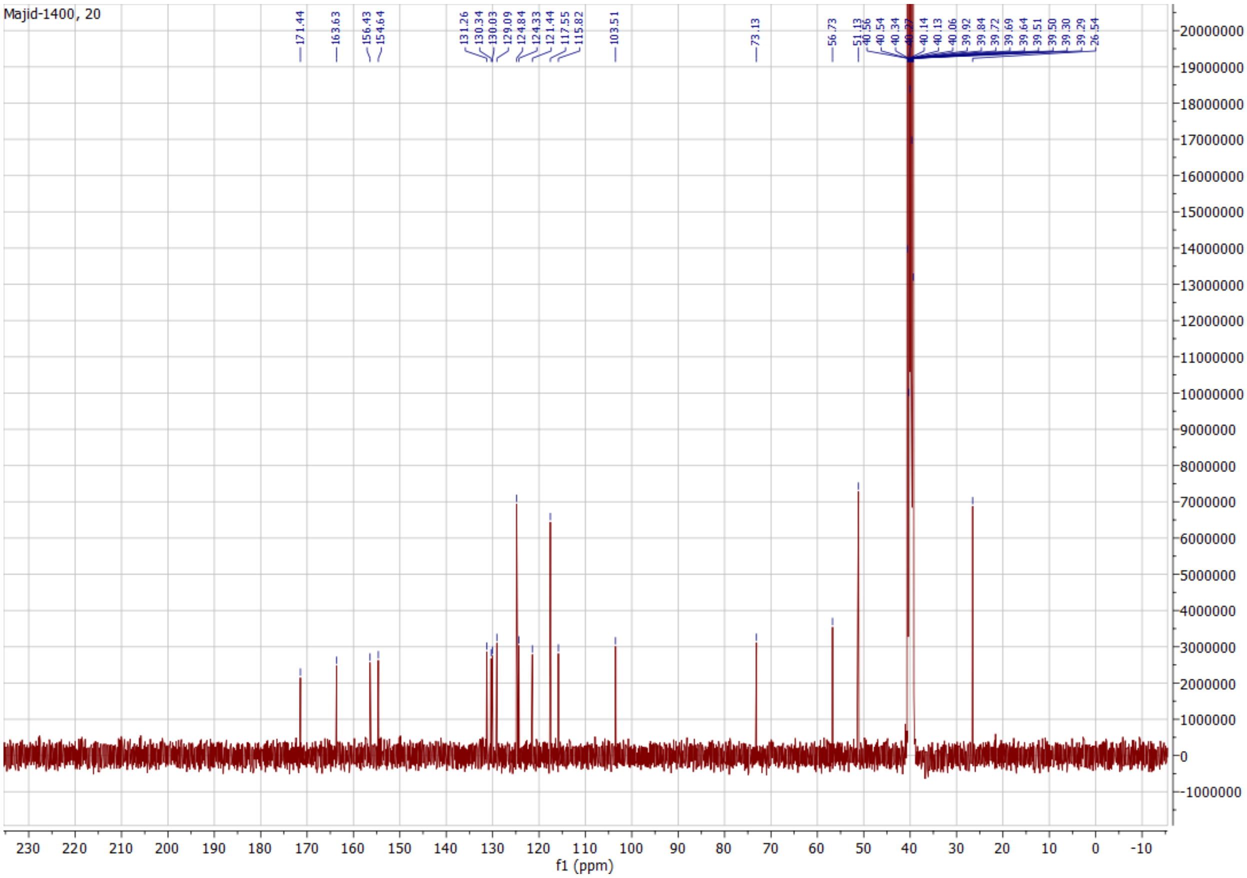

Figure S$_{32}$: ^{13}C-NMR spectra of 4-hydroxy-3-((4-methoxyphenyl)(pyrrolidin-1-yl)methyl)-2H-chromen-2-one

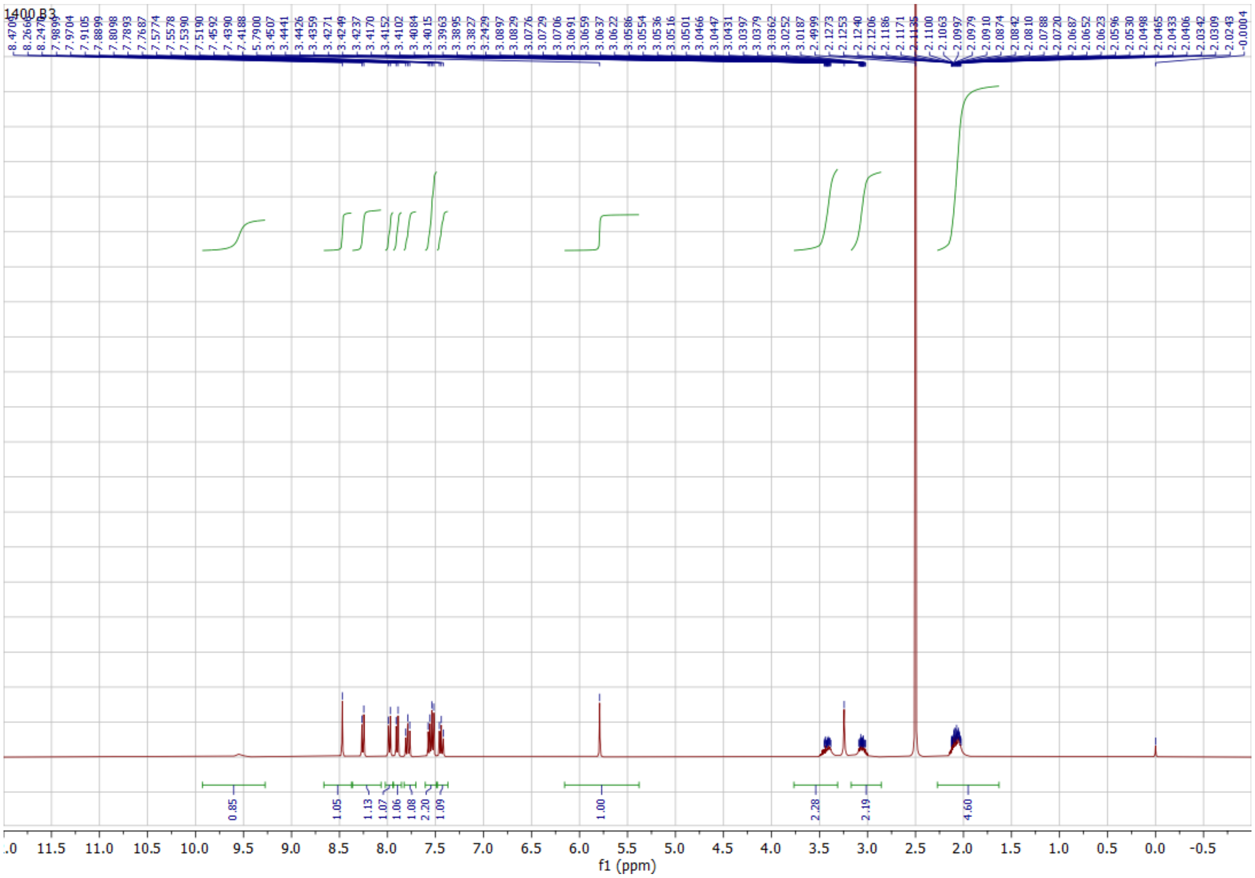

Figure S$_{33}$: ^{1}H-NMR spectra of 4-hydroxy-3-((3-nitrophenyl)(pyrrolidin-1-yl)methyl)-2H-chromen-2-one

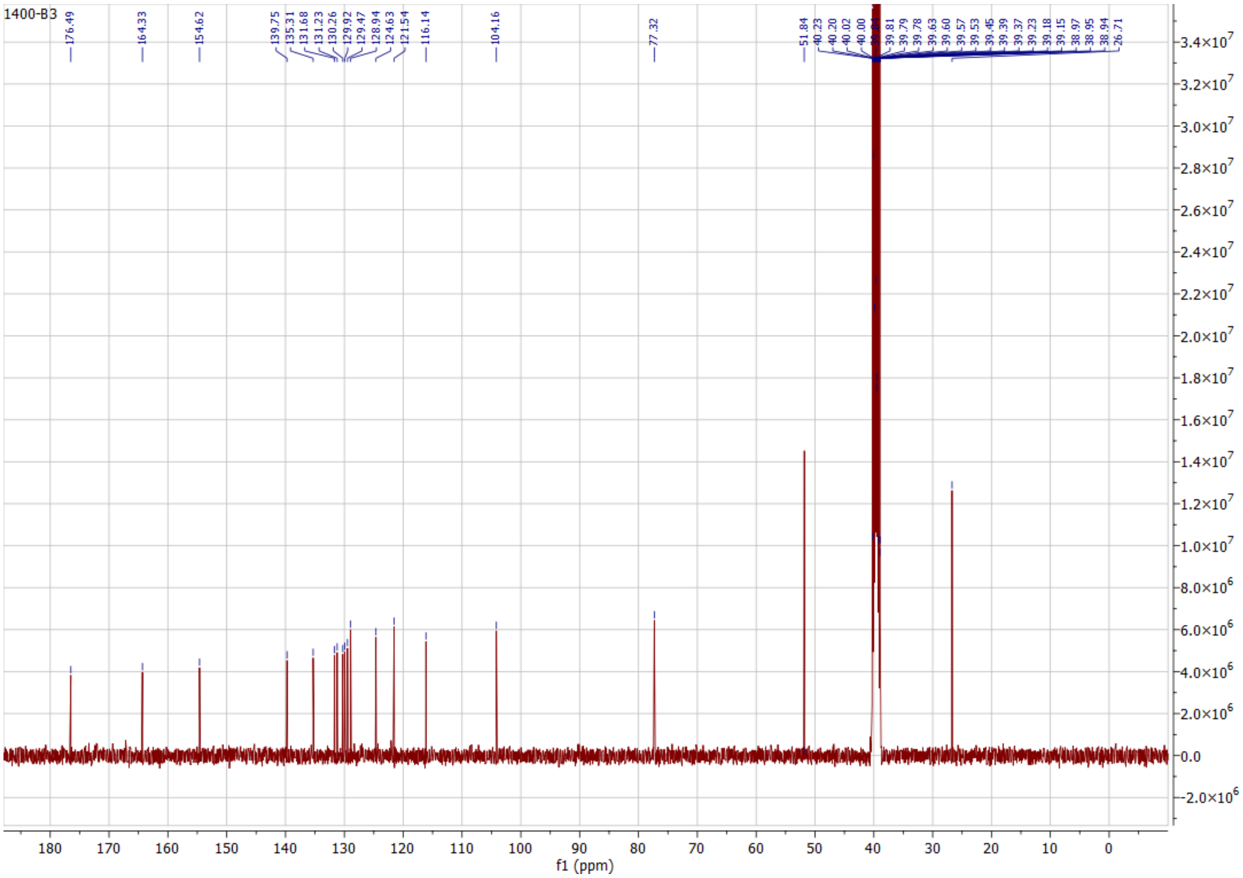

Figure S34: ^{13}C-NMR spectra of 4-hydroxy-3-((3-nitrophenyl)(pyrrolidin-1-yl)methyl)-2H-chromen-2-one

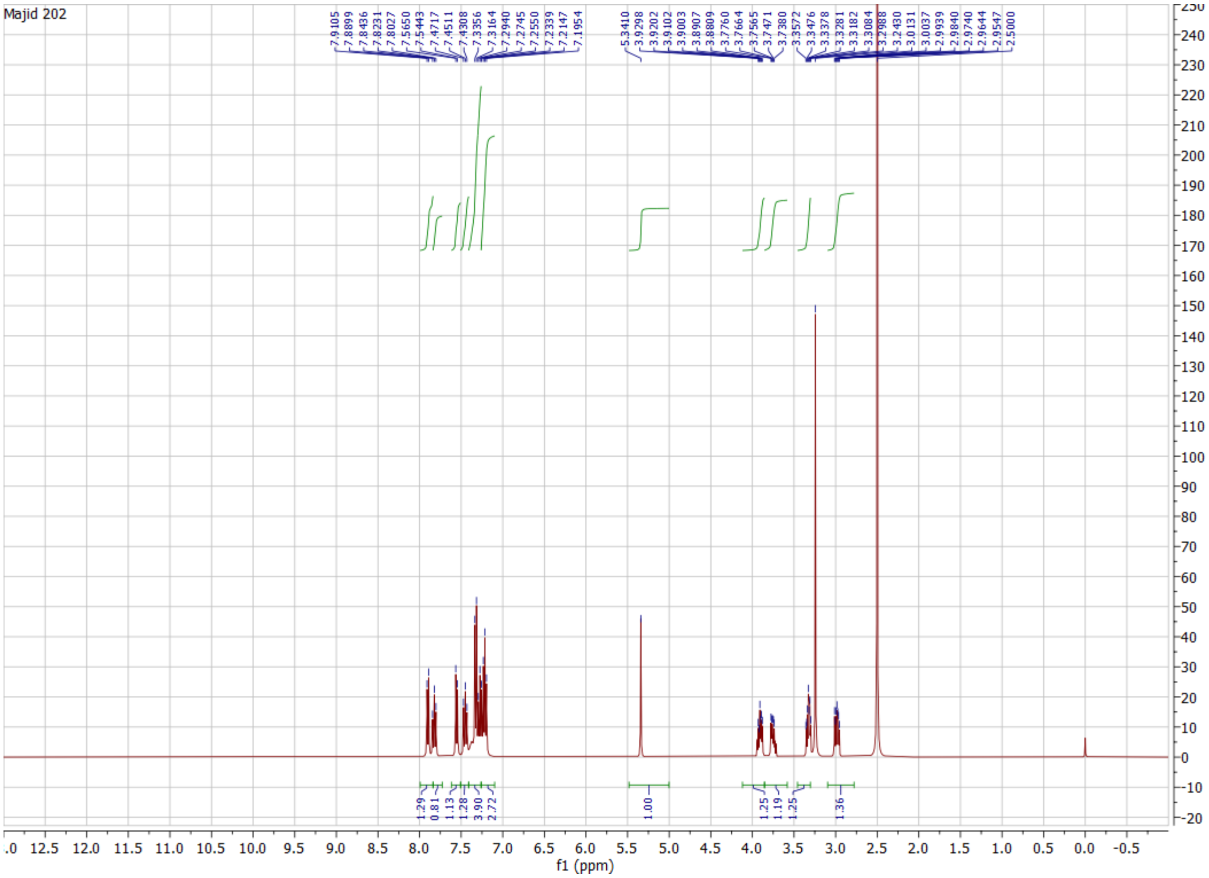

Figure S_{35}: ^{1}H-NMR spectra of 4-hydroxy-3-(morpholino(phenyl)methyl)-2H-chromen-2-one

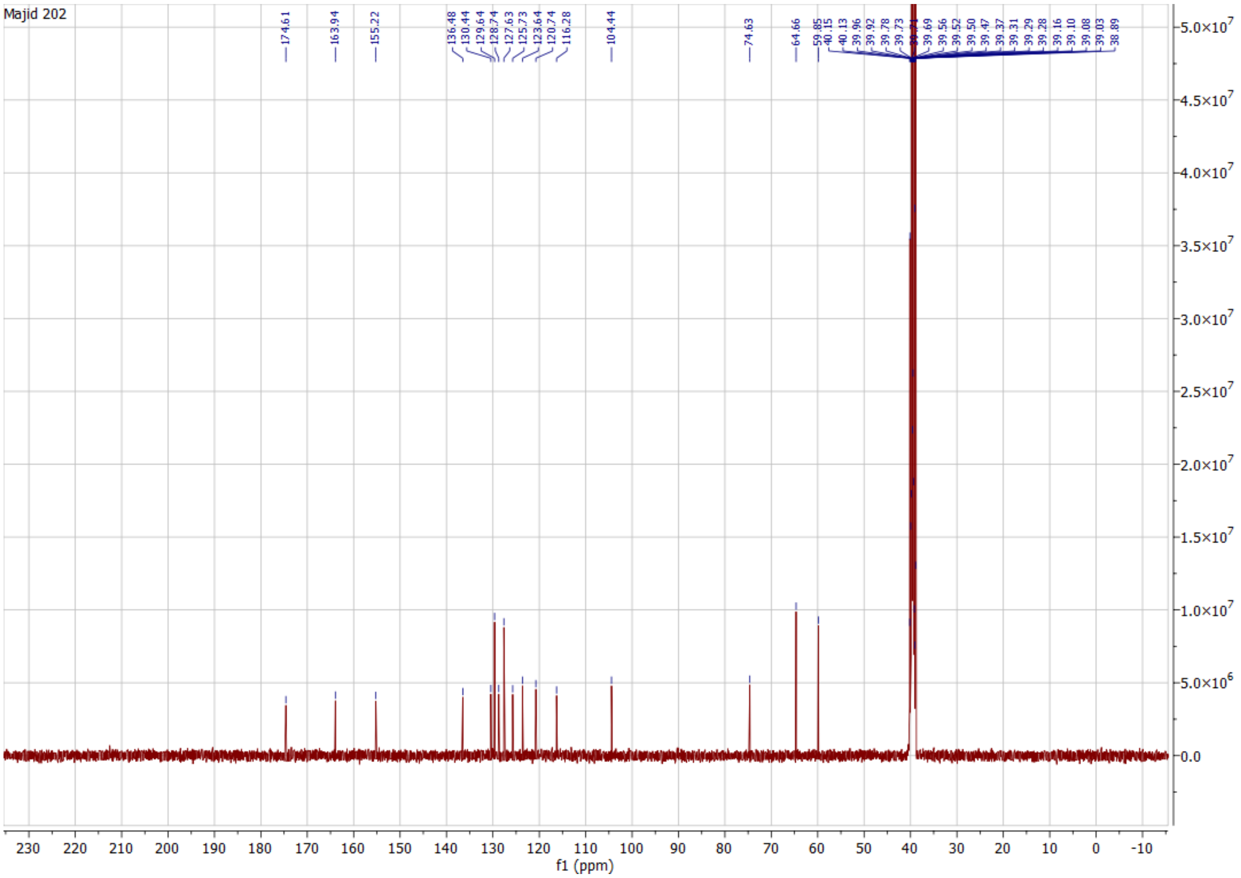

Figure S$_{36}$: ^{13}C-NMR spectra of 4-hydroxy-3-(morpholino(phenyl)methyl)-2H-chromen-2-one

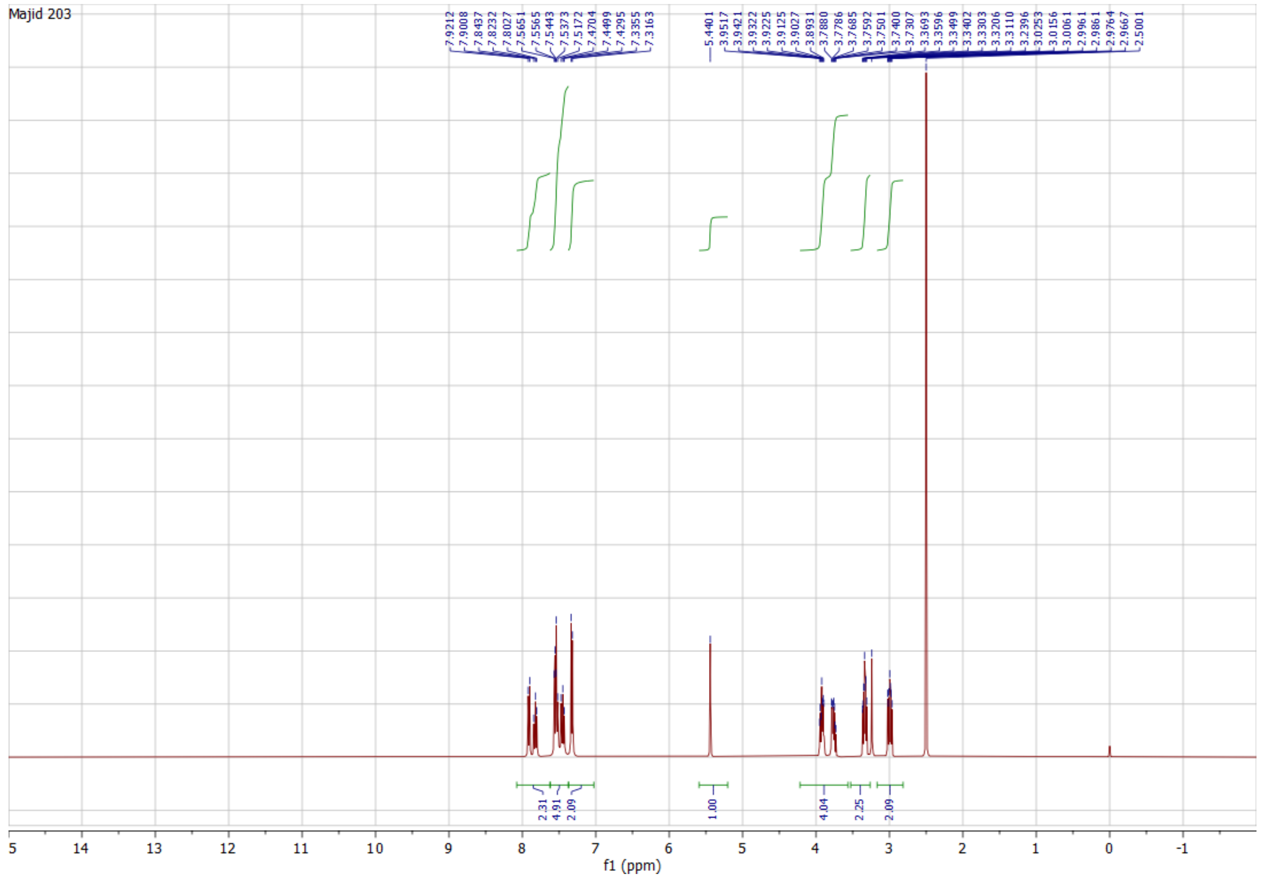

Figure S$_{37}$: ^{1}H-NMR spectra of 3-((4-bromophenyl)(morpholino)methyl)-4-hydroxy-2H-chromen-2-one

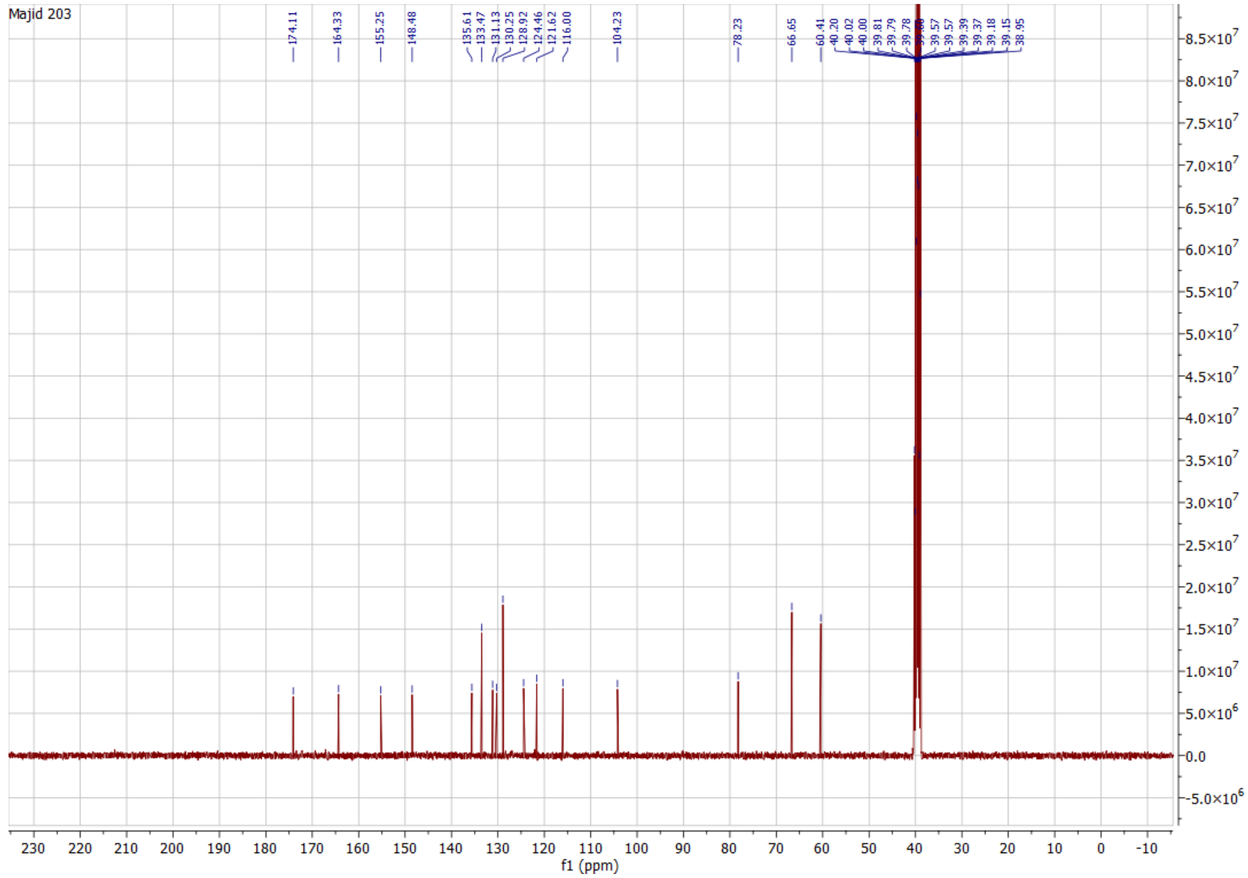

Figure S$_{38}$: ^{13}C-NMR spectra of 3-((4-bromophenyl)(morpholino)methyl)-4-hydroxy-2H-chromen-2-one

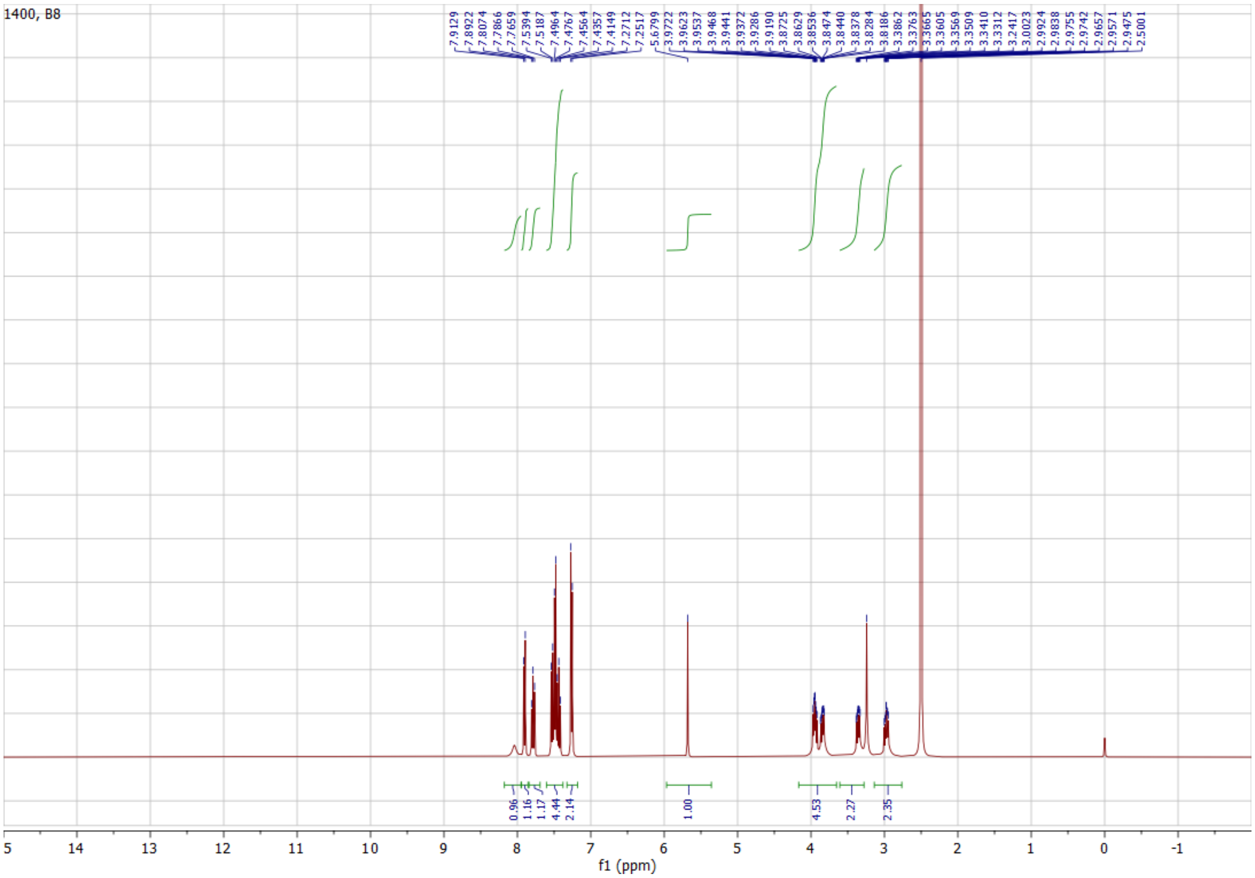

Figure S39: ^{1}H-NMR spectra of 3-((4-chlorophenyl)(morpholino)methyl)-4-hydroxy-2H-chromen-2-one

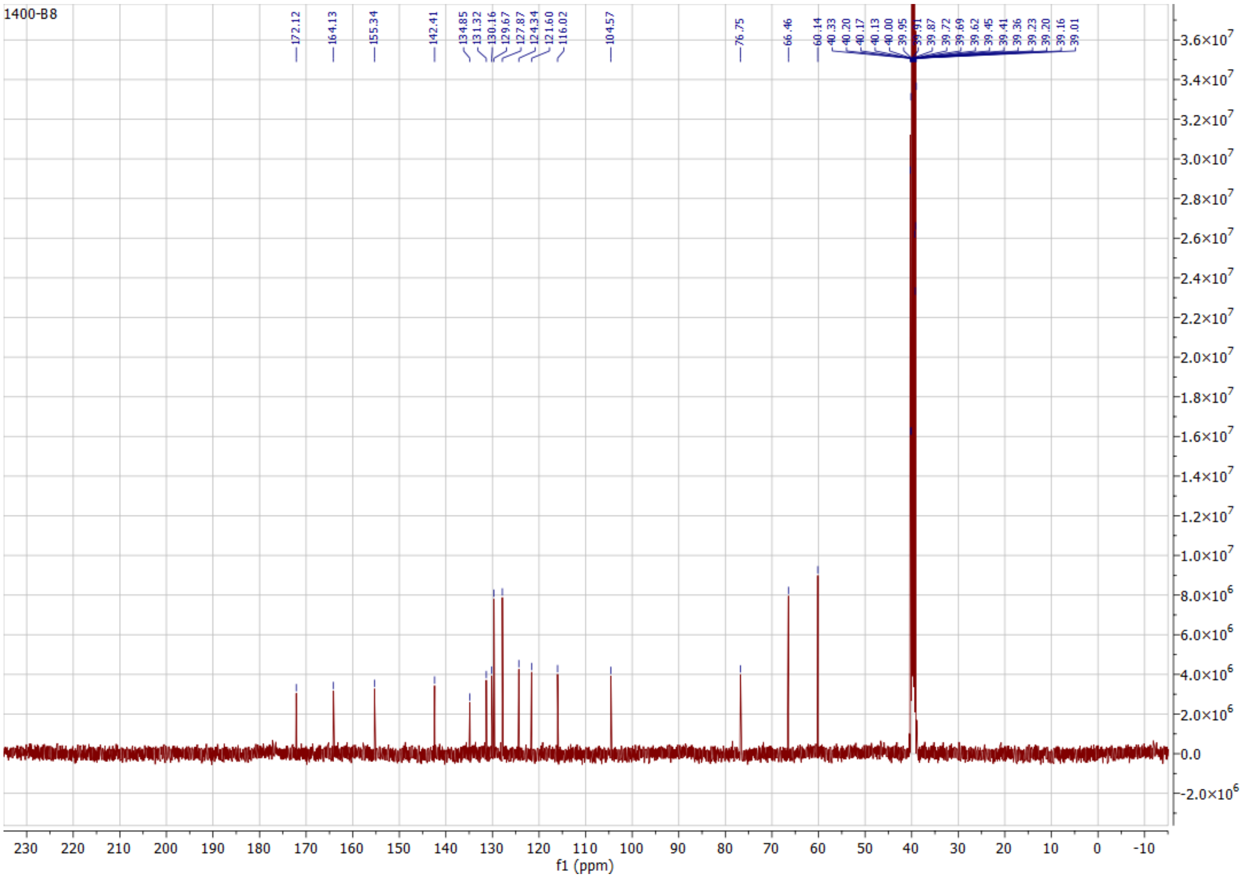

Figure S$_{40}$: ^{13}C-NMR spectra of 3-((4-chlorophenyl)(morpholino)methyl)-4-hydroxy-2H-chromen-2-one

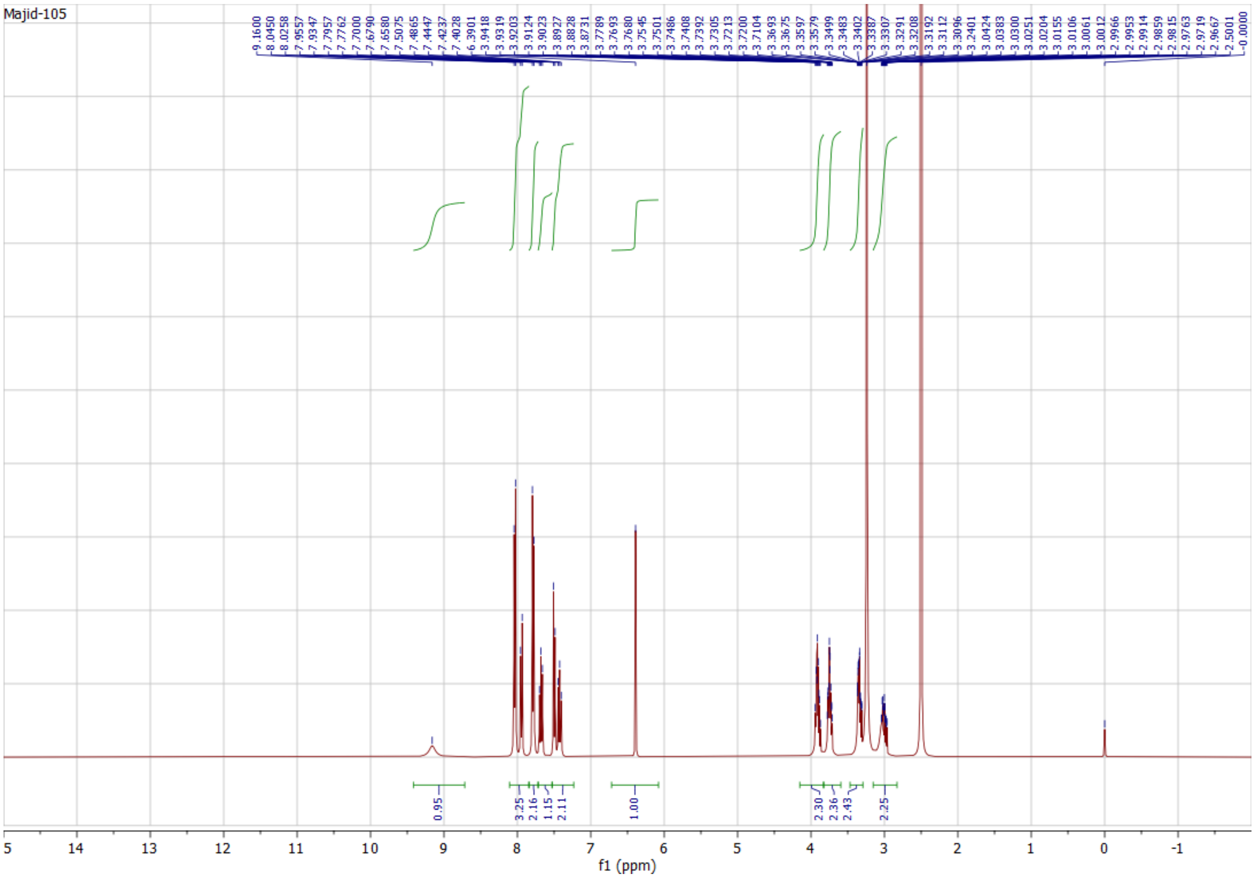

Figure S$_{41}$: ^{1}H-NMR spectra of 4-hydroxy-3-(morpholino(4-nitrophenyl)methyl)-2H-chromen-2-one

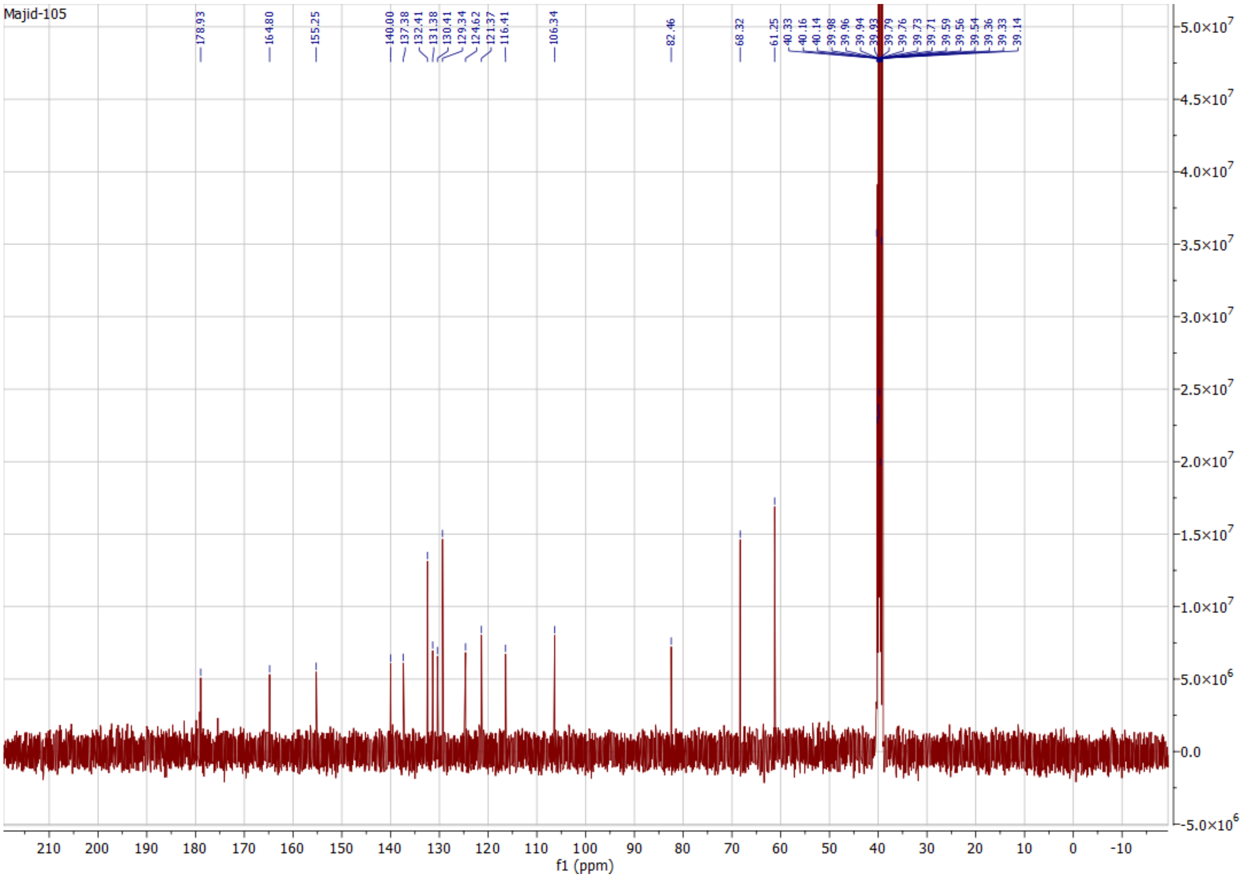

Figure S$_{42}$: ^{13}C-NMR spectra of 4-hydroxy-3-(morpholino(4-nitrophenyl)methyl)-2H-chromen-2-one

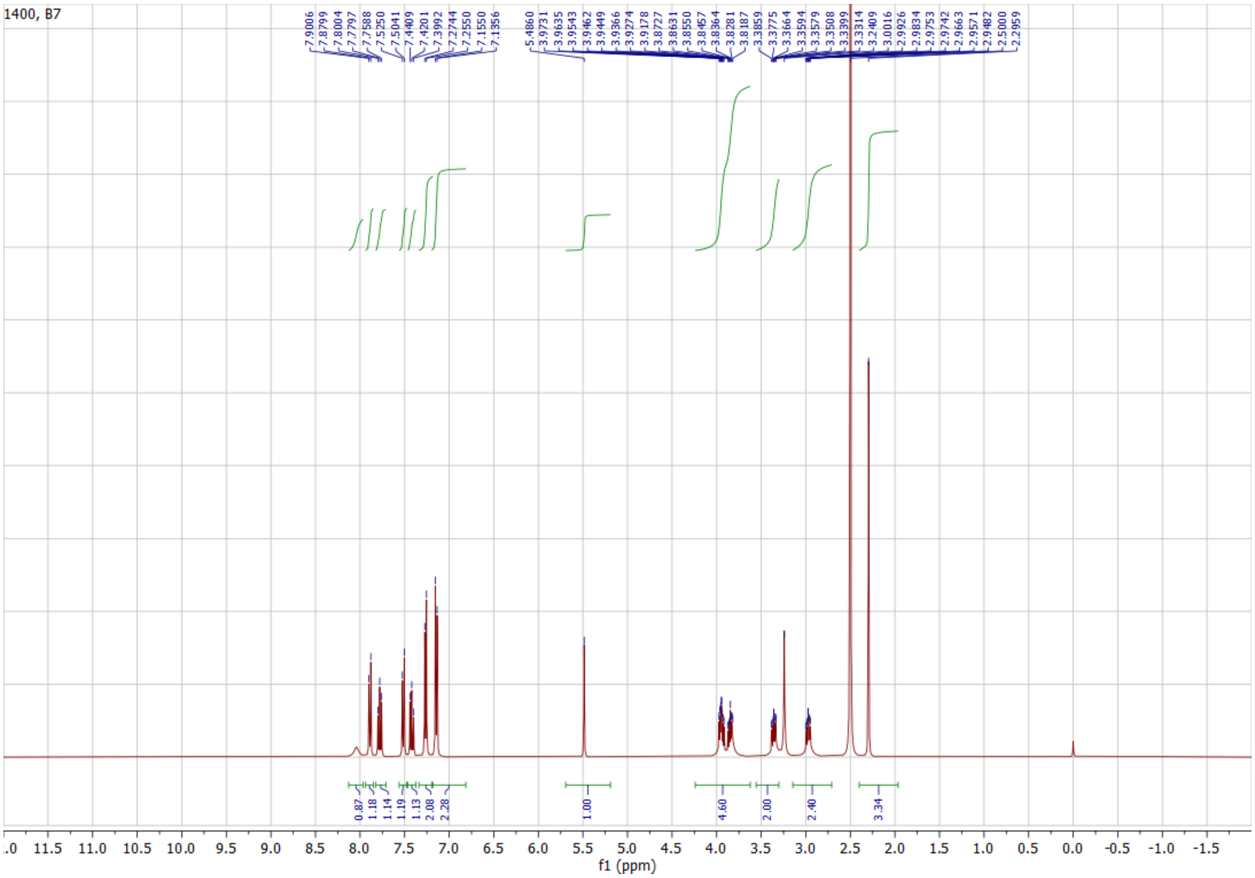

Figure S$_{43}$: ^{1}H-NMR spectra of 4-hydroxy-3-(morpholino(p-tolyl)methyl)-2H-chromen-2-one

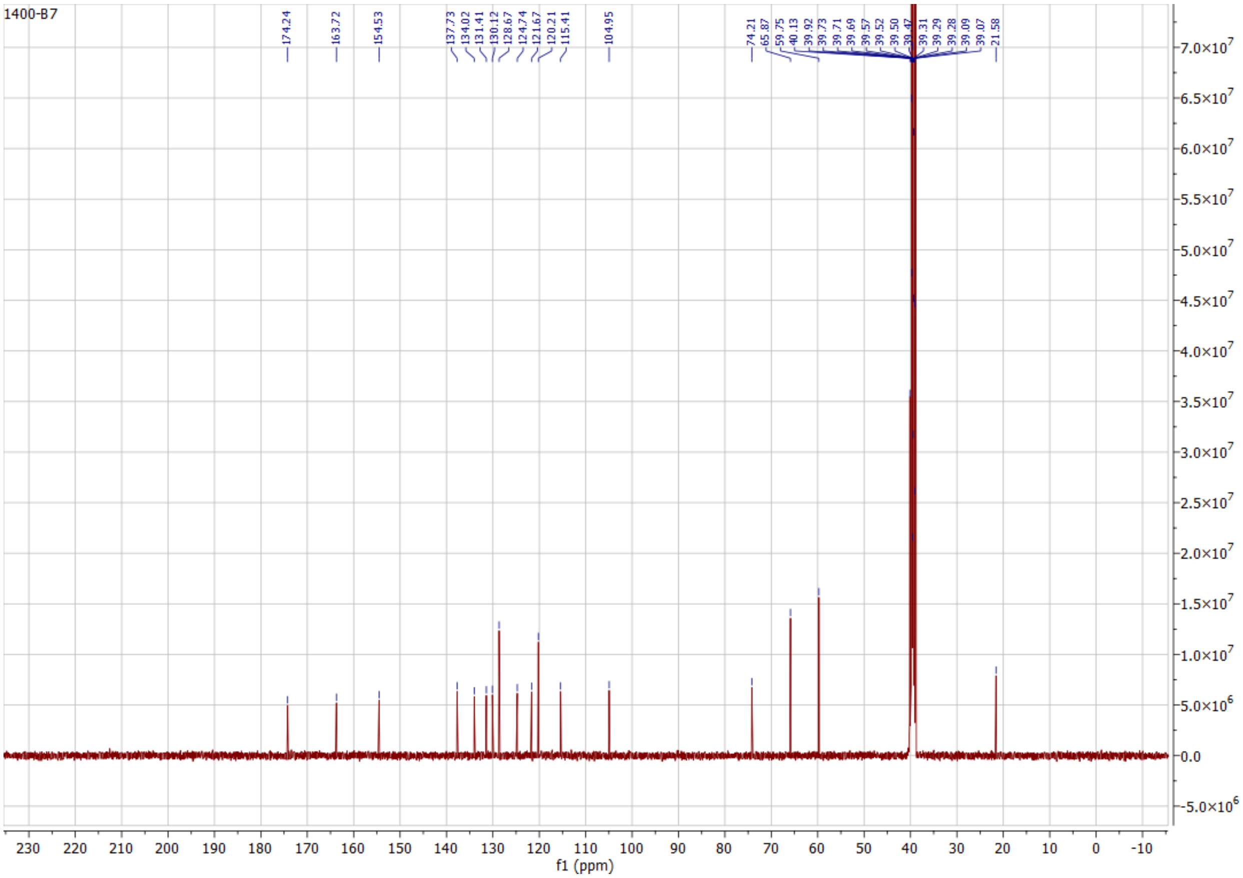

Figure S$_{44}$: ^{13}C-NMR spectra of 4-hydroxy-3-(morpholino(p-tolyl)methyl)-2H-chromen-2-one

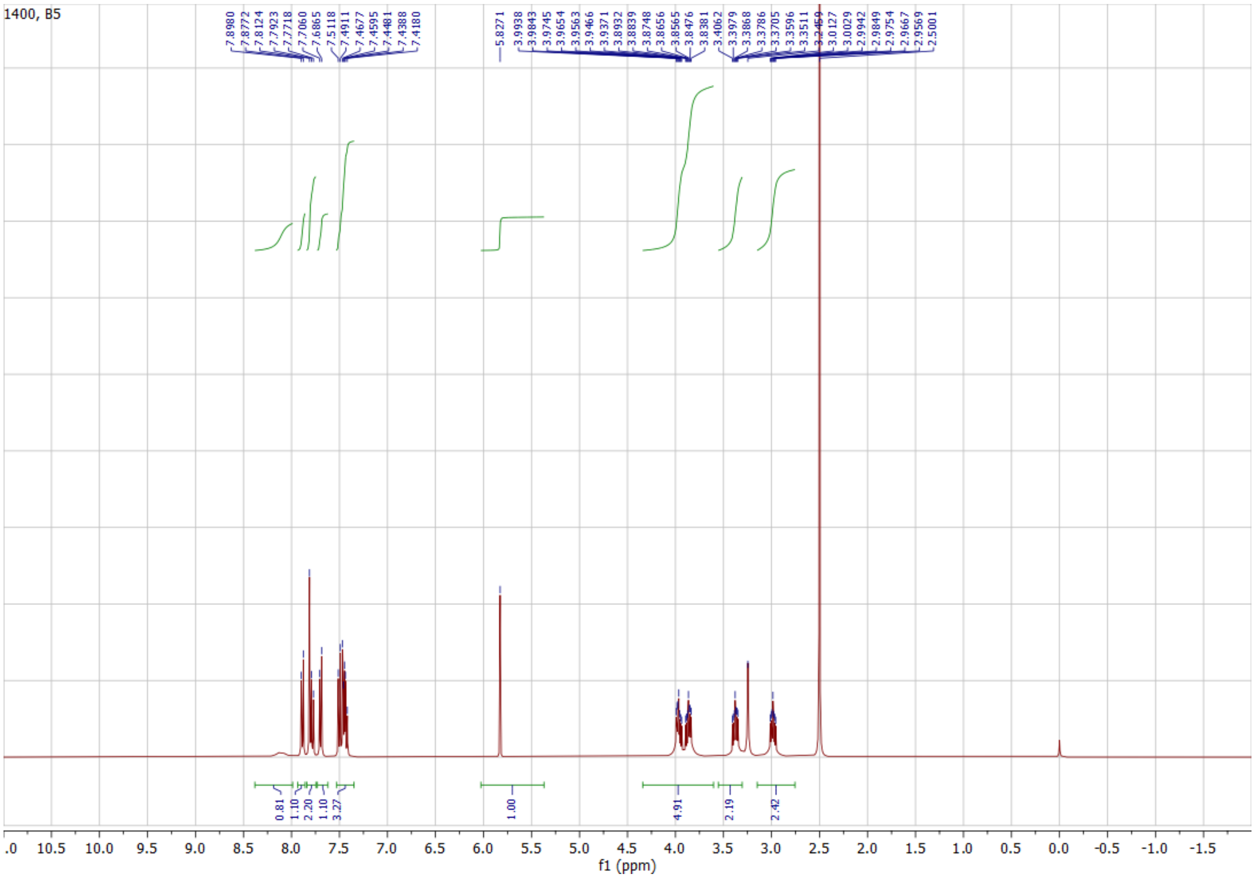

Figure S$_{45}$: ^{1}H-NMR spectra of 3-((2,4-dichlorophenyl)(morpholino)methyl)-4-hydroxy-2H-chromen-2-one

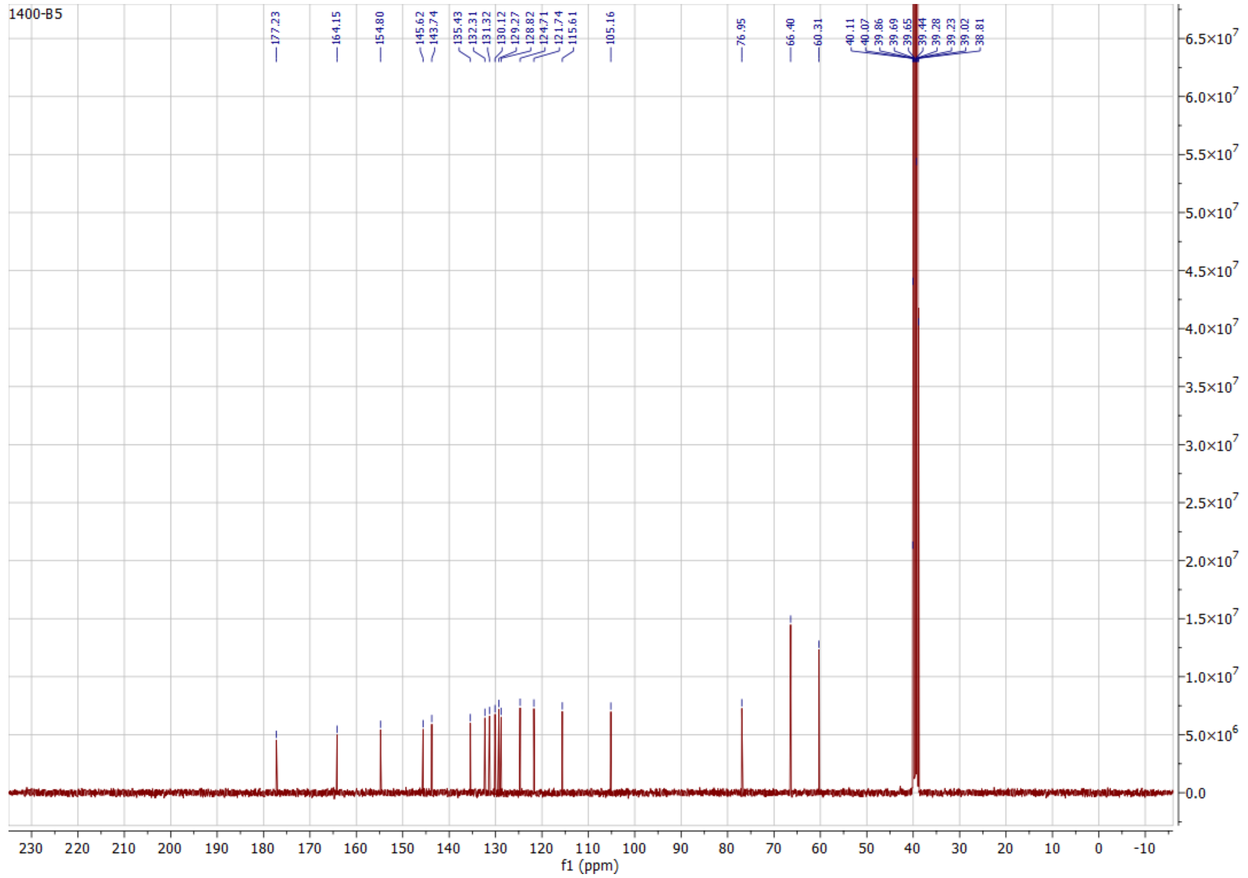

Figure S$_{46}$: ^{13}C-NMR spectra of 3-((2,4-dichlorophenyl) (morpholino)methyl)-4-hydroxy-2H-chromen-2-one

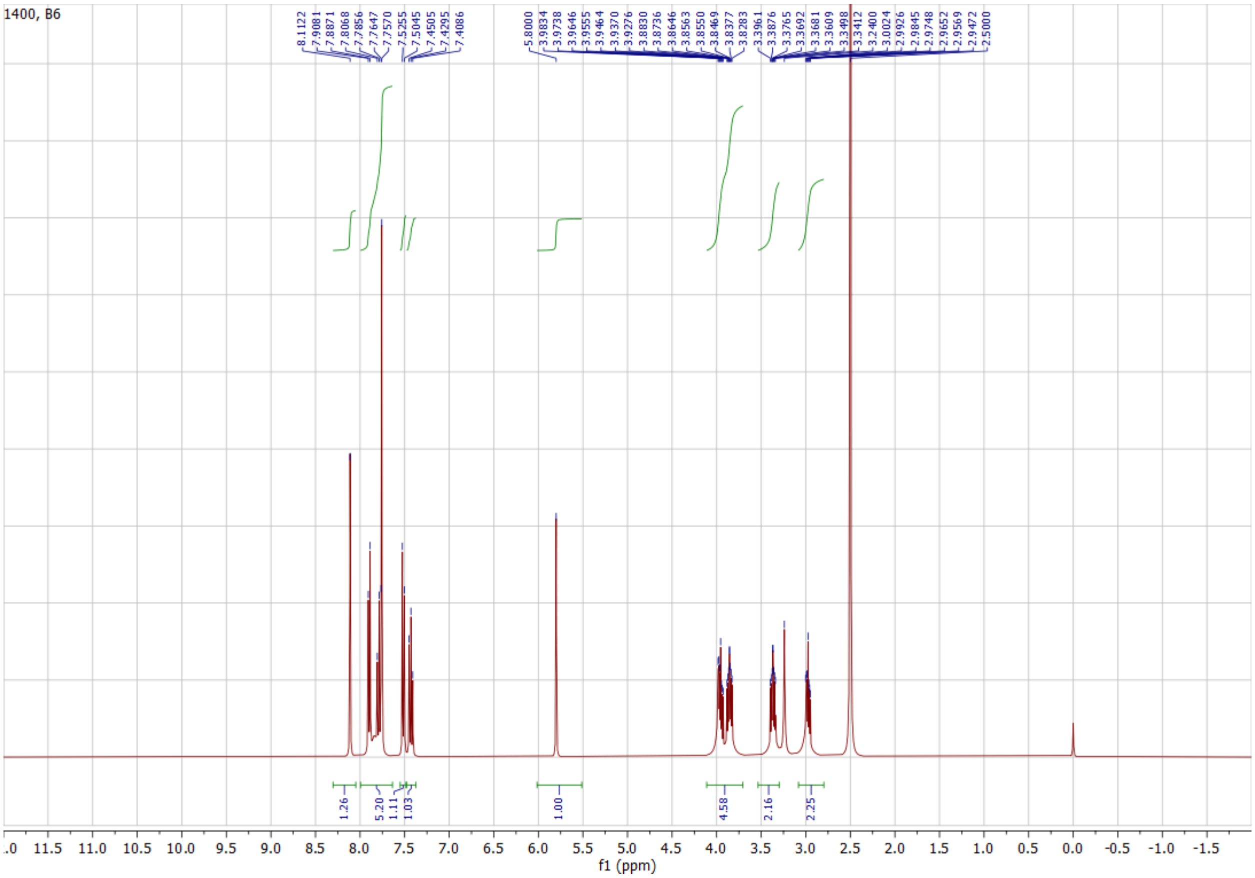

Figure S$_{47}$: ^{1}H-NMR spectra of 3-((3,5-dichlorophenyl) (morpholino)methyl)-4-hydroxy-2H-chromen-2-one

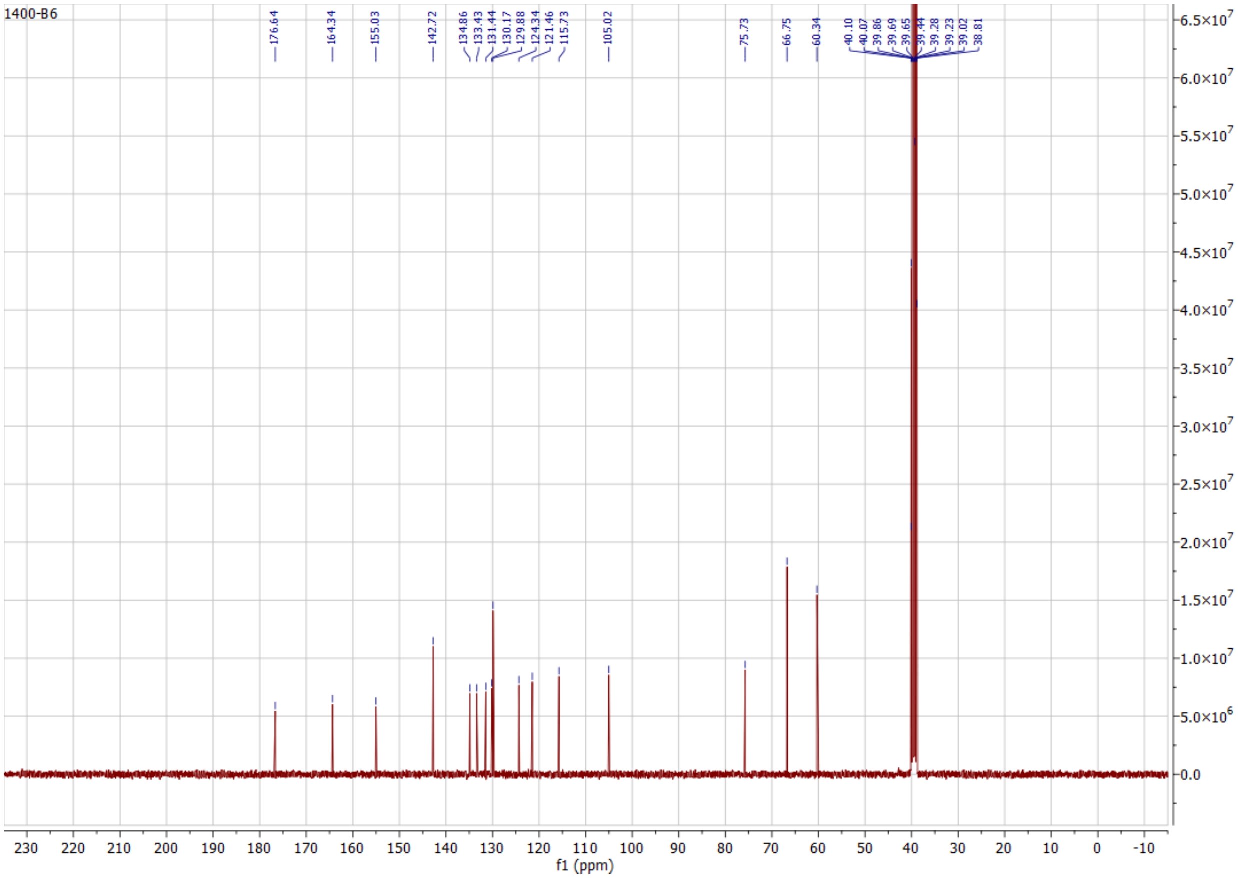

Figure S$_{48}$: ^{13}C-NMR spectra of 3-((3,5-dichlorophenyl) (morpholino) methyl)-4-hydroxy-2H-chromen-2-one
